# Proteome changes in pepper (*Capsicum annuum* L.) leaves induced by the green peach aphid (*Myzus persicae* Sulzer)

**DOI:** 10.1186/s12870-020-02749-x

**Published:** 2021-01-06

**Authors:** Victoria Florencio-Ortiz, Susana Sellés-Marchart, José L. Casas

**Affiliations:** 1 Unidad Asociada CSIC-UA IPAB. Instituto Universitario de Investigación CIBIO (Centro Iberoamericano de la Biodiversidad), University of Alicante, Carretera de San Vicente del Raspeig, s/n, E-03690 San Vicente del Raspeig, Alicante, Spain; 2 Genomics and Proteomics Unit, Servicios Técnicos de Investigación, University of Alicante, Carretera de San Vicente del Raspeig, s/n, E-03690 San Vicente del Raspeig, Alicante, Spain

**Keywords:** Plant proteomics, LC-MS/MS, Plant defense, Biotic stress, Plant-aphid interaction

## Abstract

**Background:**

Aphid attack induces defense responses in plants activating several signaling cascades that led to the production of toxic, repellent or antinutritive compounds and the consequent reorganization of the plant primary metabolism. Pepper (*Capsicum annuum* L.) leaf proteomic response against *Myzus persicae* (Sulzer) has been investigated and analyzed by LC-MS/MS coupled with bioinformatics tools.

**Results:**

Infestation with an initially low density (20 aphids/plant) of aphids restricted to a single leaf taking advantage of clip cages resulted in 6 differentially expressed proteins relative to control leaves (3 proteins at 2 days post-infestation and 3 proteins at 4 days post-infestation). Conversely, when plants were infested with a high density of infestation (200 aphids/plant) 140 proteins resulted differentially expressed relative to control leaves (97 proteins at 2 days post-infestation, 112 proteins at 4 days post-infestation and 105 proteins at 7 days post-infestation). The majority of proteins altered by aphid attack were involved in photosynthesis and photorespiration, oxidative stress, translation, protein folding and degradation and amino acid metabolism. Other proteins identified were involved in lipid, carbohydrate and hormone metabolism, transcription, transport, energy production and cell organization. However proteins directly involved in defense were scarce and were mostly downregulated in response to aphids.

**Conclusions:**

The unexpectedly very low number of regulated proteins found in the experiment with a low aphid density suggests an active mitigation of plant defensive response by aphids or alternatively an aphid strategy to remain undetected by the plant.

Under a high density of aphids, pepper leaf proteome however changed significantly revealing nearly all routes of plant primary metabolism being altered. Photosynthesis was so far the process with the highest number of proteins being regulated by the presence of aphids.

In general, at short times of infestation (2 days) most of the altered proteins were upregulated. However, at longer times of infestation (7 days) the protein downregulation prevailed.

Proteins involved in plant defense and in hormone signaling were scarce and mostly downregulated.

**Supplementary Information:**

The online version contains supplementary material available at 10.1186/s12870-020-02749-x.

## Background

Aphids (Hemiptera: Aphididae) represent one of the most devastating pests to crop production owing to their enormous reproductive potential along with their unique feeding strategy from the phloem. Among the more than 4000 known aphid species, about 250 represent a major threat to agriculture worldwide [1, 2]. They can directly cause damage and lower agricultural yields by depleting photoassimilates and injecting salivary secretions that can be phytotoxic and also affect plant hormone balances changing host metabolism to their advantage and interfering with the plant’s physiological functions [1, 3–5]. The signs and symptoms of aphid attack can be diverse (chlorosis, necrosis, wilting, stunting, and malformation of new growth), therefore, it is likely that host molecular response is specific for a certain plant-aphid interaction [6, 7]. Additionally to their direct effects, aphid honeydew excrement can build enough on plants impairing photosynthesis and promoting the development of fungal diseases [4, 5] and aphids are also vectors of plant viruses, transmitting nearly 30% of all plant virus species described to date [8]. Between all aphid species *Myzus persicae* (Sulzer), the green peach aphid, especially stands out for being highly polyphagous. It feeds on over 400 plant species belonging to nearly 50 plant families, affecting several important agricultural and horticultural crops. Moreover, *M. persicae* is vector for more than 100 viral diseases [9] and is the aphid specie that has developed more mechanisms (at least six types) of insecticide resistance [2]. Thus, the identification of factors that regulate plant resistance or tolerance and limit aphid infestation is momentousness.

Plants have evolved elaborate defense systems to protect themselves against insect herbivores, some of which are expressed constitutively whereas others are induced only after herbivore attack. The herbivore-induced production of defense is initiated by the recognition of insect oral secretions and signals from injured plant cells and then mediated by elaborate signaling networks, which include receptors/sensors, calcium (Ca^2+^) influxes, kinase cascades, reactive oxygen species (ROS), and phytohormone signaling pathways. Plant defense are commonly classified as direct, if they have an effect on the herbivore, or as indirect, if they enhance the attraction of the herbivore’ natural enemies. Between the direct defenses, of extremely importance are the huge diversity of plant metabolites with toxic, repellent or antinutritive properties, like glucosinolates, alkaloids, terpenoids, phenolics or proteinase inhibitors, just to mention some [10–12]. This accumulation of defensive compounds is usually associated with significant alterations in primary metabolism following insect attack, which may serve for the provision of energy, reducing equivalents, and carbon skeletons to support the defense responses [13, 14]. Moreover, the reconfiguration of primary metabolism could support the physiological adjustments that allow the plants to tolerate herbivory reducing the negative impacts of herbivore attack, and also some primary metabolites may have themselves a defensive role [13]. However, as insects may also manipulate plant primary metabolism for their own benefit, it is difficult to establish whether or not the observed changes are adaptive responses of plants [14].

Plant-aphid interactions have been widely studied at the transcriptional level revealing that aphids induce transcriptional reprogramming in their host plants. They modulate plant sequences involved in signaling, protein synthesis, modification and degradation, maintenance of cell structure and homeostasis, photosynthesis and secondary metabolism [15–17]. However, available data indicate poor correlation between transcript levels with those of their respective functional products, the proteins, questioning thus the relevance of using mRNA profiling data to elucidate plant phenotypes [7, 18]. Expression levels of a protein depend not only on transcription rates of the gene, but also on additional control mechanisms, such as mRNA stability, splicing, translational regulation, post-translational processing, control of protein turnover, protein degradation or a combination of these [19, 20]. In this context, proteomics has recently become a complementary tool to transcriptomics in the study of plant-herbivore interactions [20–23]. However, little is still known about the mechanism of plant defense against aphids at a protein level, with some studies limited to a few plant-aphid interactions.

Solanaceae is the third most economically important family in plant kingdom after the Poaceae and Fabaceae. In Solanaceae, proteomics research has been focused on tomato (42%) followed by potato (28%) and tobacco (20%) [18]. Some recent studies have tackled the study of leaf proteome changes in pepper in response to abiotic stress [24], pathogens [25–27], phytohormones [28] and larvae oral secretion [29]. Up to our knowledge, however, a proteomic study of the response of pepper plants against insect herbivory has not been conducted so far. In the present work, we have used a label-free proteomics technology coupled with bioinformatics tools to analyze the leaf proteome responses of sweet pepper (*Capsicum annuum* L.) to *M. persicae* infestation*.* Understanding how the plant responds to aphids at the proteomic level will provide tools for a better management of pests in agriculture.

## Results

**Fig. 1:**
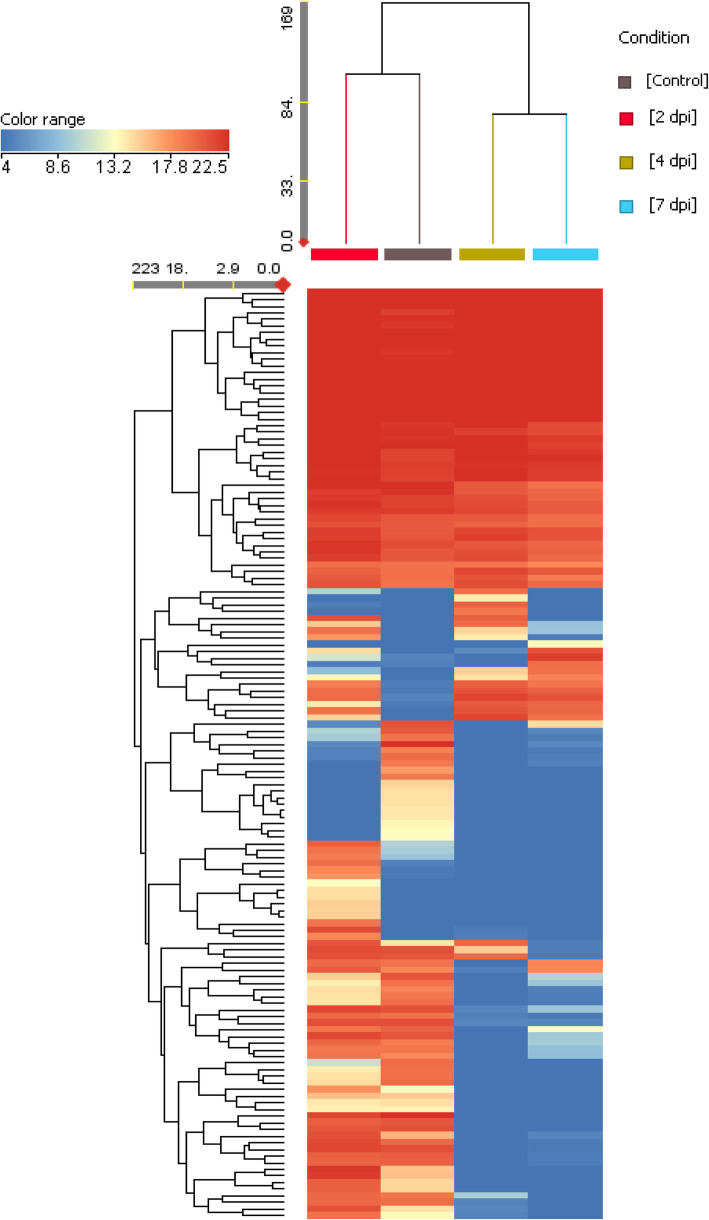
Hierarchical clustering results of regulated proteins in the experiment with high density of infestation. The obtained heat map showed a clustering of the samples coming from 4 different treatments. Each row corresponds to a protein and the complete list of proteins arranged by order of appearance (from top to bottom) is provided in Additional file 3

We studied pepper leaf proteome responses against *M. persicae* infestation following two different approaches. When plants were infested with an initially low density of aphid infestation (20 aphids/plant) and the response was studied at local level taking advantage of clip cages, only 6 proteins resulted differentially expressed relative to control leaves (3 at 2 dpi and 3 at 4 dpi) (Table 1). Conversely, when plants were infested with a high density of aphids (200 aphids/plant) 140 proteins resulted differentially expressed relative to control leaves, during the whole time-course experiment (97 at 2 dpi, 112 at 4 dpi and 105 at 7 dpi) (Table 2, Fig. 1). The complete list of the regulated proteins with the parameters for protein identification, protein abundances and their functional annotations is provided in the Additional file 1 and some characteristic spectra are showed in Additional file 2.

**Table 1:** Regulated proteins in the clip-cages experiment. *N* = 4

**Regulated proteins**				**Time of infestation**		
				**3 hpi**	**2 dpi**	**4dpi**
Total				0	3	3
Upregulated				0	3	0
Downregulated				0	0	3
**Functional category**	**Protein name**	**Accession Number**	**Protein code**	**Fold change (log)**		
Photosynthesis	psbP domain-containing protein 1, chloroplastic isoform X1	XP_016539332.1	A	nc	nc	−19.56
Carbohydrate metabolism	beta-xylosidase alpha-L-arabinofuranosidase 2-like	XP_016539194.1	B	nc	19.89	nc
Lipid metabolism	3-oxoacyl-[acyl-carrier-] synthase chloroplastic	XP_016559523.1	C	nc	18.43	nc
Stress and defence	thioredoxin F- chloroplastic-like	XP_016573859.1	D	nc	nc	−20.92
	thioredoxin reductase NTRB-like	XP_016560325.1	E	nc	18.23	nc
Unknown function	uncharacterized protein LOC107862498	XP_016563584.1	F	nc	nc	−18.95

**Table 2:** Regulated proteins in the high infestation density experiment. *N* = 4

**Regulated proteins**				**Time of infestation**		
				**2 dpi**	**4 dpi**	**7 dpi**
Total				97	112	105
Upregulated				69	44	25
Downregulated				28	68	80
**Functional category**	**Protein name**	**Accession Number**	**Protein code**	**Fold change (log)**		
Photosynthesis	psbP-like protein 1, chloroplastic	XP_016563374.1	1	6.40	−9.36	−14.45
	psbP domain-containing protein 6, chloroplastic	XP_016574890.1	2	−9.73	−19.20	− 14.64
	oxygen-evolving enhancer protein 3–2, chloroplastic	XP_016560066.1	3	−1.25	−22.55	−22.55
	photosystem II reaction center Psb28 protein	XP_016541900.1	4	nc	−20.08	−15.54
	photosystem II CP47 chlorophyll apoprotein (chloroplast)	AFP90802.1	5	1.44	2.17	1.43
	photosystem II protein D1 (chloroplast)	AFP90756.1	6	1.24	2.16	1.40
	chloroplast pigment-binding protein CP26	ACX71300.1	7	1.05	1.26	nc
	uncharacterized protein ycf39	XP_016540029.1	8	14.01	15.48	14.40
	cytochrome f-like	XP_016559008.1	9	1.22	1.51	1.09
	NAD(P)H-quinone oxidoreductase subunit O, chloroplastic	XP_016551962.1	10	−13.10	−13.10	−13.10
	photosystem I P700 apoprotein A2 (chloroplast)	AFP90775.1	11	1.97	3.24	2.54
	photosystem I P700 apoprotein A1 (chloroplast)	AFP90776.1	12	1.10	1.94	1.41
	photosystem I reaction center subunit III, chloroplastic-like	XP_016559311.1	13	1.08	1.49	nc
	photosystem I reaction center subunit VI, chloroplastic-like	XP_016562688.1	14	0.99	1.82	nc
	photosystem I subunit VII (chloroplast)	AFP90827.1	15	1.46	1.21	nc
	ferredoxin, chloroplastic-like	XP_016539055.1	16	−15.49	−20.65	−5.93
	ruBisCO large subunit-binding protein subunit alpha	XP_016547312.1	17	nc	nc	−1.22
	ruBisCO large subunit-binding protein subunit beta, chloroplastic	XP_016579199.1	18	nc	nc	−1.19
	calvin cycle protein CP12–2, chloroplastic	XP_016555606.1	19	−8.54	−19.50	−19.50
	geranylgeranyl diphosphate reductase, chloroplastic	XP_016563245.1	20	nc	1.29	nc
	magnesium-chelatase subunit ChlI, chloroplastic	XP_016545159.1	21	nc	nc	−1.55
	ycf54-like protein	XP_016576775.1	22	nc	−15.36	−15.36
	protein CURVATURE THYLAKOID 1B, chloroplastic	XP_016545573.1	23	4.54	−16.26	−11.23
Amino acid metabolism	glutamate--glyoxylate aminotransferase 2	XP_016547661.1	24	1.04	1.35	nc
	serine--glyoxylate aminotransferase	XP_016550188.1	25	1.19	1.82	1.98
	glycine dehydrogenase (decarboxylating), mitochondrial-like	XP_016550439.1	26	nc	1.13	nc
	5-methyltetrahydropteroyltriglutamate--homocysteine methyltransferase	XP_016555027.1	27	1.00	1.05	nc
	bifunctional aspartokinase/homoserine dehydrogenase 1, chloroplastic-like isoform X1	XP_016569906.1	28	4.62	19.98	nc
	putative ketol-acid reductoisomerase	ACF17638.1	29	nc	1.08	nc
	LL-diaminopimelate aminotransferase, chloroplastic	XP_016578067.1	30	1.05	1.35	nc
	kynurenine formamidase isoform X1	XP_016539519.1	31	−13.70	−18.52	−14.08
	putative ferredoxin-dependent glutamate synthase 1	ACF17655.1	32	1.58	2.47	1.86
	ACT domain-containing protein ACR11-like	XP_016563012.1	33	nc	nc	−1.25
	ACT domain-containing protein ACR12-like isoform X1	XP_016551208.1	34	6.14	−15.72	−15.72
Carbohydrate metabolism	NADP-dependent malic enzyme-like	XP_016543302.1	35	13.51	14.40	18.42
	malate dehydrogenase, chloroplastic-like isoform X1	XP_016566422.1	36	nc	−9.35	−18.99
	enolase	XP_016542903.1	37	nc	nc	−1.03
	fructose-1,6-bisphosphatase, chloroplastic-like	XP_016542325.1	38	1.09	1.71	nc
	glucose-1-phosphate adenylyltransferase small subunit, chloroplastic/amyloplastic	XP_016581019.1	39	1.39	nc	nc
	glyoxylate/succinic semialdehyde reductase 2, chloroplastic	XP_016562555.1	40	9.50	−9.55	−9.55
Lipid metabolism	2-C-methyl-D-erythritol 4-phosphate cytidylyltransferase, chloroplastic	XP_016538918.1	41	nc	−19.01	−10.31
	zerumbone synthase-like isoform X1	XP_016555539.1	42	nc	nc	−1.10
	GDSL esterase/lipase At5g33370-like	XP_016551009.1	43	19.18	14.89	8.94
	BAHD acyltransferase DCR	XP_016564555.1	44	14.49	nc	nc
Hormone metabolism	3-ketoacyl-CoA thiolase 2, peroxisomal	XP_016566389.1	45	−3.86	−18.41	−13.73
	heterodimeric geranylgeranyl pyrophosphate synthase small subunit, chloroplastic	XP_016542878.1	46	−4.54	−18.92	−14.44
Transcription	28 kDa ribonucleoprotein, chloroplastic	XP_016566474.1	47	nc	−14.55	−19.44
	29 kDa ribonucleoprotein B, chloroplastic-like	XP_016543071.1	48	−5.16	−19.40	−14.99
	polyadenylate-binding protein 8-like isoform X1	XP_016539541.1	49	−5.45	−20.62	−10.68
	DEAD-box ATP-dependent RNA helicase 3, chloroplastic	XP_016537495.1	50	nc	−5.66	−16.40
	putative transcription factor Btf3	ABM55742.1	51	−14.41	−14.41	− 14.41
Translation	30S ribosomal protein S9, chloroplastic	XP_016568673.1	52	17.49	14.04	4.42
	30S ribosomal protein S17, chloroplastic	XP_016562689.1	53	−14.19	−14.19	−14.19
	40S ribosomal protein S2–3-like	XP_016569631.1	54	14.63	16.56	15.89
	50S ribosomal protein L21, chloroplastic	XP_016574877.1	55	−18.84	−18.84	−14.04
	60S ribosomal protein L8	XP_016581322.1	56	14.56	5.40	20.82
	60S ribosomal protein L11–1-like	XP_016580888.1	57	nc	2.09	1.29
	60S ribosomal protein L12	XP_016577833.1	58	nc	−20.87	−20.87
	60S acidic ribosomal protein P2-like	XP_016577917.1	59	−15.05	− 15.05	− 15.05
	ribosomal protein L16 (chloroplast)	AIA76997.1	60	nc	−4.71	14.59
	protein MLP1 homolog	XP_016580206.1	61	2.19	−13.42	nc
	elongation factor 1-beta 2-like	XP_016548084.1	62	nc	−20.60	−11.33
	ribosome-recycling factor, chloroplastic isoform X1	XP_016542152.1	63	nc	−20.87	−16.49
	eukaryotic initiation factor 4A-2	XP_016550226.1	64	1.14	1.08	nc
	chloroplast stem-loop binding protein of 41 kDa b, chloroplastic	XP_016578068.1	65	1.18	1.68	0.69
Protein folding and degradation	calnexin homolog 1	XP_016563032.1	66	21.15	4.55	nc
	protein GrpE isoform X1	XP_016543595.1	67	19.06	nc	nc
	chaperone protein ClpB3, chloroplastic	XP_016561338.1	68	14.52	nc	nc
	10 kDa chaperonin-like	XP_016551490.1	69	−14.44	−14.44	−14.44
	trigger factor-like protein TIG, Chloroplastic	XP_016581355.1	70	nc	−14.52	− 14.52
	protein disulfide-isomerase-like	XP_016575805.1	71	nc	nc	−1.29
	leucine aminopeptidase 2, chloroplastic	XP_016540297.1	72	1.53	1.91	nc
	presequence protease 1, chloroplastic/mitochondrial-like	XP_016539607.1	73	15.39	19.59	8.84
	ATP-dependent zinc metalloprotease FTSH, chloroplastic	XP_016568301.1	74	1.00	nc	nc
	proteasome subunit alpha type-4	XP_016560260.1	75	13.31	−4.25	−4.25
	proteasome subunit alpha type-5	XP_016559469.1	76	6.46	5.56	−9.61
	proteasome subunit alpha type-6	XP_016540329.1	77	−10.53	−20.44	−15.25
	Skp1	AAX83944.1	78	14.60	−4.82	−4.82
	serine protease inhibitor 5-like	XP_016563917.1	79	nc	13.96	nc
Stress and defence	putative 13-lipoxygenase	ADZ73653.1	80	1.27	1.70	1.15
	glutathione S-transferase, partial	ACN60408.1	81	nc	−19.12	nc
	uncharacterized monothiol glutaredoxin ycf64-like	XP_016560321.1	82	15.18	nc	nc
	thioredoxin	AAR83852.1	83	nc	−15.85	−16.06
	peroxidase 12-like	XP_016569419.1	84	13.12	nc	nc
	thioredoxin-like protein CDSP32, chloroplastic	XP_016568326.1	85	nc	nc	−1.18
	2-Cys peroxiredoxin BAS1, chloroplastic-like	XP_016543590.1	86	nc	nc	−1.11
	peroxiredoxin-2E-2, chloroplastic	XP_016543463.1	87	nc	nc	−1.81
	peptide methionine sulfoxide reductase A1-like	XP_016564938.1	88	−14.65	−19.45	− 14.86
	putative lactoylglutathione lyase isoform X1	XP_016537843.1	89	5.68	−8.60	−13.50
	stromal 70 kDa heat shock-related protein, chloroplastic	XP_016538990.1	90	nc	nc	−1.38
	heat shock protein 90–5, chloroplastic	XP_016547276.1	91	1.32	nc	nc
	hsp70-Hsp90 organizing protein 2-like	XP_016568921.1	92	nc	−19.97	−6.85
	kirola	XP_016552266.1	93	1.16	−18.60	−9.15
	fatty acid hydroperoxide lyase	AAK27266.1	94	8.53	15.13	19.29
	CBS domain-containing protein CBSX3, mitochondrial	XP_016541123.1	95	nc	−13.79	−13.79
	peroxisomal (S)-2-hydroxy-acid oxidase GLO1	XP_016552561.1	96	nc	1.40	nc
	dihydrolipoyl dehydrogenase 2, chloroplastic-like isoform X4	XP_016573540.1	97	9.98	19.29	nc
Cell organization	histone H3-like protein	AAR84425.1	98	6.47	−4.92	16.80
	membrane-associated protein VIPP1, chloroplastic	XP_016551679.1	99	nc	−20.56	−20.56
Transport	coatomer subunit gamma	XP_016550178.1	100	20.96	20.04	nc
	signal recognition particle 43 kDa protein, chloroplastic	XP_016561463.1	101	13.86	−4.29	−4.29
	protein TIC110, chloroplastic	XP_016539169.1	102	nc	−14.53	−14.93
	patellin-3-like	XP_016556342.1	103	nc	nc	13.46
Energy production	ATP synthase CF0 subunit I (chloroplast)	AFP90762.1	104	1.11	nc	−1.32
	ATP synthase delta chain, chloroplastic	XP_016551634.1	105	nc	−3.13	−4.37
	ATP synthase subunit gamma, mitochondrial	XP_016563339.1	106	4.71	−12.97	−12.97
	V-type proton ATPase subunit B2	XP_016539985.1	107	nc	nc	−1.10
	V-type proton ATPase subunit E	XP_016566244.1	108	5.33	−14.86	−14.86
	adenylate kinase 4	XP_016563818.1	109	−5.41	−19.35	−19.35
	cytochrome b5-like	XP_016538256.1	110	1.05	−18.07	−9.43
	cytochrome b561 and DOMON domain-containing protein At3g25290-like	XP_016541279.1	111	18.11	4.62	nc
Miscellaneous	mitochondrial dicarboxylate/tricarboxylate transporter DTC	XP_016582458.1	112	19.17	20.75	19.83
	early nodulin-like protein 2	XP_016541659.1	113	15.05	nc	nc
	remorin	XP_016564655.1	114	−13.31	−13.31	−13.31
	plasma membrane-associated cation-binding protein 1	XP_016545329.1	115	−14.55	−14.55	− 14.55
	uncharacterized protein LOC107845278	XP_016544993.1	116	−13.63	−13.63	− 13.63
	2-methyl-6-phytyl-1,4-hydroquinone methyltransferase, chloroplastic-like	XP_016570658.1	117	15.10	16.35	15.71
	ferritin-2, chloroplastic-like	XP_016575544.1	118	nc	−15.79	−11.72
	bifunctional purple acid phosphatase 26-like	XP_016556756.1	119	nc	18.86	nc
	adenine phosphoribosyltransferase 1-like	XP_016568861.1	120	1.64	−19.56	−15.16
	haloacid dehalogenase-like hydrolase domain-containing protein At4g39970	XP_016552268.1	121	nc	nc	−1.02
	sulfite reductase 1	XP_016554536.1	122	9.56	15.87	15.35
	UPF0603 protein At1g54780, chloroplastic	XP_016538515.1	123	1.23	nc	nc
	peptidyl-prolyl cis-trans isomerase CYP26–2, chloroplastic-like	XP_016556190.1	124	−5.41	−19.23	−10.14
	peptidyl-prolyl cis-trans isomerase CYP38, chloroplastic	XP_016561540.1	125	1.66	nc	nc
	photosynthetic NDH subunit of lumenal location 4, chloroplastic	XP_016551803.1	126	15.07	nc	nc
	COBW domain-containing protein 1	XP_016562950.1	127	nc	nc	−16.25
	nucleoid-associated protein At4g30620, chloroplastic-like	XP_016540291.1	128	−4.84	−19.20	−19.20
	thylakoid lumenal 16.5 kDa protein, chloroplastic	XP_016540179.1	129	nc	−20.34	−15.88
	calmodulin-7-like	XP_016577941.1	130	5.20	−14.88	−14.88
	14–3-3 protein 6-like	XP_016572083.1	131	6.58	−15.54	−15.54
	plasminogen activator inhibitor 1 RNA-binding protein-like isoform X1	XP_016570399.1	132	−18.95	−18.95	− 18.95
Unknown function	uncharacterized protein LOC107880062	XP_016582471.1	133	nc	−1.88	−2.54
	uncharacterized protein OsI_027940	XP_016556193.1	134	10.11	−9.94	−9.94
	uncharacterized protein LOC107850455	XP_016550473.1	135	9.72	−8.92	−8.92
	uncharacterized protein LOC107867216	XP_016568855.1	136	−4.84	−19.52	−19.52
	uncharacterized protein LOC107874389	XP_016576670.1	137	−18.33	−23.49	−18.19
	uncharacterized protein LOC107839055	XP_016537888.1	138	14.98	21.39	19.15
	uncharacterized protein LOC107860356	XP_016561166.1	139	−14.18	−14.18	−14.18
	uncharacterized protein LOC107873810	XP_016576233.1	140	−17.34	−17.34	− 17.34

**Fig. 2:**
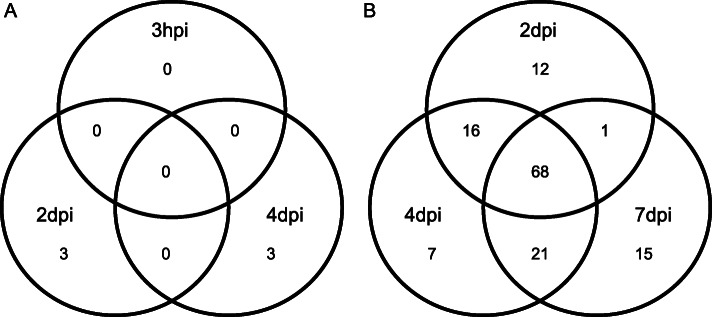
Venn diagram showing the number of overlapping and unique proteins under aphid infestation. **a** Experiment with 20 aphids/leaf confined into clip-cages. **b** Experiment with high density of infestation (200 aphids/plant). *N* = 4

**Fig. 3:**
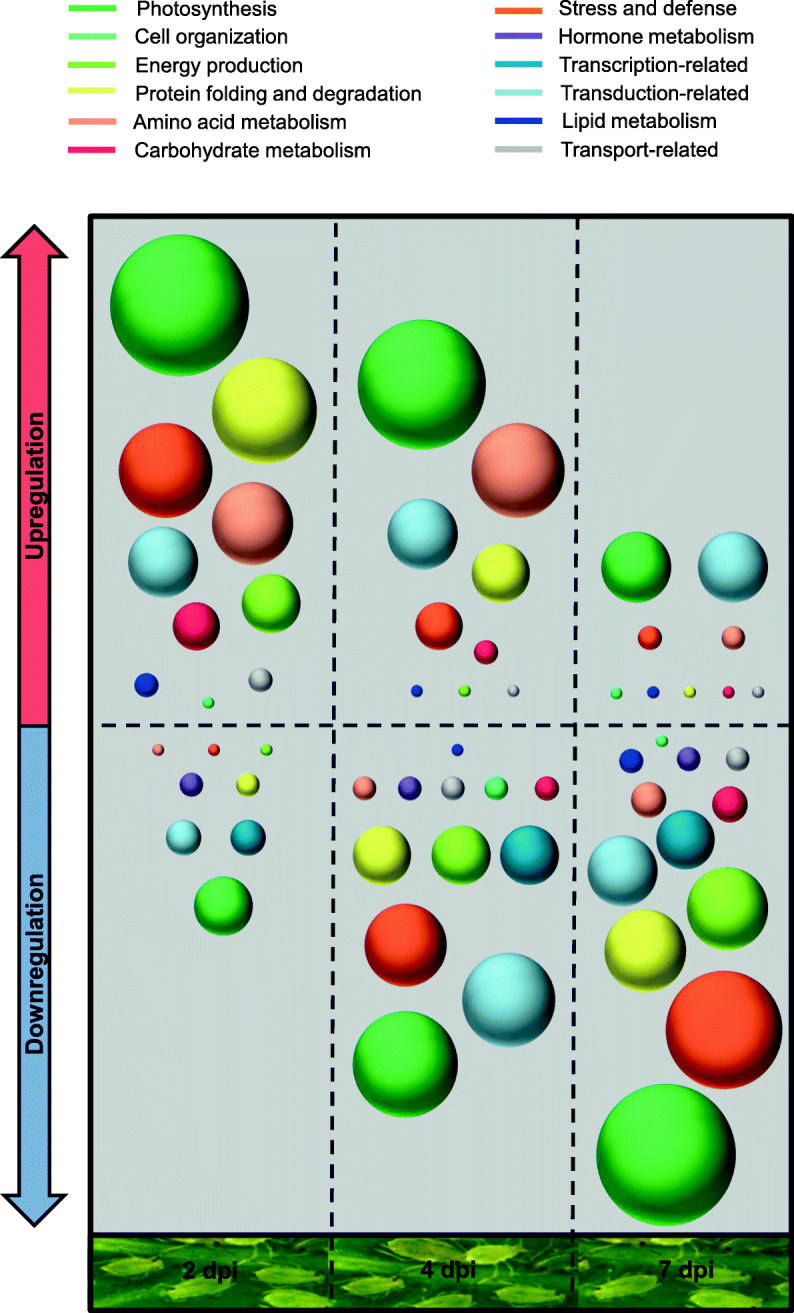
Evolution of the induction / suppression of metabolic pathways upon aphid infestation. The sphere’s diameters are proportional to the number of regulated proteins

A general pattern was found in the present study with most proteins being upregulated at short times but downregulated with the progress of infestation, at both low and high aphid density (Tables 1 and 2). Moreover, there was poor overlapping between the proteins regulated at each time point of infestation (Fig. 2). In the case of leaf discs any of the regulated proteins was shared between different time points of the experiment (Fig. 2a) whereas in the case of a high density of aphid infestation less than half of the proteins (48.6%) were significantly affected throughout the whole experiment (Fig. 2b). The central time point (4 dpi) was the one showing a higher number of regulated proteins (Fig. 2b), and it also represents an intermediate situation in the proteomic response, with more or less equal number of proteins shared with the shorter (2 dpi) and longer (7 dpi) time of aphid infestation. Most of the regulated proteins matched the functional categories of photosynthesis, stress and defense, translation, protein folding and degradation and amino acid and carbohydrate metabolism (Tables 1 and 2). A schematic representation of the time course of induction / suppression of metabolic pathways upon aphid infestation is provided in Fig. 3.

## Discussion

The two approaches differing in aphid infestation assayed in the present study gave very different results. A low aphid density locally confined to a specific leaf area with the use of clip cages provoked minor local variations in the proteome of these plant cells. This absence of a significant plant response is in line with previous behavioural evidences of local reduction of plant resistance by aphids [30, 31] and may be consequence of an active mitigation of plant defensive response. For instance, an immune-suppressive aphid saliva protein was shown to be delivered in mesophyll cells near aphid stylets but not in cells further away from aphid feeding sites [32]. Moreover, aphids continuously secrete saliva into the plant tissue, which contains multiple types of proteins that facilitate stylet penetration and divert or suppress plant defenses to maintain their successful feeding [1, 3, 33, 34]. Alternatively, aphids may go unnoticed by the plant derived from their “stealthy” mode of feeding which involves only limited plant tissue damage, as they penetrate plant tissue following the apoplastic path to establish feeding sites in the phloem sieve elements.

Conversely, a high density of infestation triggered a large proteome alteration of pepper leaves. It is however worth noting that in this case all the leaves of the plant were infested and collected for the analysis and thus proteome changes were studied at systemic level. This factor that may have contributed to the differences observed in both experiments.

In our study, most of the proteins were only transiently regulated. This highlights the relevance of including time-course experiments when studying plant-insect interactions at a proteomic level. The observed dynamic protein expression pattern can be ascribed to the cascade of events that follows insect attack and that involves an integrative crosstalk between signaling molecules, such as Ca^2+^, ROS, protein kinases and phytohormones [35, 36]. In addition, it may also reflect the adjustment of plant defense responses to handle the progression of a successful infestation and/or the ability of aphids to suppress some plant defense responses during long-term feeding [7, 37].

In the following sections we will summarize the changes observed in the pepper leaf proteome according to their possible role in plant-aphid interactions. Discussion of the induction or suppression of the different metabolic pathways upon aphid infestation is based on the abundances of a group of proteins belonging to such metabolic pathway. However, further functional analysis would be necessary to confirm the role of specific proteins in plant defense against aphids.

### Photosynthesis and photorespiration

Although there are some well documented exceptions, a general pattern of decreased photosynthesis in response to insect feeding has been observed, supported by actual measurements of changes in photosynthesis rate, photosynthesis-related gene expression, or production of proteins that are part of the photosynthetic apparatus [14]. Moreover, given that removal of leaf material is not necessary and the jasmonic acid (JA) signaling pathway is required for the reduction in photosynthetic activity, it seems to be an active plant response during insect herbivory rather than just a side effect of a metabolic limitation [15, 38]. In the present study, almost the half (47.9%) of the regulated proteins were chloroplastic (Table 2; Additional file 1). Most of the proteins constituents of the electron transport chain (Prot. 5–9, 11–15) were upregulated in response to aphid feeding from 2 to 7 dpi. Interestingly, maintenance or upregulation of photosynthetic capacity via upregulation of photosystem components has been related with wheat tolerance to the russian wheat aphid (*Diuraphis noxia* Mordvilko) [39, 40]. However, we detected that components of the oxygen evolving complex or water-splitting complex (Prot. 1–3) and a subunit of the reaction center of photosystem II (Prot. 4) were downregulated in aphid-infested plants especially between 4 and 7 dpi, when also two subunits of the chloroplastic ATP synthase (Prot. 104, 105) were downregulated. Additionally, one protein of the oxygen evolving center was downregulated at 4 dpi in leaf discs (Prot. A). Overall, although additional functional studies are necessary to reveal the impact of these changes in photosynthetic rates, results suggest that the processes of photooxidation of water, transference of energy and electrons and photophosphorylation (which take place during the light reactions of photosynthesis) may be impaired as a consequence of aphid infestation.

Gas exchange measurements coupled with fluorescence techniques demonstrated that aphid feeding reduces photosynthetic rates in barley through their negative impact in carbon-linked/dark reactions, specifically ribulose-1,5-bisphosphate carboxylase/oxigenase (rubisco) activity and ribulose-1,5-bisphosphate regeneration [41]. Rubisco is the major protein that mediates carbon utilization through the Calvin-Benson cycle or photorespiration. In contrast to other studies showing rubisco up- or downregulation in response to aphid feeding [42–44], we did not detect changes in rubisco content throughout the whole time-course of the experiment. However, proteins implicated in rubisco assembly (Prot. 17, 18) were downregulated at 7dpi, suggesting a possible reduction in rubisco activity after long times of aphid infestation. Photorespiration was traditionally considered as a wasteful process evolved as a way to detoxify 2-phosphoglycolic acid produced by the failed oxygenation rubisco reaction and to recycle carbon to fuel the Calvin-Benson cycle. However, accumulating evidence point to an essential role of photorespiration pathway for plant growth and development and responses to abiotic and biotic stresses, given that it interacts with several primary metabolic pathways [45, 46]. In the present study we detected that some proteins involved in photorespiration (Prot. 24–26, 35) were upregulated in aphid-infested plants. Conversely, a CP12 protein (Prot. 19) was strongly downregulated, especially at longer times of infestation. CP12 is a key protein in the regulation of the Calvin-Benson cycle, as it coordinates the reversible inactivation of the enzymes glyceraldehyde-3-phosphatedehydrogenase (GAPDH) and phospho-ribulokinase (PRK) through the formation of the supramolecular complex GAPDH/CP12/PRK [47]. Collectively, these results suggest a dynamic and complex regulation of photosynthesis and photorespiration in response to aphid feeding, which may be ascribed to the reallocation of plant metabolites from normal growth and reproductive processes to defensive functions in response to aphid attack.

### Amino acid and carbohydrate metabolism

Aphids need to ingest large volumes of phloem sap to fulfil their dietary requirements which, in turn, may modify source-sink relationships and water relations within the plant [4, 17]. Moreover, aphids may be able to alter plant metabolism to adapt phloem sap composition to their own benefit through the induction of enzymes involved in carbon (C) and nitrogen (N) assimilation and mobilization [17, 48, 49]. Accordingly, in the present study enzymes related with amino acid and carbohydrate metabolism were highly represented between those proteins regulated in response to aphid attack (Table 2). Amino acids play a dual role in plant-aphid interactions as major growth-limiting nutrients for insects and as precursors for the production of many plant defense compounds [14]. In a previous study we showed a large increase in the free amino acid content of pepper leaves in response to a high aphid density of infestation [50]. Accordingly, several enzymes involved in the biosynthesis of serine, glycine, methionine, lysine, valine, leucine, isoleucine, threonine and glutamate (Prot. 24–30, 32) were upregulated in aphid-infested plants in the present study.

Aphid feeding results in the activation of a premature leaf senescence characterized by the expression of senescence associated genes, chlorosis and cell death [51]. Induction of senescence has been suggested to be an aphid strategy to provoke the release of free amino acids into the phloem and/or manipulate the sink-source relationship to allow more N transport into infested leaves [3, 14]. However, it could also be a process engaged by the plant to counter the aphids’ ability to alter resource allocation and thereby control severity of aphid infestation [9]. In contrast with Carrillo et al. [48] we did not detect induction of senescence-associated proteins in aphid-infested plants. However, we detected some phenotypical changes that may be related with this process. During senescence a set of coordinated sequential events takes place including the degradation of macromolecules, decrease in the overall protein anabolism, reallocation of nutrients and dismantling of chloroplasts [52]. As chloroplasts represent the main source of N-containing molecules within the leaf it is not surprising that the earliest structural, biochemical and metabolic changes are observed in these organelles [52]. In the present study, the membrane-associated protein VIPP1 (Prot. 99) was strongly downregulated from 4 dpi, which may be indicative of the dismantling of chloroplasts given that it plays a key role in the biogenesis and maintenance of chloroplast membranes [53]. Moreover, 57.1% of the regulated proteins were downregulated at 7 dpi and almost half of them (46.3%) are chloroplastic (Table 2; Additional file 1). Interestingly, all the proteins involved in transcription (Prot. 47–51) and post-transcriptional control (Prot. 132) were downregulated, and also some of the ribosomal proteins (Prot. 53, 55, 58, 59, 62, 63). Three proteases (Prot. 72–74) and one subunit of the proteasome system were upregulated at 2 and 4 dpi. However, all the detected components of the proteasome system (Prot. 75–77) resulted downregulated at 7dpi along with the S-phase kinase-associated-protein 1 (Skp1, Prot. 78), which is necessary for ubiquitin-mediated protein catabolic process. Additionally, a serine protease inhibitor was upregulated at 4dpi (Prot 79). Thus, it seems that proteolysis of endogenous proteins is closely regulated in response to aphid infestation, which has been suggested to contribute to multiple levels of plant defense [42].

As a consequence of protein catabolism, toxic ammonium is released, which has to be immediately re-assimilated into organic molecules via the glutamine synthetase (GS) / glutamine oxoglutarate aminotransferase (GOGAT) pathway [52, 54]. An increase in N assimilation rate is most likely to require enhanced flux through respiratory pathways, since early steps of the tricarboxylic acid (TCA) cycle are the major source of C skeletons for GOGAT [54]. Accordingly, a NADP-dependent malic enzyme, involved in the TCA cycle, was upregulated in aphid-infested plants (Prot. 35) along with the mitochondrial dicarboxylate/tricarboxylate transporter DTC (Prot. 112), which transports TCA cycle intermediates and is also involved in the production of glycerate during photorespiration and ammonium assimilation [55]. The GS/GOGAT system is widely accepted as occupying a central position in leaf N metabolism, however, its regulation at the post-transcriptional level is poorly understood [56]. Interestingly, we found that ACR11 and ACR12 (Prot. 33 and 34) were downregulated from 4 dpi onwards. These proteins have been shown to enhance the activity of GS2 in *Arabidopsis* and to interact and stabilize ferredoxin (Fd)-GOGAT1 possibly modulating its activity [56, 57]. Moreover, ACR11 has shown to regulate ROS and SA accumulation in *Arabidopsis* and play a role in pathogen resistance [58].

### Lipid and hormone metabolism

Besides functioning as major structural components of cell membranes, plant lipids are also precursors of antibiotic compounds and signaling molecules [9, 59]. In the *Arabidopsis-M. persicae* system, PHYTOALEXIN DEFICIENT 4 (PAD4, which encodes a lipase-like protein) is required for antibiosis and antixenosis and *MYZUS PERSICAE*-INDUCED LIPASE 1 (MPL1, which exhibits lipase activity) is required only for antibiosis [9, 60]. Interestingly, a GDSL esterase/lipase (Prot 43) was strongly upregulated in aphid-infested plants from 2 dpi onwards. Some GDSL lipases have shown to regulate plant immunity [61, 62] and, specifically in pepper, GDSL-lipase 1 is involved in signaling pathway of methyl jasmonate (MeJA) and/or the wound responses through pathogenesis-related protein 4 (PR-4) expression modulation [63]. Also, an enzyme linked with the biosynthesis of fatty acids (Prot C) was upregulated in leaf discs at 2 dpi. Plant defense against aphids is enhanced by the disruption of function of the ω-3 FATTY ACID DESATURASE7 (FAD7) [64] but by the expression of α-DIOXYGENASE 1 [65], which highlights the relevance of fatty acid metabolism in plant defense responses to aphids. Conversely, an enzyme related with the isoprenoid biosynthetic process (Prot. 41) was strongly downregulated at 4 and 7 dpi.

Plant responses to aphids appear to be regulated by the signaling pathways driven by JA, salicylic acid (SA), ethylene (ET), abscisic acid (ABA) and gibberellins (GAs) [9, 66]. However, the “decoy” hypothesis has suggested that aphids manipulate plant defense responses through pathway cross-talk to repress the potentially more biologically effective JA-signaling pathway [17]. JA is synthesized through the octadecanoid pathway which is initiated in the chloroplast with the oxygenation of the fatty acid linoleic acid by lipoxygenase (LOX). In our study, 13-LOX (Prot. 129) was upregulated from 2 dpi to 7 dpi but peroxisomal 3-ketoacyl-CoA thiolase 2 (Prot. 73), which participates in further steps of JA biosynthesis, was strongly downregulated from 2 dpi onwards. According to our results, the enzyme allene oxide synthase (which also acts downstream LOX) resulted downregulated in aphid-infested wheat plants [42]. Moreover, a protein involved in GAs biosynthetic pathway (Prot. 46) was also downregulated from 2 dpi onwards. GAs are formed from isoprenoid building blocks synthesized in the 2-C-methyl-d-erythritol-4-phosphate pathway [67] and we also detected that an enzyme of this pathway (Prot. 41) was downregulated in aphid-infested plants.

### Stress and defense

#### Antioxidant and detoxifying systems

ROS play an important role in signaling pathways that regulate acclimatory and defense responses in plants. The “oxidative burst”, resulting from the generation of ROS, is a rapid and common plant response to many abiotic and biotic stresses, including aphid herbivory [36, 38, 66, 68]. However, as the accumulation of ROS is generally harmful for cells, plants must find a balance between producing ROS for defense and producing ROS scavengers to help stabilize plant tissue [17]. Recent discoveries have pointed that ROS-scavenging enzymes and non-enzymatic antioxidants function not only to keep ROS homeostasis but are also involved in ROS-dependent signaling during plant acclimation responses to environmental stresses [68]. Thus, it is not strange to observe that aphid feeding can induce expression of certain antioxidant enzymes while suppressing others within a single plant species [17, 42, 69]. In the present study, we obtained a strong representation of oxidative stress-related proteins among the aphid-regulated proteins (Prot. 81–88). However, only two of them (Prot. 82 and 84) accumulated at 2 dpi and the content of the rest was either unaltered or reduced below control levels at longer times (4 and/or 7 dpi). The reduced synthesis of these enzymes has been suggested to be a plant defense strategy to increase ROS levels, which are directly toxic to insects [49]. However, given the late time of its deployment, downregulation of ROS-scavenging enzymes seems to be a trait more related with pepper susceptibility than with defense against aphids. Accordingly, resistance to aphid infestation in wheat was closely linked to its ability to control ROS levels through increased levels and activities of antioxidative enzymes [70, 71]. Another protein regulated in response to aphid infestation was lactoylgluthatione lyase (Prot. 89), which plays a critical detoxification role in cells by catalyzing the conversion of a variety of aromatic and aliphatic α-ketoaldehydes, such as methylglyoxal, to α-hydroxy thioesters [72]. Lactoylgluthatione lyase was upregulated at 2 dpi in aphid-infested plants but strongly decreased its content from 4 dpi onwards. Such a lowering effect could be explained as an attempt to maintain a toxic environment for aphids by the plant [48].

#### Chaperones

Molecular chaperones are key components contributing to cellular homeostasis being responsible for protein folding, assembly, translocation, and degradation in several normal cellular processes. As a large array of stresses cause protein dysfunction, chaperones play a critical role in cell survival by assisting protein refolding and preventing the aggregation of non-native proteins [73]. In the present study, a wide range of proteins with chaperone activity (Prot. 66–70, 72, 90–92) were regulated in response to aphid infestation. These proteins were mostly upregulated at short times of infestation (2 dpi), but downregulated or unchanged at 7 dpi. The fact that these proteins are not upregulated at long time of infestation may be part of the aphid manipulation of plant metabolism to promote the release of amino acids from proteins in order to improve its feeding [48].

#### Direct and indirect defenses

A hydroperoxide lyase (HPL, Prot. 94) was strongly upregulated in aphid-infested plants from 2 dpi to 7 dpi, which may play an important role in pepper defenses. HPL cleaves 13-hydroperoxides generated by LOX to produce green leaf volatiles (GLVs) and traumatin (12-oxododecenoic acid), the wound hormone involved in healing of damaged tissues [74]. In transgenic potato plants, HPL depletion highly reduced levels of the GLVs hexanal and 3-hexenal and correlated to an increase in aphid performance [75]. Conversely, we detected two defence-related proteins that were upregulated or unchanged at 2 dpi but strongly downregulated at longer times of infestation (4 and 7 dpi), a kirola protein (Prot. 93) and a CBS domain containing protein (CDCP, Prot. 95). Kirole has the highest sequence identity with members of the major latex/ripening-related (MLP/RRP) family [76] and its fold is very similar to that of pathogenesis-related (PR)-10 proteins [77]. Interestingly, a kirola-like protein was also upregulated in pepper plants in response to the whitefly *Bemisia tabaci* [78] and in *Nicotiana* sp. plants infected by *B. tabaci* [79] and virus [78, 80]. On the other hand, CDCPs may have a key role in stress response/tolerance and development in plants [81–83]. Specifically, a gene encoding a CDCP (OsBi1) is implicated in the resistance of rice plants to the sucking herbivore brown planthopper (*Nilaparvata lugens* Stål.) [84]. Our results are in good agreement with other studies showing a downregulation of some proteins with a direct role in plant defense in response to aphid infestation, such as a receptor-like protein kinase and an hydroxyproline-rich glycoprotein in *Arabidopsis* [44] or the insect-specific defense protein Hfr-2 in wheat [42].

### Other processes

#### Signaling networks

In plants, Ca^2+^ acts as intracellular second messenger being especially important for the maintenance of cellular homeostasis and signal transduction pathways through its binding to various Ca^2+^ sensor proteins. These include calmodulins, calmodulin-binding proteins, calcium-dependent protein kinases (CDPKs) and other Ca^2+^-binding proteins [12, 36]. In the present study two Ca^2+^-binding proteins were regulated in response to aphid infestation. Calnexin homolog 1 (Prot. 66), was strongly upregulated at 2 dpi and 4 dpi recovering control levels at 7 dpi, whilst calmodulin-7-like (Prot. 130) was upregulated at 2 dpi but strongly decreased its content at 4 and 7 dpi, which may be interpreted as an aphid-influenced plant response [7, 49]. Although we did not detect any CDPK regulated by aphid infestation, a protein similar to 14–3-3 protein 6 (Prot. 131) was upregulated at 2 dpi. Recent advances have demonstrated complex regulatory network between CDPKs and 14–3-3 proteins and that they act in concert in plant signaling pathways [85]. 14–3-3 proteins bind to target proteins and modulate their activity, stability, subcellular localization, or participation in a protein complex, playing key roles in many physiological processes in plants, including cell growth and division, primary metabolism, response to light, abiotic and biotic stress responses and participate in processes mediated by almost any phytohormone [85–87]. Interestingly, 14–3-3 protein 6 was strongly downregulated at 4 and 7 dpi, as noticed for calmodulin-7. Finally, it is worth mentioning that remorin (Prot. 114) was also downregulated in aphid-infested plants from 2 to 7 dpi. Remorin proteins may act as molecular scaffolds regulating signal transduction and has been proposed to be involved in plant-microbe cell signaling [88].

#### Transport

The defensive responses to aphid attack are accompanied by the activation of sequences involved in cellular transport and exocytosis [16], given that many of the defense-related proteins produced are synthesized and then secreted to their various destinations within the cell by the Golgi apparatus [20]. Accumulating evidence also points to a more direct role of the secretory pathway in plant defense responses. In *Arabidopsis*, the induction of the protein secretory pathway is required for systemic acquired resistance [89]. Moreover, a recent study in the *Arabidopsis* - *M. persicae* system has shown that the aphid effector Mp1 associates with host vacuolar protein sorting associated protein 52 (VPS52), a trafficking pathway protein, to promote infestation [90]. In the present study, coatomer subunit gamma (Prot. 100), involved in vesicle-mediated transport and intracellular protein transport, was strongly upregulated in aphid-infested plants at 2 and 4 dpi. Moreover, signal recognition particle 43 kDa protein (Prot. 101) and protein TIC110 (Prot. 102), both implicated in protein import into chloroplasts, resulted upregulated or unchanged at 2 dpi but downregulated at later times of aphid infestation (4 and 7 dpi), whilst patellin 3-like protein – which binds phospholipids and function in diverse signaling pathways in plants [91]- was upregulated at 7 dpi.

#### Growth and development

Plant fitness is optimized when growth and defense are appropriately prioritized in response to both environmental and developmental cues [92, 93]. In the present study, an early nodulin-like protein (ENODL; Prot 101) was upregulated at 2dpi. Nodulin-like proteins regulate plant growth and development being involved in transport of different nutrients, amino acids, hormones and solutes [94]. Interestingly, an allelic variation in an ENODL gene influences insect community species diversity and the abundance of interacting foundation species: aphids and tending ants [95]. Also, three ENODL proteins putatively increased Bt rice resistance to brown planthopper infestation [96]. This suggests that ENODL proteins may influence different plant–insect interactions.

## Conclusions

LC-MS/MS analysis coupled with bioinformatics resulted to be a powerful approach to study pepper leaf proteome responses to *M. persicae* infestation. The fact that an unexpectedly very low number (6) of proteins were found to be regulated in the experiment with leaf discs, even though the response was studied at local level, leads us to hypothesize that aphids are either preventing the activation of plant defense responses or remain undetected by the plant, at least in the particular plant-aphid system studied in this work. Conversely, a systemically high density of aphid infestation in pepper plants resulted in a set of 140 proteins differentially regulated in leaves as a consequence of the presence of aphids. These proteins belong to nearly all routes of plant primary metabolism, including photosynthesis, photorespiration, amino acid and carbohydrate metabolism, translation, protein folding and degradation, and energy production and suggest a large metabolic reprogramming occurring in aphid-infested pepper leaves. Photosynthesis has been so far the metabolic process in which a higher number of proteins have been regulated by the presence of aphids. In fact, a large amount (48%) of the regulated proteins were chloroplastic, which highlights the relevance of this organelle in the plant response to aphids. Other processes such as amino acid and carbohydrate metabolism or protein folding and degradation have been likewise highly affected by the presence of aphids. Considering the magnitude of the change in the regulated proteins and its behaviour with time, we can also conclude that at short times of infestation (2 days), most of the changing proteins were upregulated. At 4 dpi the proportion among up- and downregulated proteins was almost equilibrated while at longer times (7 dpi), most of proteins were downregulated. It is worth noting that proteins directly involved in defense were scarce and mostly downregulated in response to aphid infestation, just as proteins involved in hormone signalling pathways. This slight defensive response elicited in pepper plants may be ascribed to the susceptibility of this species to aphids. Collectively considered, the results outline a significant metabolic drift in the pepper plant in favour of the feeding requirements of the aphids. Whether this metabolic drift is directed by elicitors derived from the aphid is a matter for further research. Furthermore, the leaf proteomic information obtained in the present study will help to the understanding of the defense response of an important agricultural crop to aphids.

## Methods

### Plant material

Pepper plants (*C. annuum* var. California Wonder seeds from Ramiro Arnedo S. A, Murcia, Spain) were germinated in plastic pots with a 1:1 mixture of peat (Prohumin potting soil, Projar S.A., Valencia, Spain) and vermiculite. Plants were maintained in a growth chamber at 24 °C and 70% relative humidity under a 16:8 h photoperiod (day/night). Plants were watered three times a week.

### Aphid infestation

A *M. persicae* colony was maintained on pepper plants as described previously [50]. Two independent assays have been performed: one with a low density of aphids (20 per plant) confined to a single leaf with the aid of a clip cage (see below for description). In the second experiment, a high aphid density (200 per plant) was used without restrictions of movement. In both cases, experiment started 5 weeks after sowing and pepper plants were infested with wingless adult aphids.

#### Aphids confined to single leaves by clip cages

Twenty aphids were placed on the abaxial surface of a single leaf at the second true leaf pair of leaves and confined into a clip cage (BioQuip Products, Inc. USA), thus preventing them from freely moving throughout the plant. The infestations with aphids were initiated in a staggered manner [97], so that all tissue samples were harvested at the same time, to avoid any bias derived from diurnal cycling and/or changes in environmental conditions. Samples were collected at 3 h post-infestation (hpi), 2 days post-infestation (dpi) and 4 dpi. Appropriate controls consisting on uninfested plants that received empty clip cages for the same time periods as the aphid-infested plants were also included. At the end of the experiment, the leaf area under the cages was cut and aphids brushed off. The resulting leaf discs were then flash frozen in liquid nitrogen and stored at − 80 °C prior to freeze-drying. Tissue was finally grounded and stored at 4 °C into airtight vials until extraction. Four biological replicates were included at each time point, each one consisting on pooled samples from two plants.

#### High density of aphid infestation without movement restrictions

Plants were infested with 200 aphids distributed evenly throughout the plant without any additional restriction for their movement. This high density of aphids was used to ensure the infestation of all plant leaves. Infested plants were enclosed into nets to avoid aphid transference among treatments. Plants destined to be controls of the experiment were also enclosed into a net for the same time periods but without aphids. As above, plants were infested sequentially and leaf tissue was harvested at 2 dpi, 4 dpi and 7 dpi. At the end of the experiment aphids were brushed off and all the fully-expanded leaves of the plants were collected. All plant material was collected at the same time, flash frozen in liquid nitrogen, freeze-dried and grounded. Four biological replicates were included at each time point, each one consisting on pooled samples from two plants.

### Protein extraction and quantification

Total protein extracts were prepared according to the TCA (trichloroacetic acid)–acetone–phenol method [98]. Dried pellet was dissolved in 6 M urea and the protein concentration was assayed using an RC/DC assay (BioRad, USA). Bovine serum albumin (BSA) was used to create a standard curve.

### Tryptic in-solution digestion

Fifty micrograms of protein sample were reduced with 5 μL of 0.2 M dithiothreitol followed by incubation for 1 h at 37 °C and S-alkylation with 20 μL of 0.2 M iodoacetamide followed by incubation for 1 h in the dark at room temperature. Then, 25 mM ammonium bicarbonate buffer was added to reduce the concentration of urea to 0.6 M. For in-solution digestion, trypsin was added to the protein mixture (enzyme:substrate ratio of 1:30 w/w) and incubated at 37 °C for 16 h. In order to ensure a complete digestion, additional trypsin (1:60, w/w) was added to the sample and it was incubated for 5 h more. Then tryptic peptides were dried down using a Speed-Vacbenchtop centrifuge and later resuspended in 5% acetonitrile and 0.5% trifluoroacetic acid. The resulting peptides were desalted, in batches of 30 μg of protein, through PepClean C-18 Spin Columns according to manufacturer recommendations (Agilent Technologies). Finally, eluted peptides were dried down and resuspended in 10 μL of first LC mobile phase (5% acetonitrile and 0.1% formic acid).

### LC-MS/MS analysis

To perform label-free relative quantitation, the samples of treated and untreated groups were analyzed over four technical replicates. Peptide separation was performed using an Agilent 1290 Infinity LC system coupled to the 6550 Accurate-Mass QTOF (Agilent Technologies, Santa Clara, CA, USA) with electrospray interface (Jet Stream Technology) operating in positive-ion mode (3500 V) and in high sensitivity mode. The best conditions for the electrospray interface were: gas temperature 250 °C, drying gas 14 L/min, nebulizer 35 psi, sheath gas temperature 250 °C, sheath gas flow 11 L/min. Samples were injected (10 μL) on an Agilent Advance Bio Peptide mapping column (2.1 × 250 mm, 2.7 μm) (Agilent Technologies) with a 3–40% gradient of solvent B (0.1% formic acid in 90% acetonitrile) for 140 min operating at 50 °C and a flow rate of 0.4 mL/min. Agilent Mass Hunter Workstation Software was employed for the data acquisition. LC/MS Data Acquisition B.08.00 (Build 8.00.8058.0) was operated in Auto MS/MS and the 20 most intense ions (charge states, 2–5) within 300–1700 m/z mass range (over a threshold of 1000 counts) were selected for MS/MS analysis. The quadrupole was set to “narrow” resolution and MS/MS spectra (50–1700 m/z) were acquired until 25,000 total counts or for a maximum accumulation time of 333 ms. To assure the desired mass accuracy of recorded ions, continuous internal calibration was performed during analyses with the use of signals m/z 322.0481 (detected m/z [C6H18N3O6P3 − H]^+^) and m/z 1221.9906 (detected m/z [C24H18O6N3P3F36 -H]^+^).

### LC-MS/MS data analysis

#### Protein identification

The Extraction tool of Spectrum Mill Proteomics Workbench Rev. B.04.01.141 (Agilent Technologies) was used to process MS/MS spectra data and determine monoisotopic masses and charge states, to merge MS/MS spectra with the same precursor (Δm/z < 1.4 Da and chromatographic Δt < 60 s) and to select high quality spectra. The reduced data set was searched against the *Capsicum annuum* NCBInr database in the identity mode using the MS/MS Search tool with the following settings: trypsin, up to 2 missed cleavages, carbamidomethylation of cysteines as fixed modifications, oxidation of methionine as variable, mass tolerance of ±20 ppm for precursor and ± 50 ppm for product ions. The precursor mass shift was set between − 18 Da to 177 Da to take into consideration variable modifications such as the presence of sodium and potassium adducts. Peptide hits were validated in the peptide mode to achieve a false discovery rate (FDR) of < 1.2% and then in the protein mode according to the score settings recommended by the manufacturer. Positive identifications were considered only when two or more peptides were matched, and their summed score was > 20.

#### Statistical analysis

For label-free relative quantitation, differentially expressed proteins were assessed based on the regulation of the peptides. Missing values imputation of protein intensities were performed from a normal distribution (width: 0.3, down shift: 1.8) using Perseus software [99]. Protein lists were exported to the Mass Profiler Professional (MPP) software v. 14.9.1 (Agilent Technologies) for statistical data analysis. Data analysis was carried out based on the total spectra intensity of the proteins which were considered as entities in MPP. The entities were filtered based on their frequency, selecting those consistently present in all replicates of at least one treatment.

In the case of the experiment with clip cages, comparison of protein expression levels from various samples involved moderated T-tests (*p* ≤ 0.05) between control and aphid-infested leaves, with each time point (3 hpi, 2 dpi and 4 dpi) being analyzed separately. On the other hand, in the case of the experiment with a high density of aphids freely moving by the plant, an ANOVA followed by Tukey HSD posthoc test (p ≤ 0.05) was performed to compare protein expression levels between the different treatments (control vs 2 dpi vs 4 dpi vs 7 dpi). In all the cases, Benjamini-Hochberg procedure was employed to overcome the problem of multiple test analysis (false discovery). Proteins with ≥2.0 fold change respect the controls, positive or negative, were defined as upregulated or downregulated, respectively.

#### Functional classification

The Blast2GO v2.4.0 (BioBam, Valencia, Spain) application was used to automatically assign protein description and take-up annotations from homologous sequences of public databases, following the indications detailed in Martínez-Esteso et al. [100]. Additionally, annotation was augmented by the Annotation Expander (ANNEX), which uses an additional Gene Ontology structure to suggest new biological process and cellular component annotations. Annotations for biological process (P), molecular function (F) and cellular component (C) performed by Blast2GO were write down and manually revised to guarantee accurate assignment. Then, proteins were manually assigned to one of the following 14 functional categories: photosynthesis, amino acid metabolism, carbohydrate metabolism, lipid metabolism, protein folding and degradation, transcription-related, translation-related, energy production, stress and defense, cell organization, transport-related, hormone metabolism, miscellaneous and unknown function.

## Supplementary Information

**Additional file 1.** Complete list of the regulated proteins with the parameters for protein identification, protein abundances and their functional annotations.

**Additional file 2.** Some characteristic spectra of regulated proteins.

**Additional file 3.** List of regulated proteins (from top to bottom) represented in the heat map corresponding to Fig. 1.

## Data Availability

The mass spectrometry proteomics data have been deposited to the ProteomeXchange Consortium via the PRIDE [101] partner repository with the dataset identifier PXD022459. https://www.ebi.ac.uk/pride/archive/projects/PXD022459.

## Abbreviations

ABA: Abscisic acid
Ca^2+^: Calcium
CDCP: CBS domain-containing protein
CDPKs: Calcium-dependent protein kinases
dpi: Days post-infestation
hpi: Hours post-infestation
ENODL: Early nodulin-like
ET: Ethylene
GAs: Gibberellins
GLVs: Green leaf volatiles
GOGAT: Glutamine oxoglutarate aminotransferase
GS: Glutamine synthetase
HPL: Hydroperoxide lyase
HSPs: Heat-shock proteins
JA: Jasmonic acid
LOX: Lipoxygenase
MLP/RRP protein: Major latex/ripening-related protein
MeJA: Methyl jasmonate
PAIRBP1: Plasminogen activator inhibitor 1 RNA-binding protein
PAMP: Pathogen-associated molecular pattern
PR protein: Pathogenesis-related protein
ROS: Reactive oxygen species
rubisco: Ribulose-1,5-bisphosphate carboxylase/ oxigenase
SA: Salicylic acid
TCA: Tricarboxylic acid
