# Supplementary figures and images for: Proteome changes in pepper (Capsicum annuum L.) leaves induced by the green peach aphid (Myzus persicae Sulzer)
Source: BMC Plant Biol. 2021 Jan 6;21:12. doi: 10.1186/s12870-020-02749-x (PMC7788789; doi:10.1186/s12870-020-02749-x)

ABM55742.1

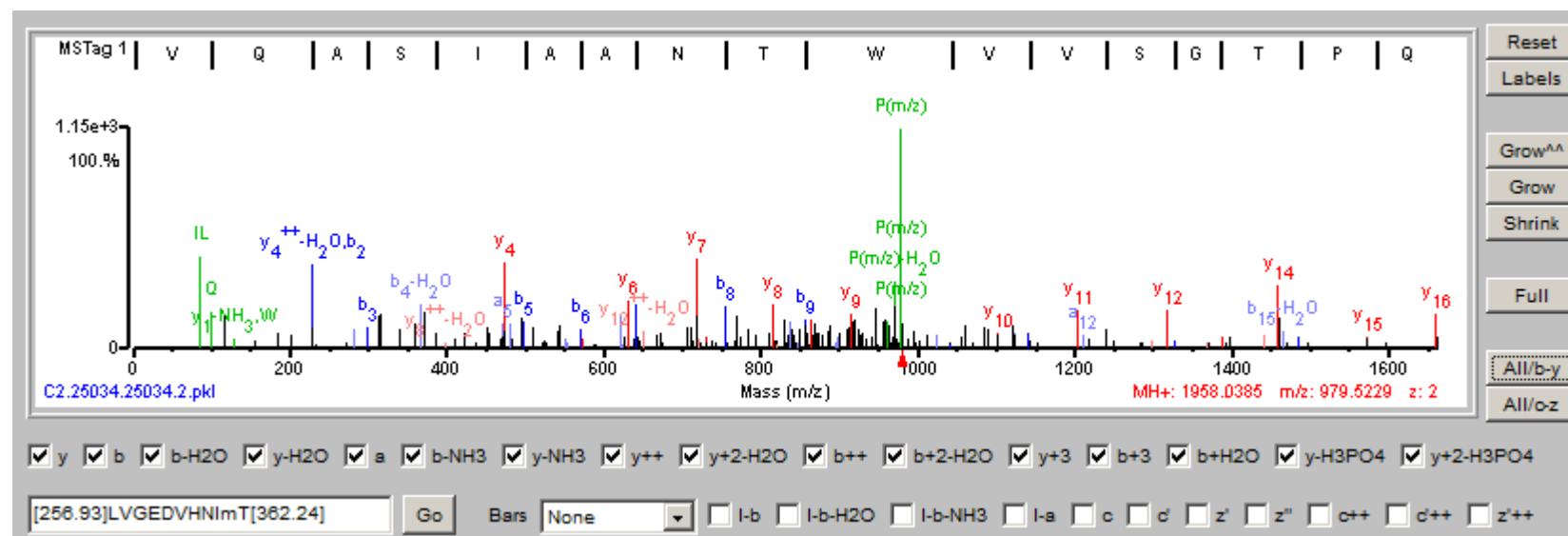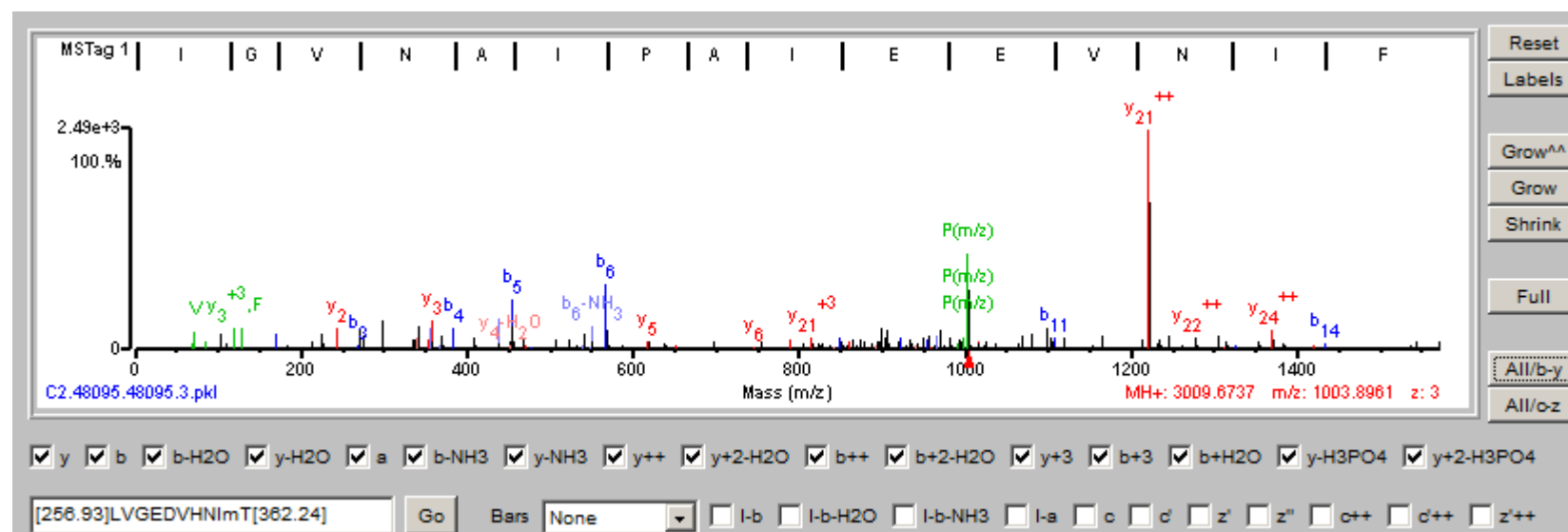

ADZ73653.1

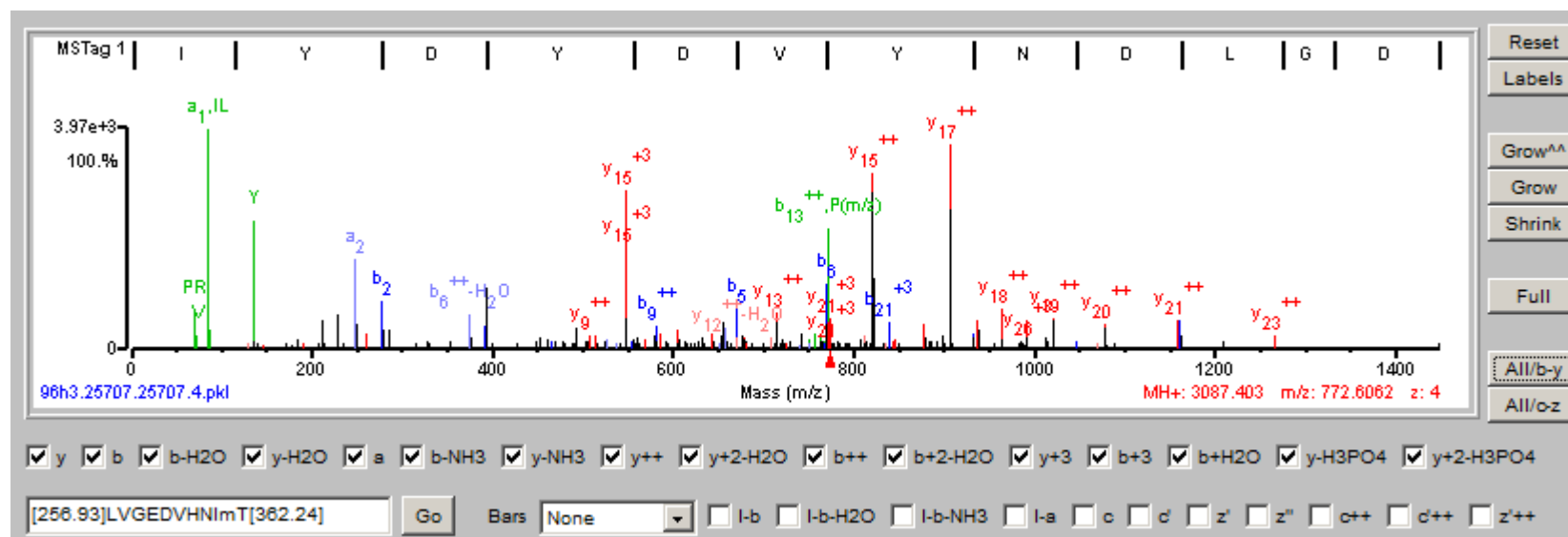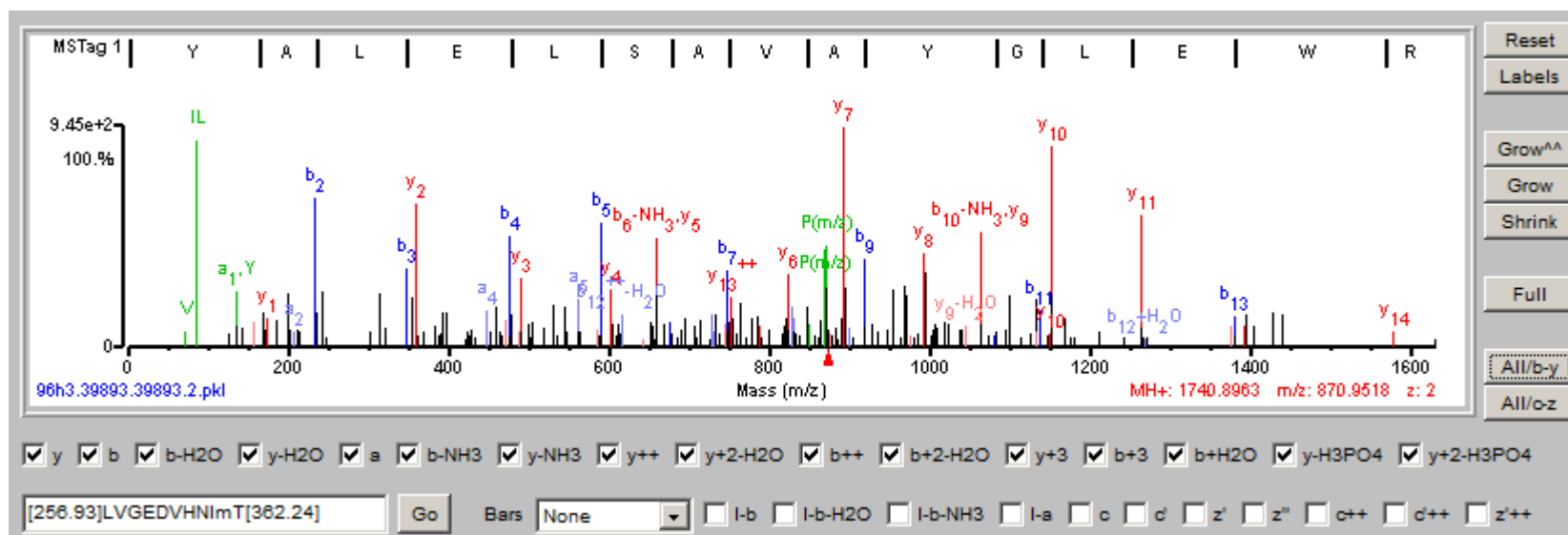

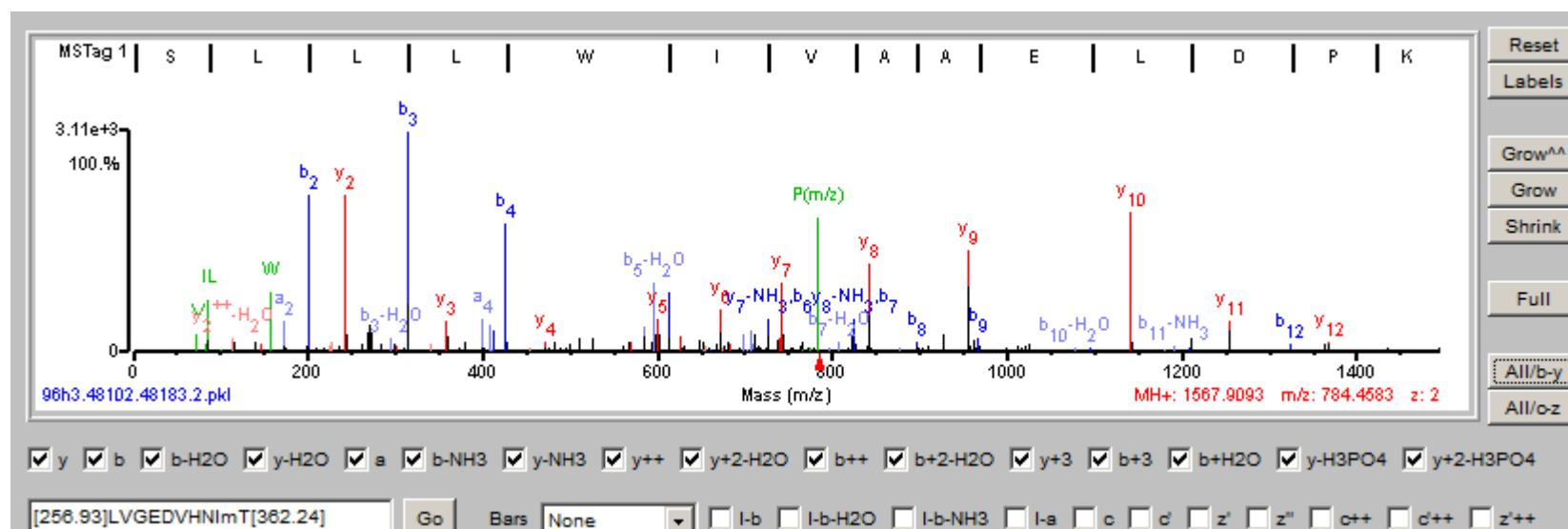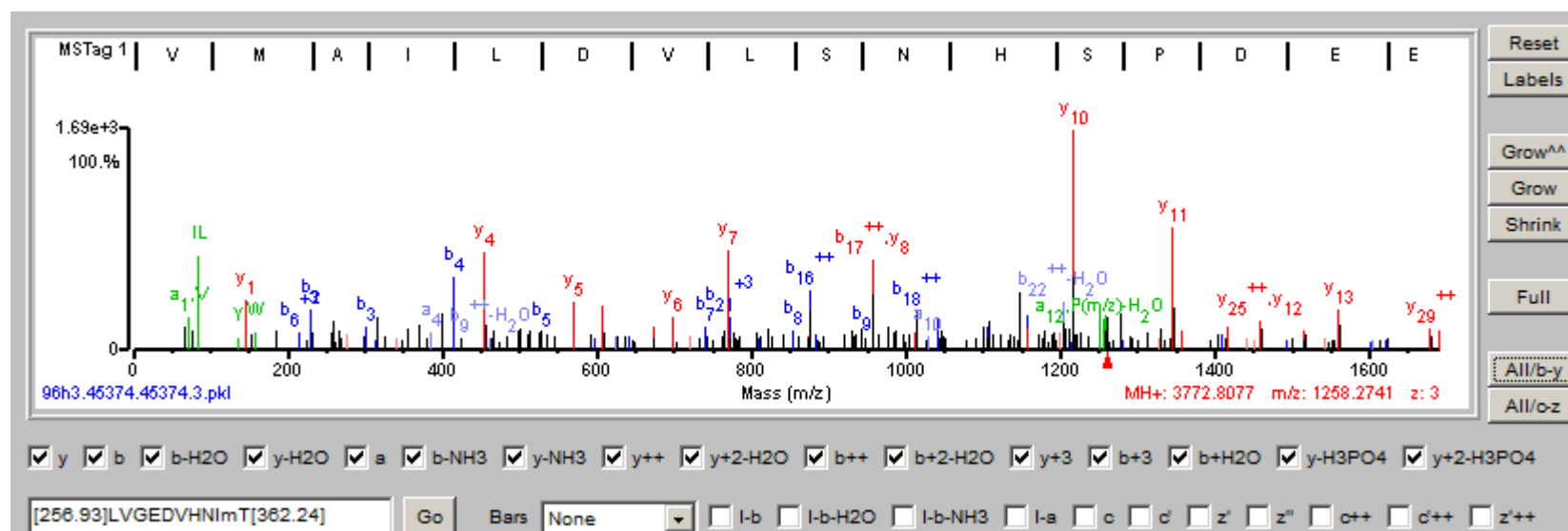

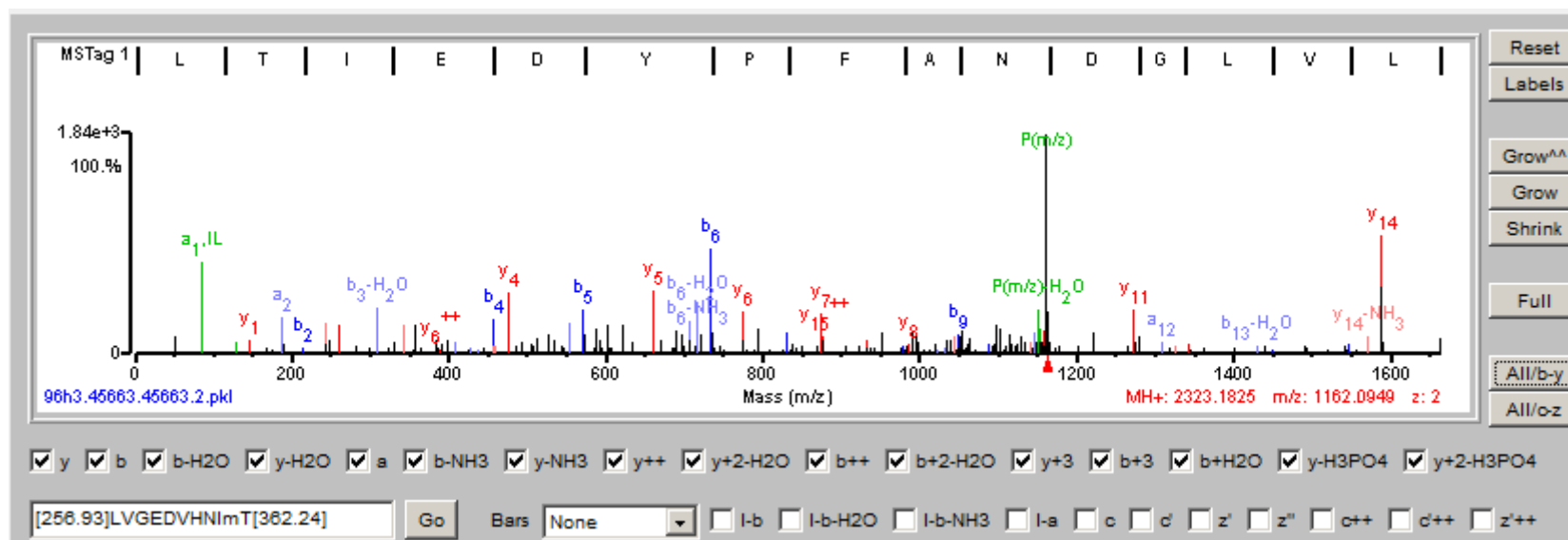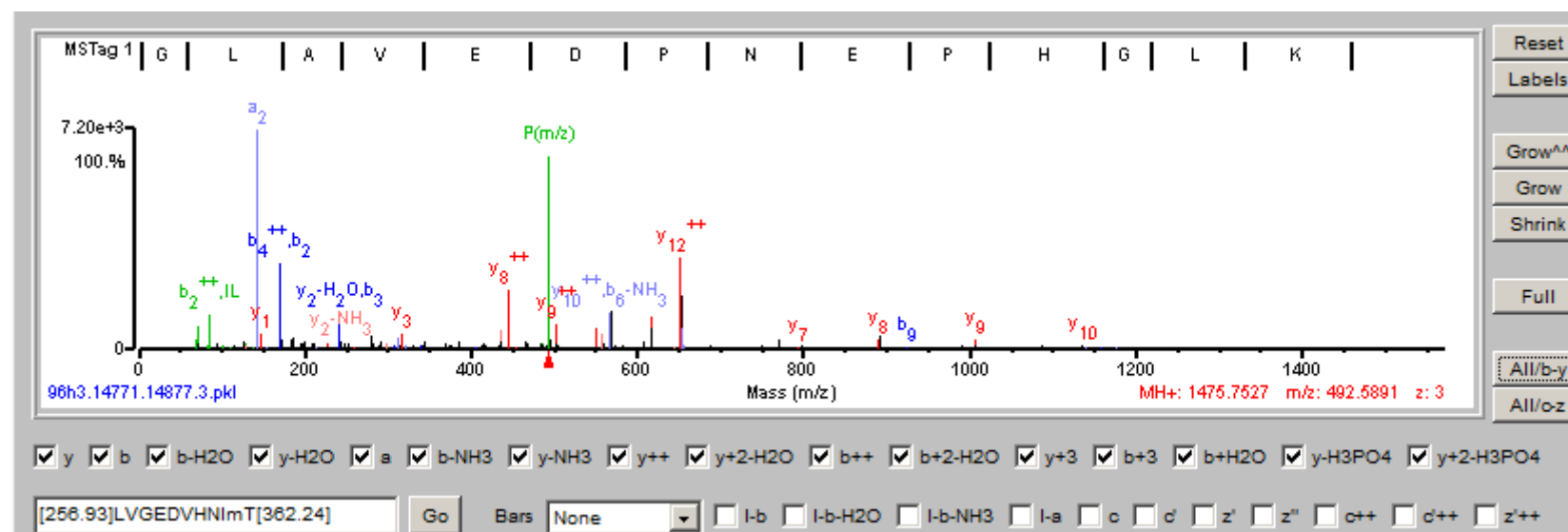

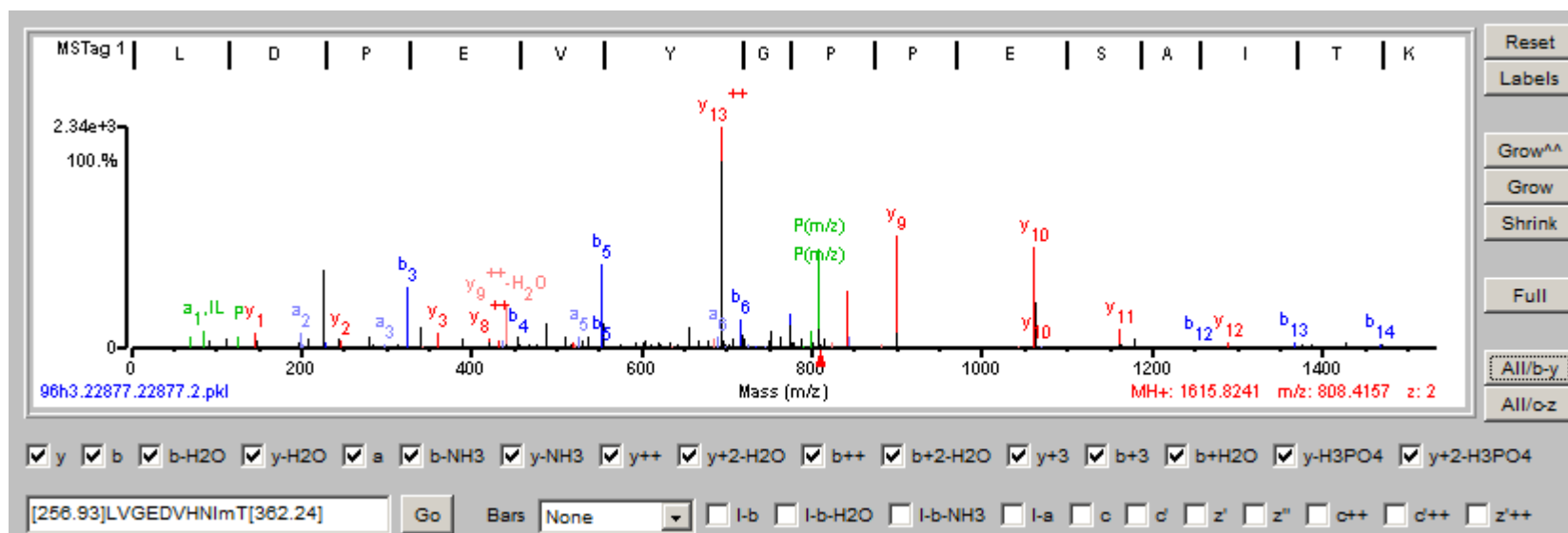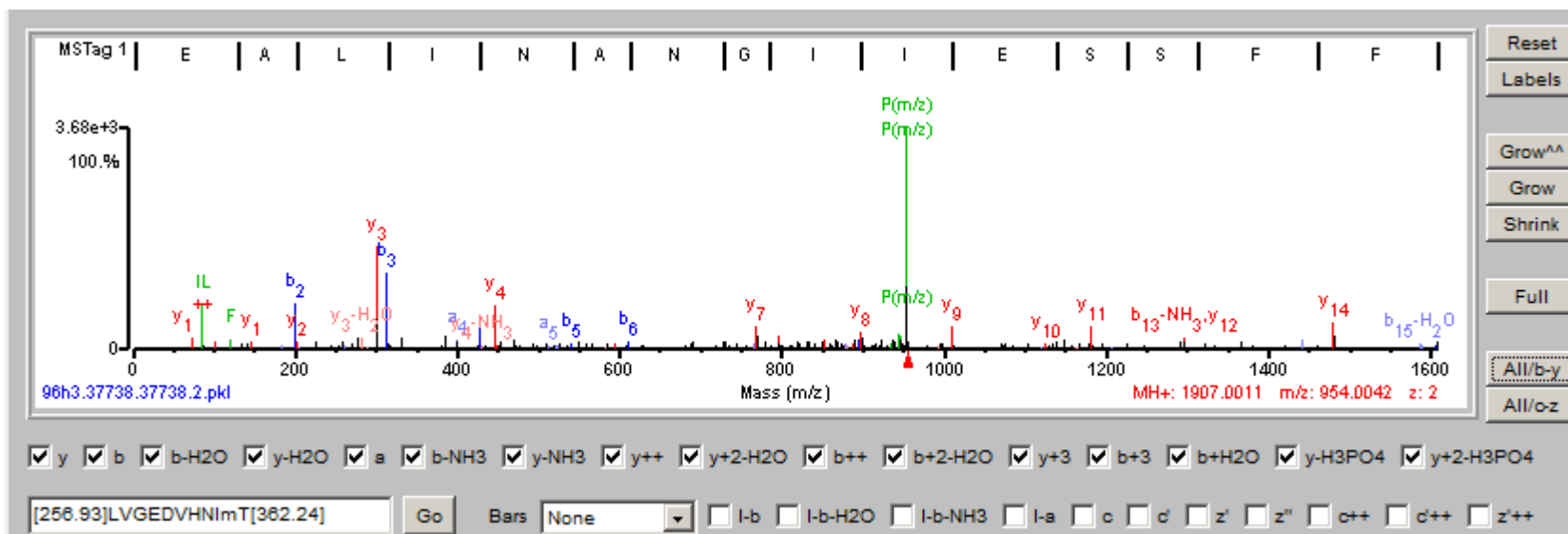

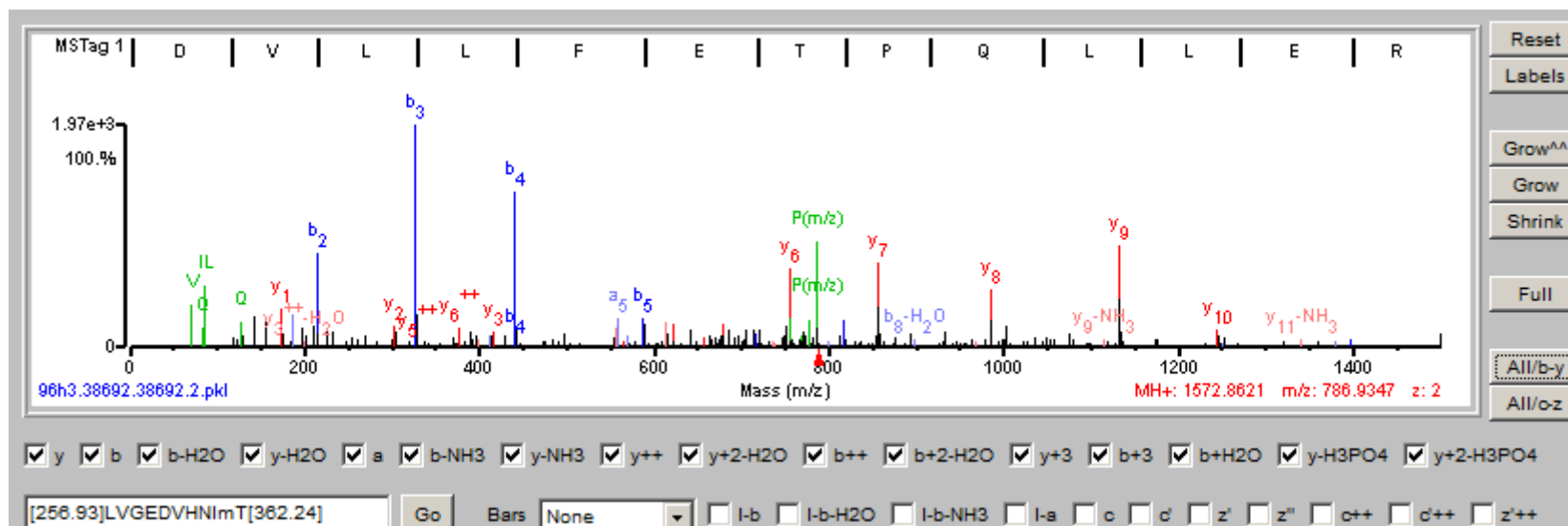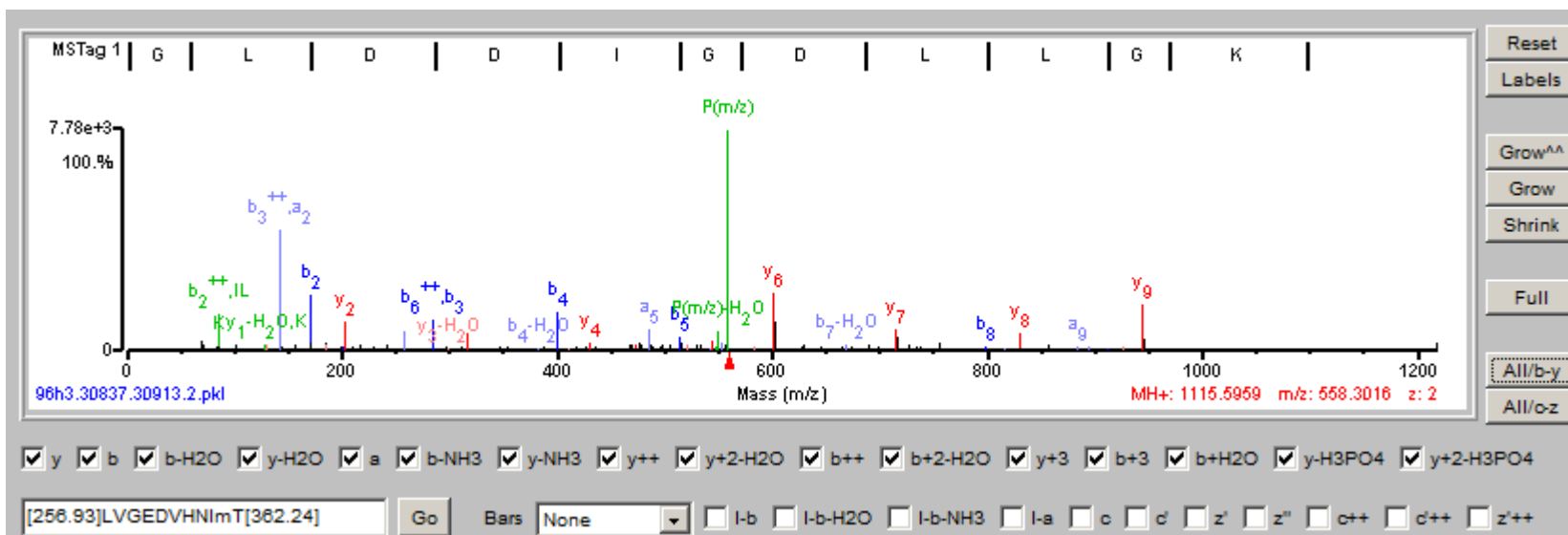

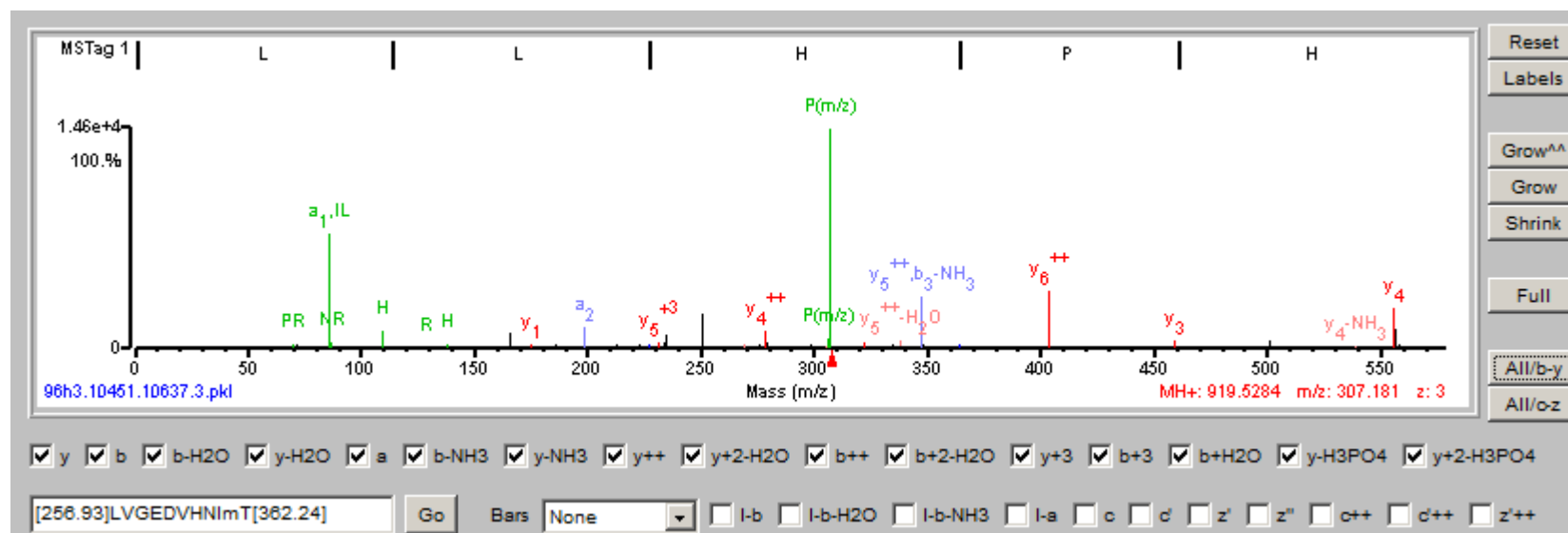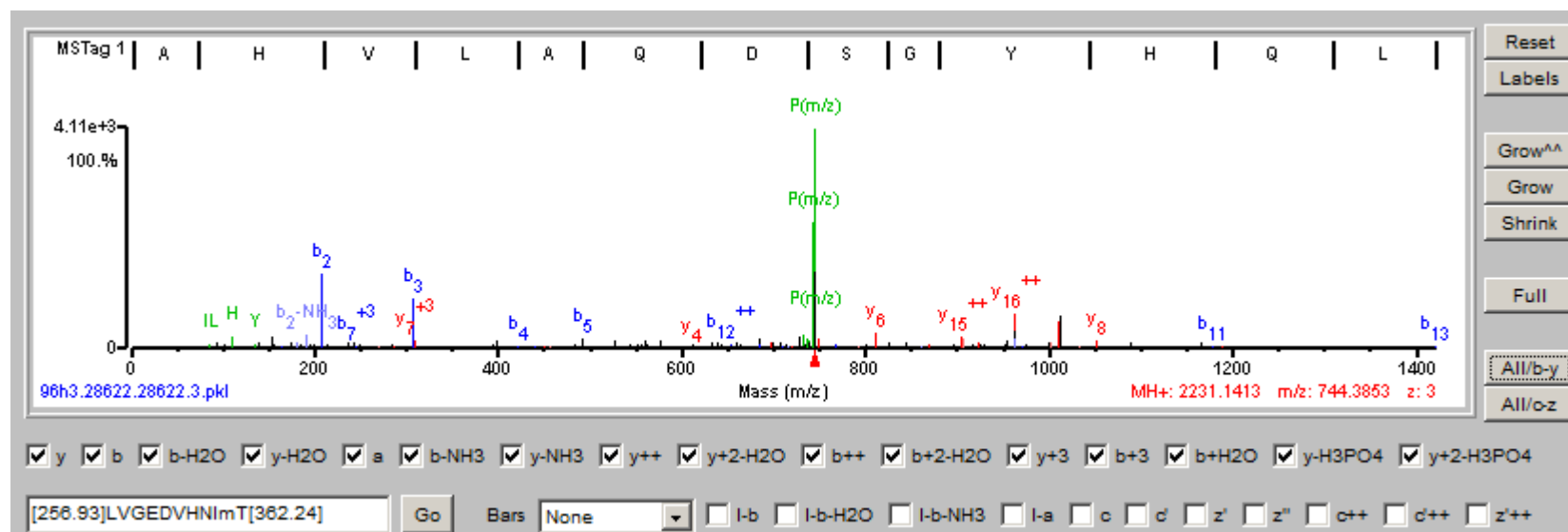

AFP90775.1

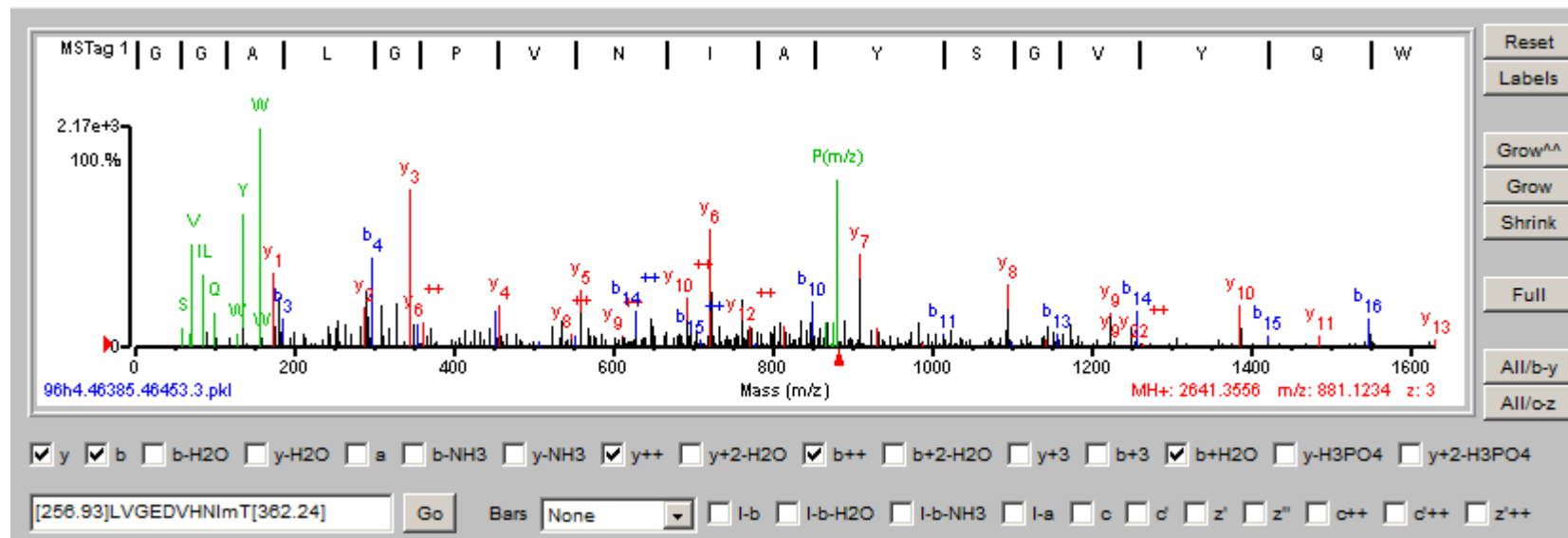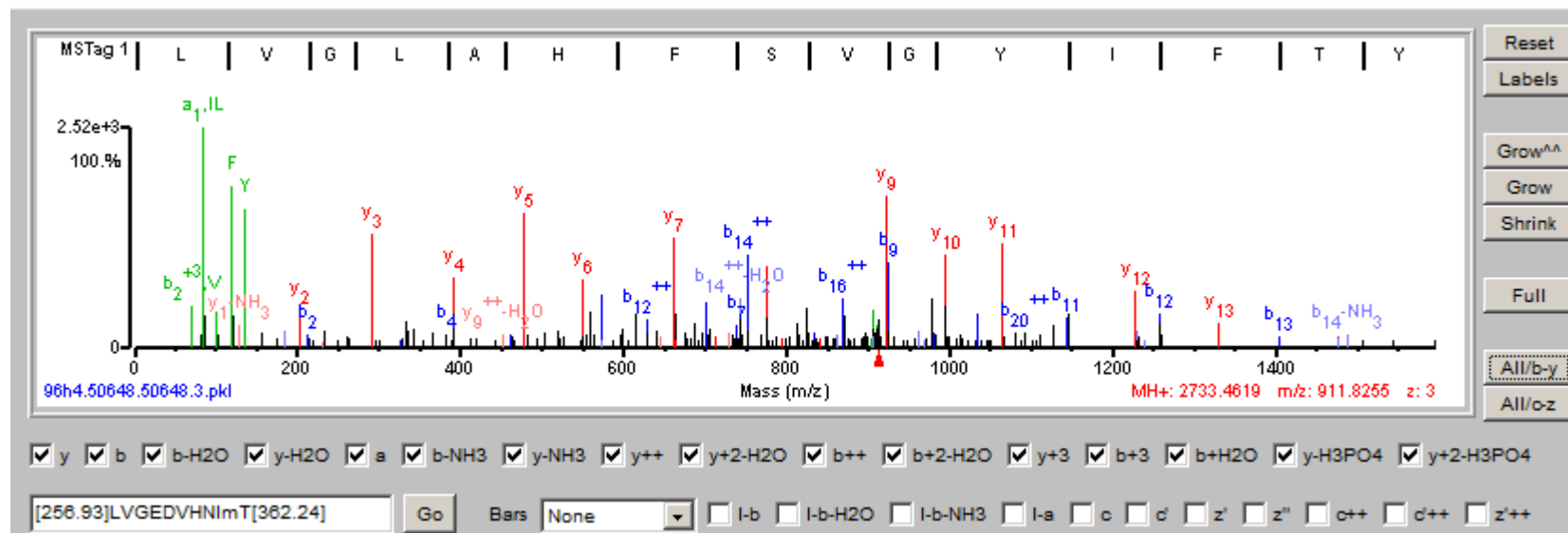

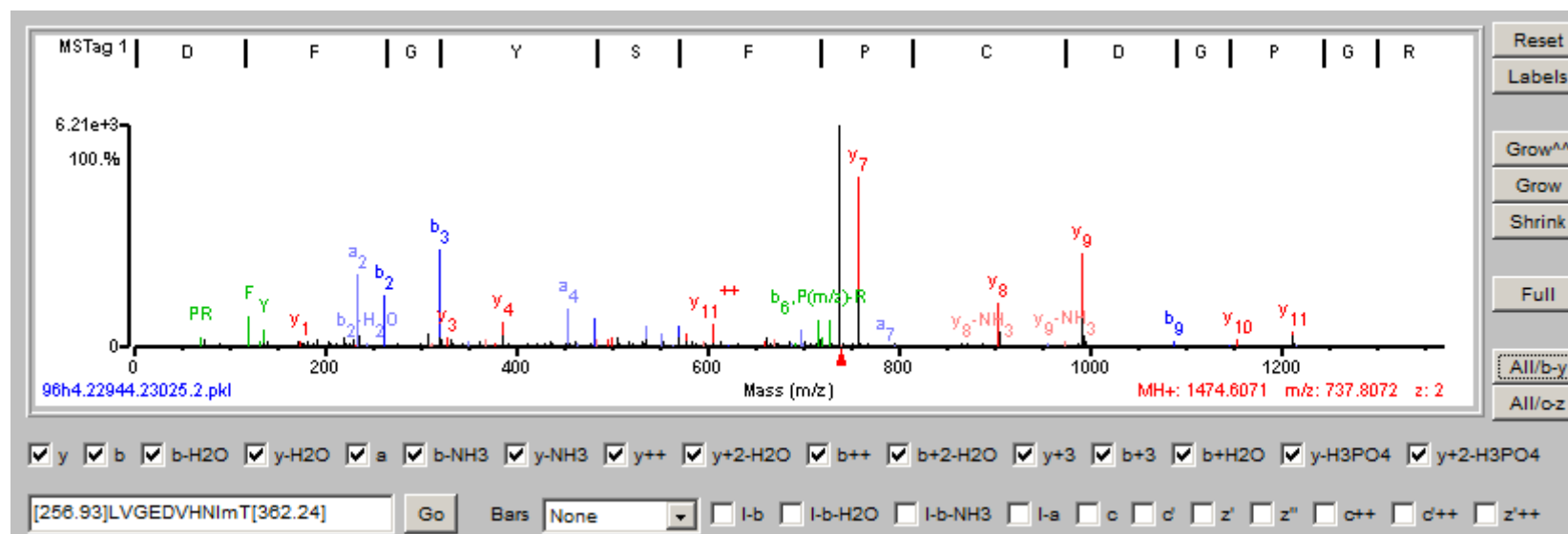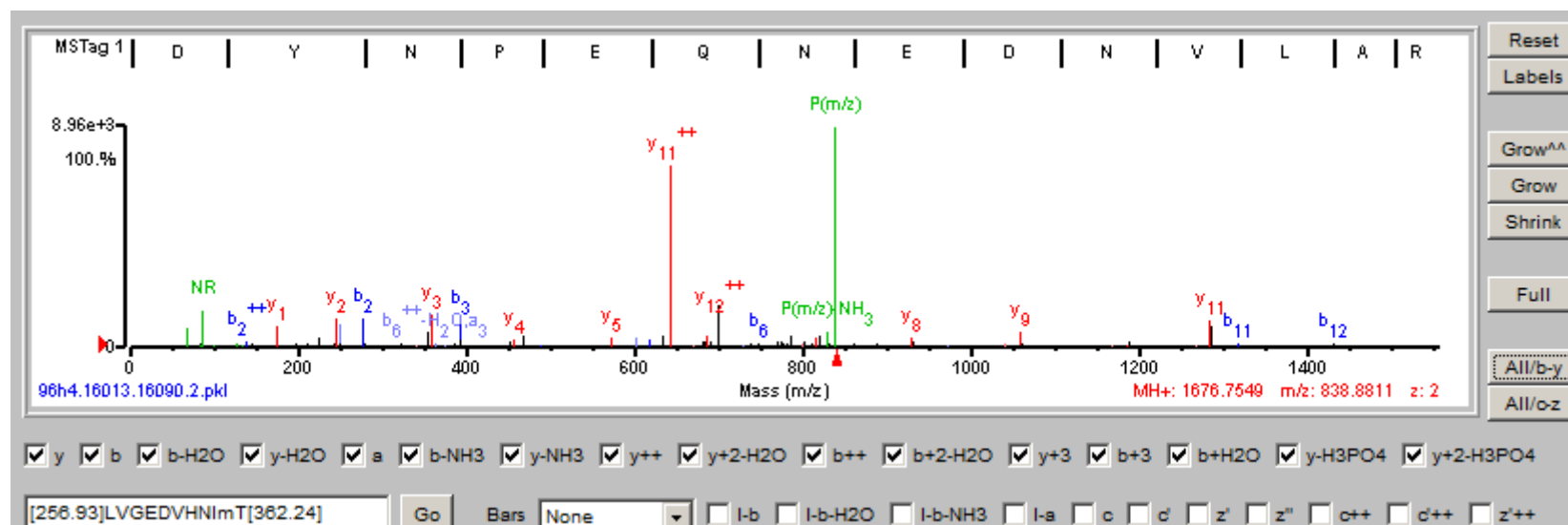

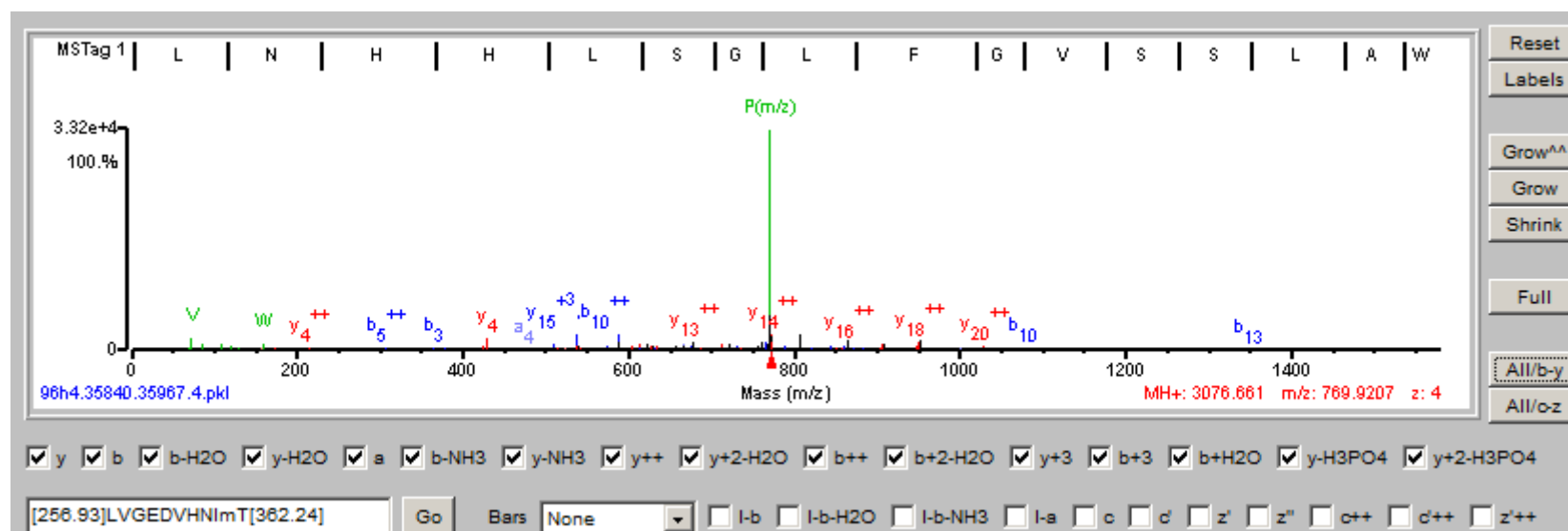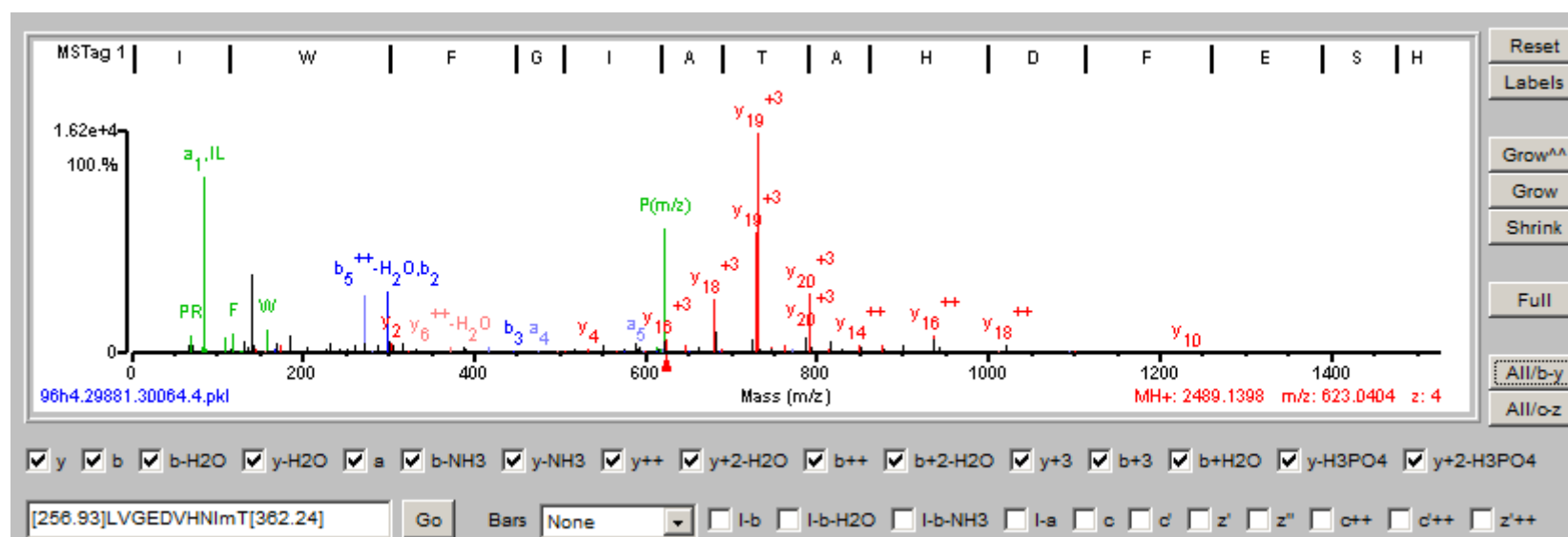

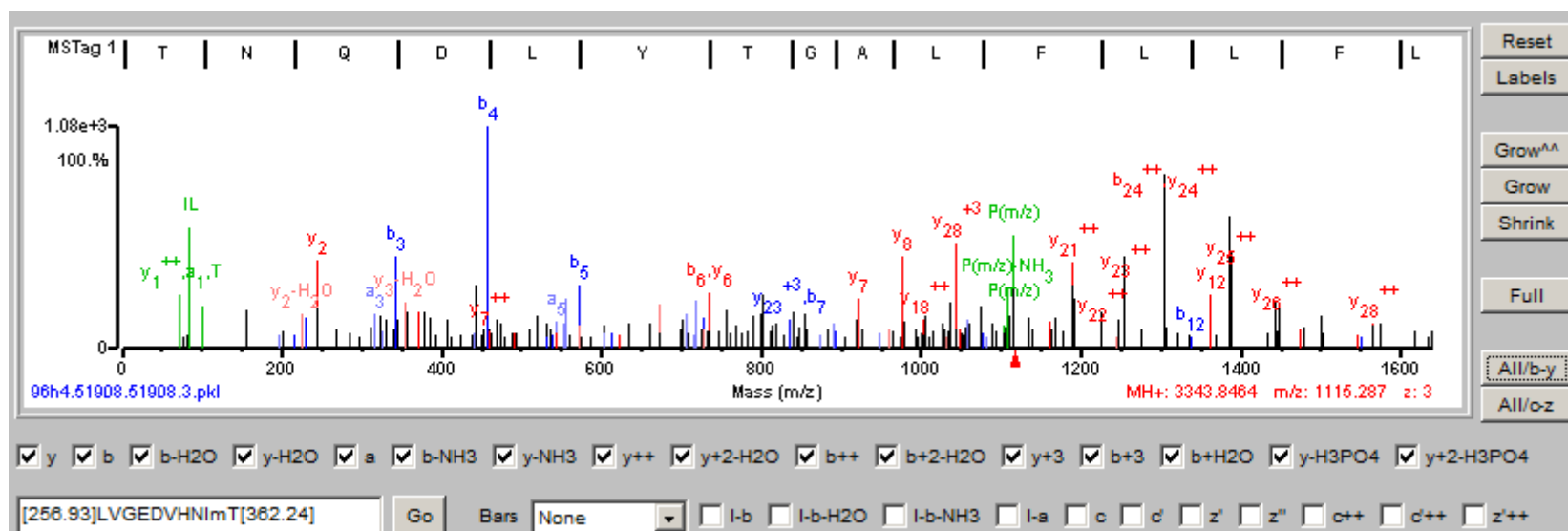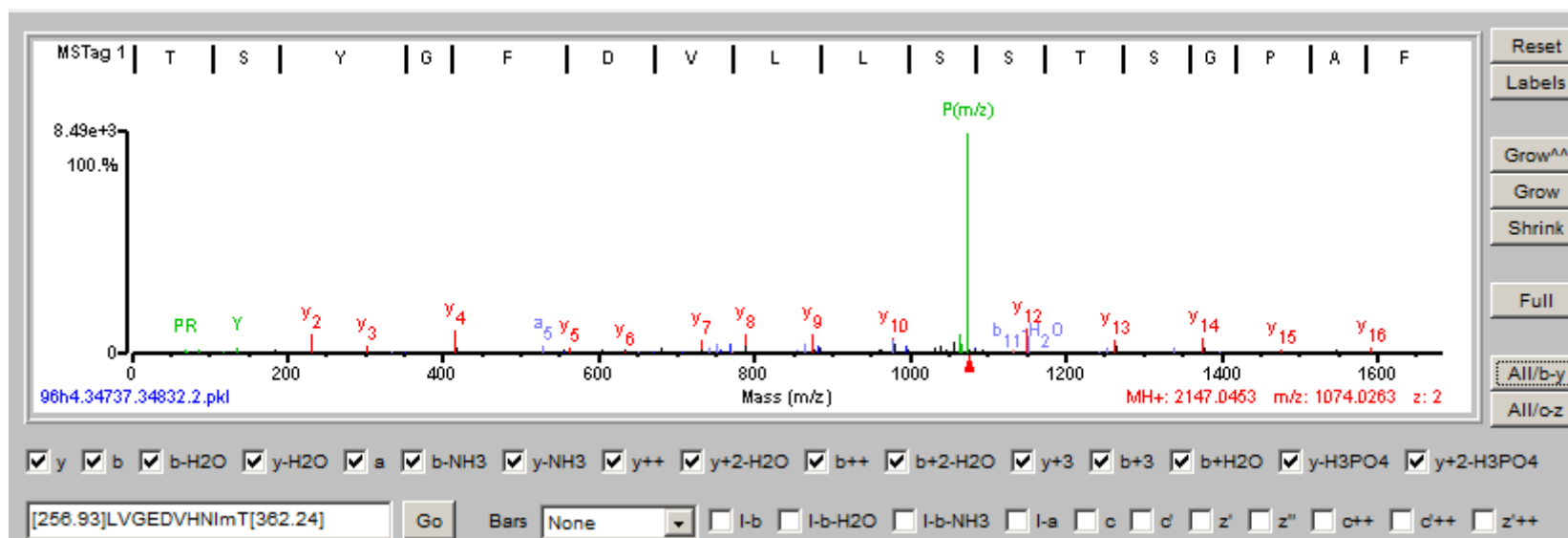

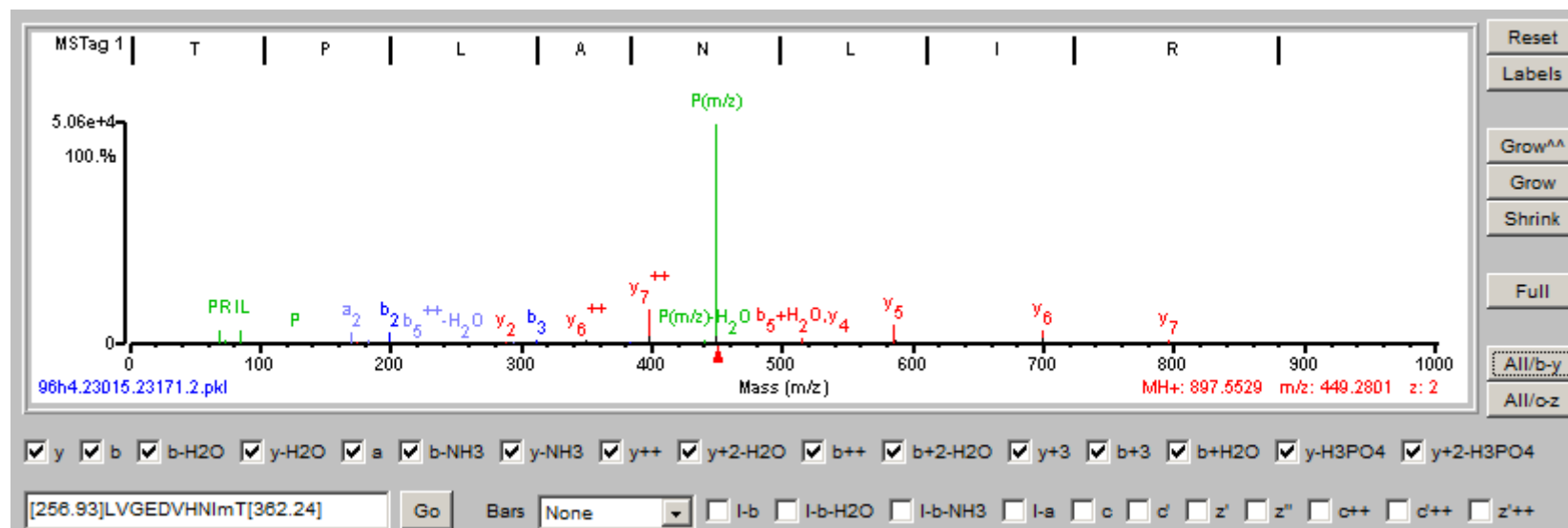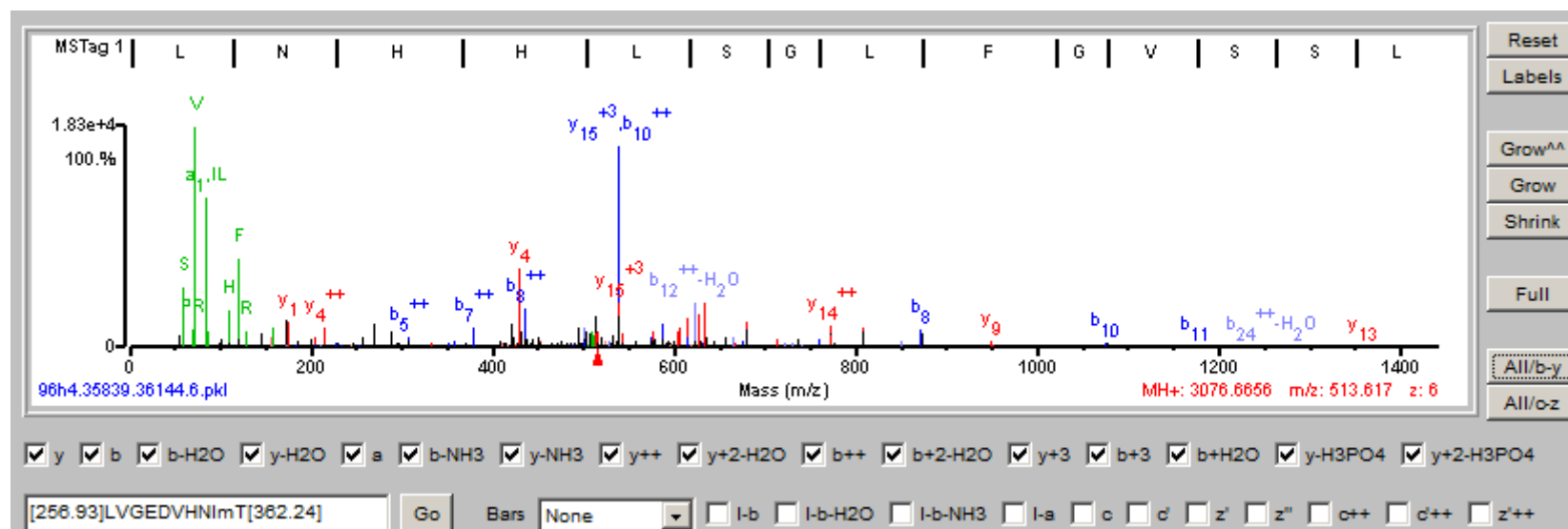

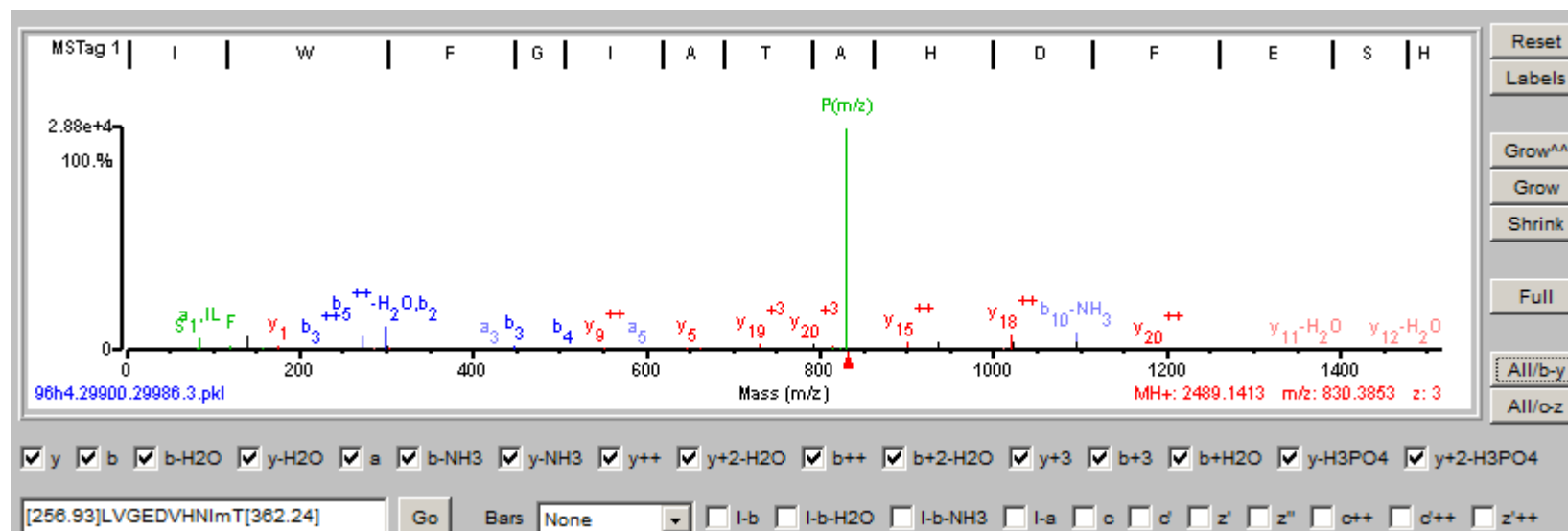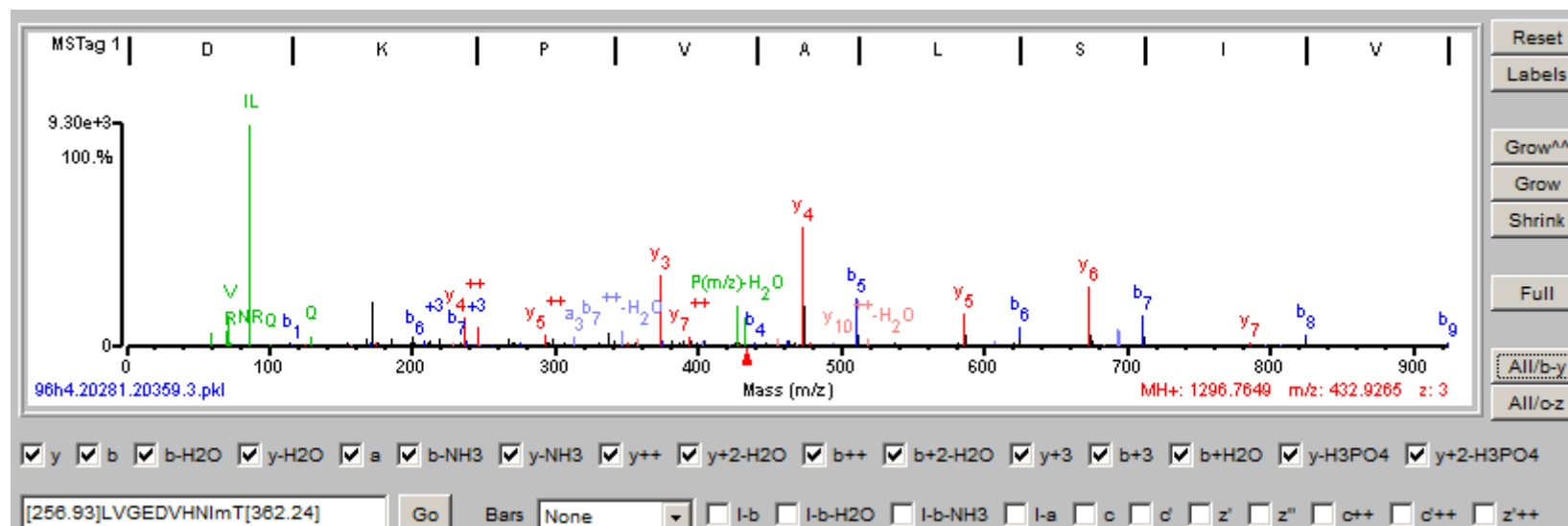

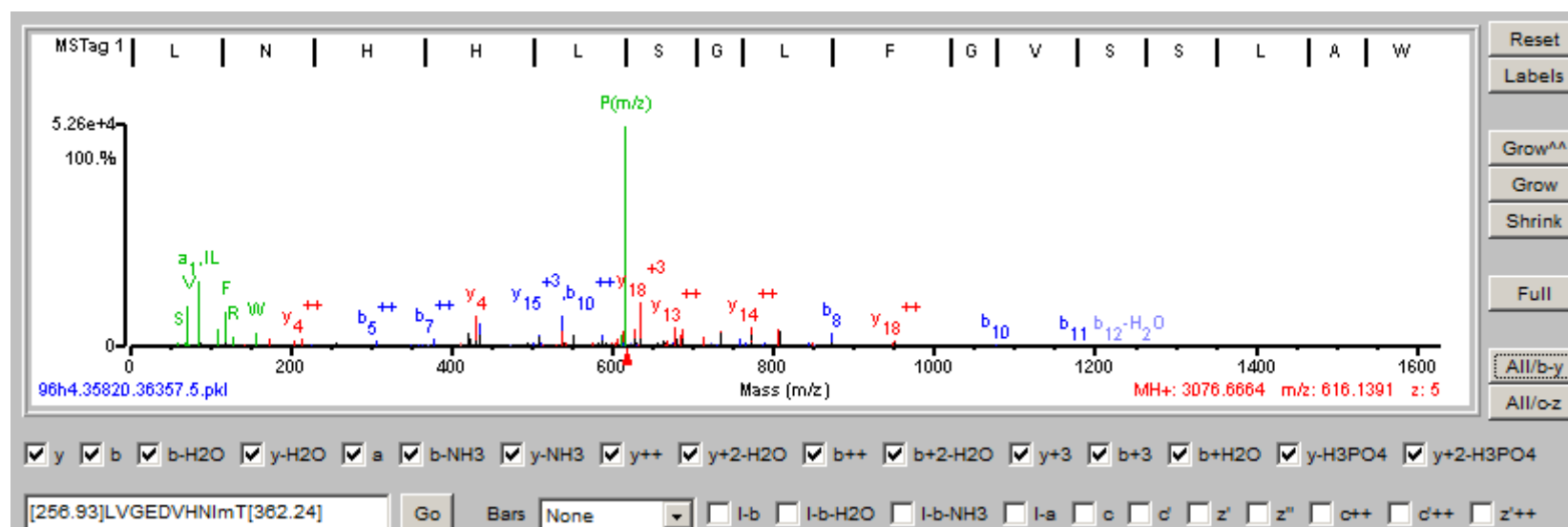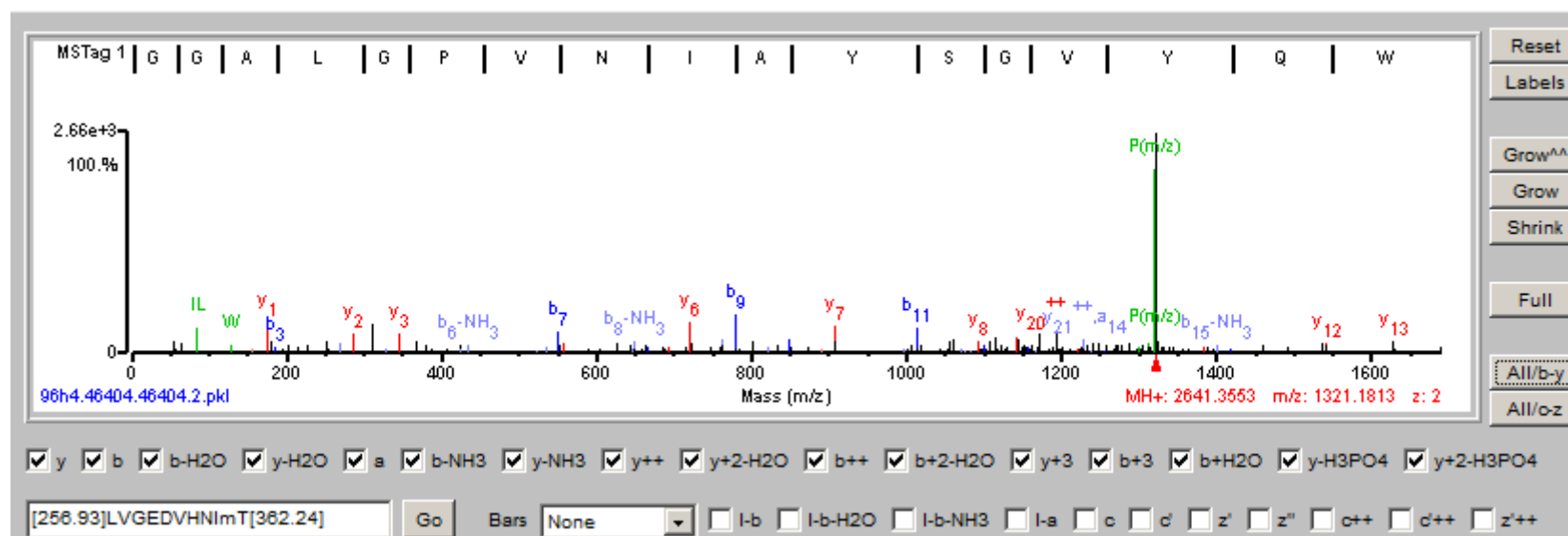

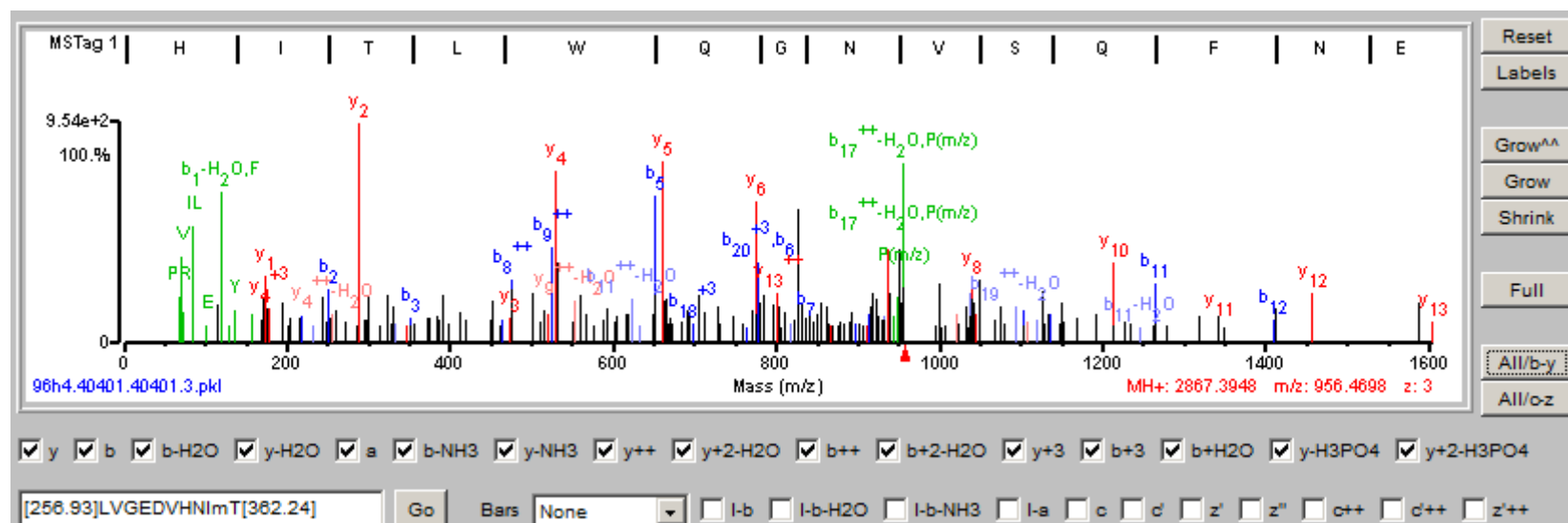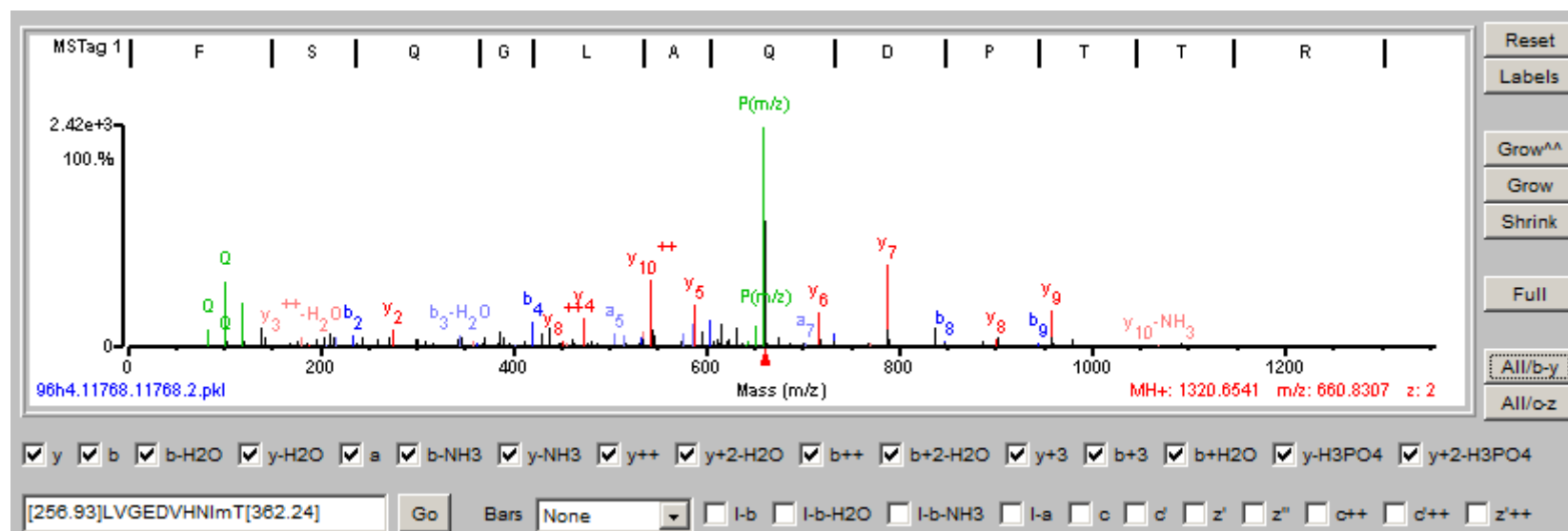

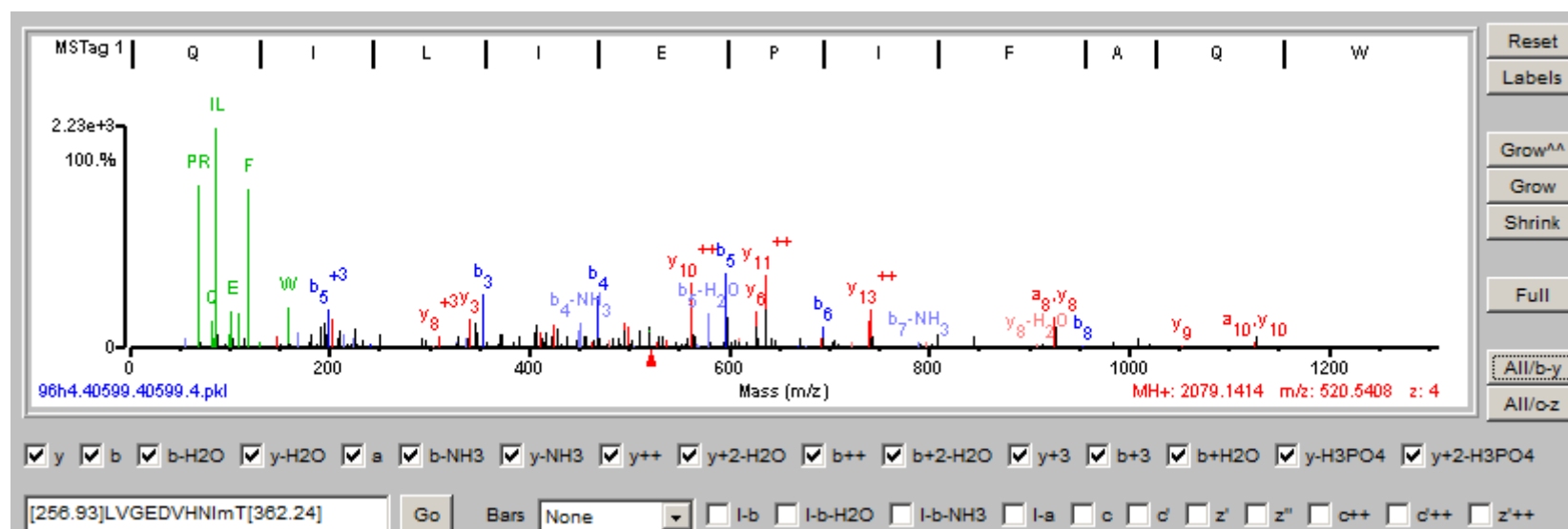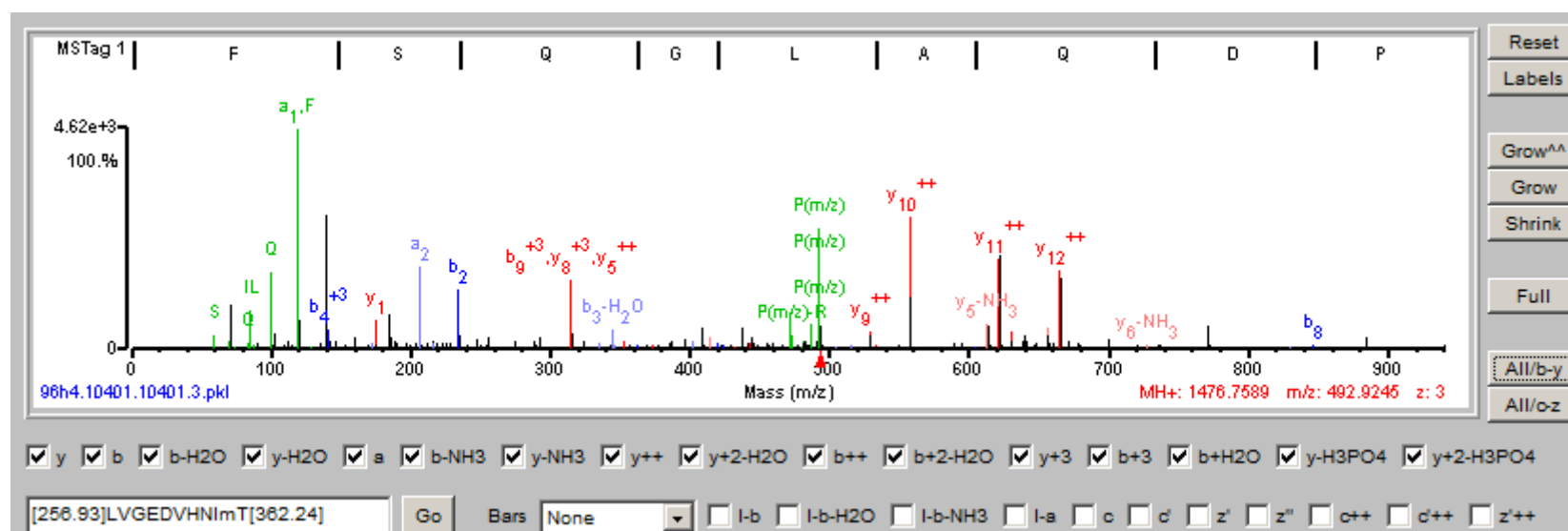

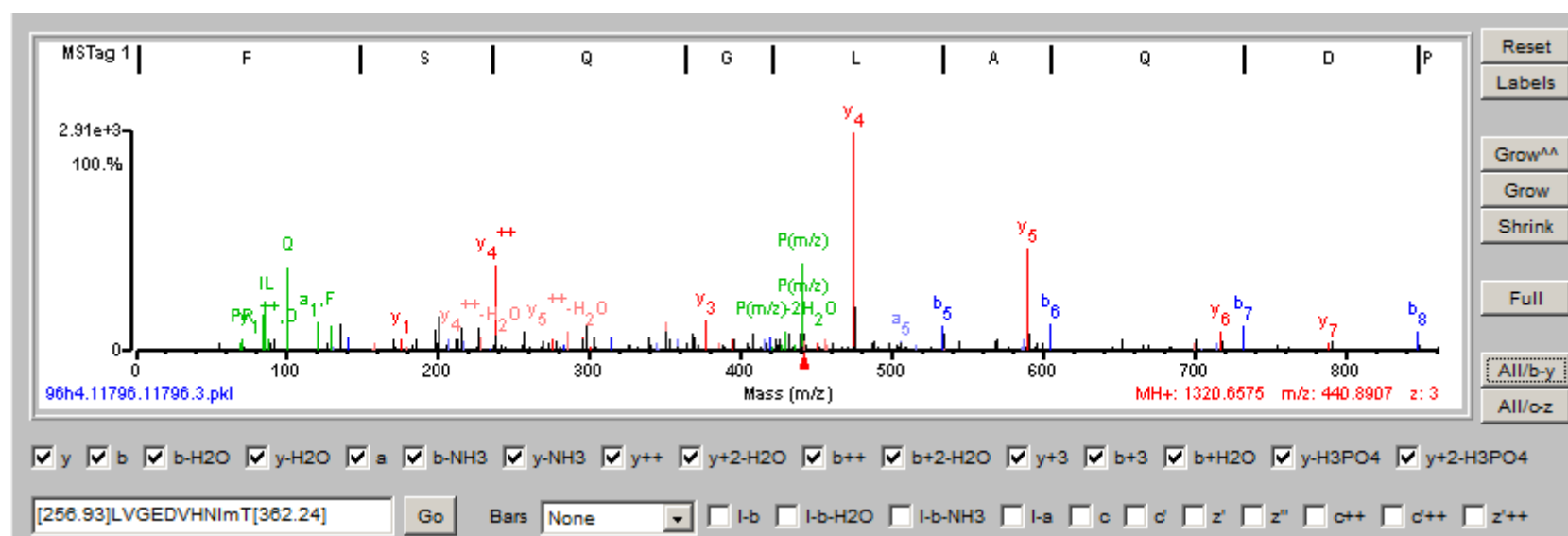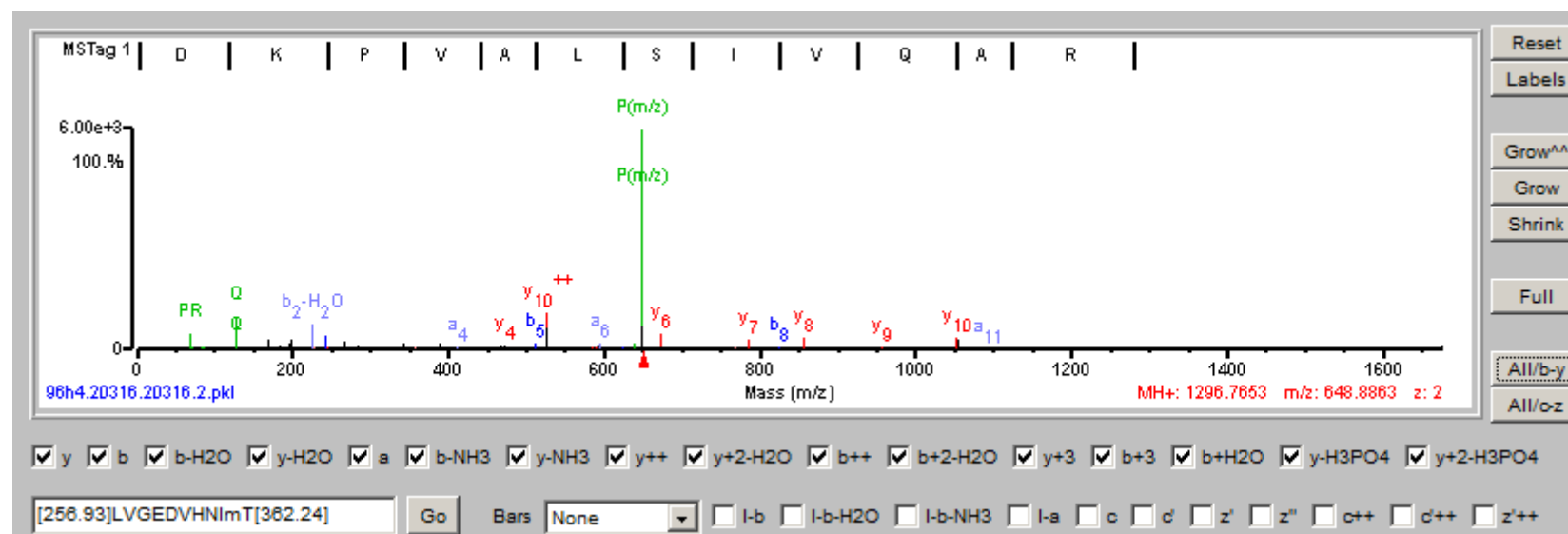

XP\_016537888.1

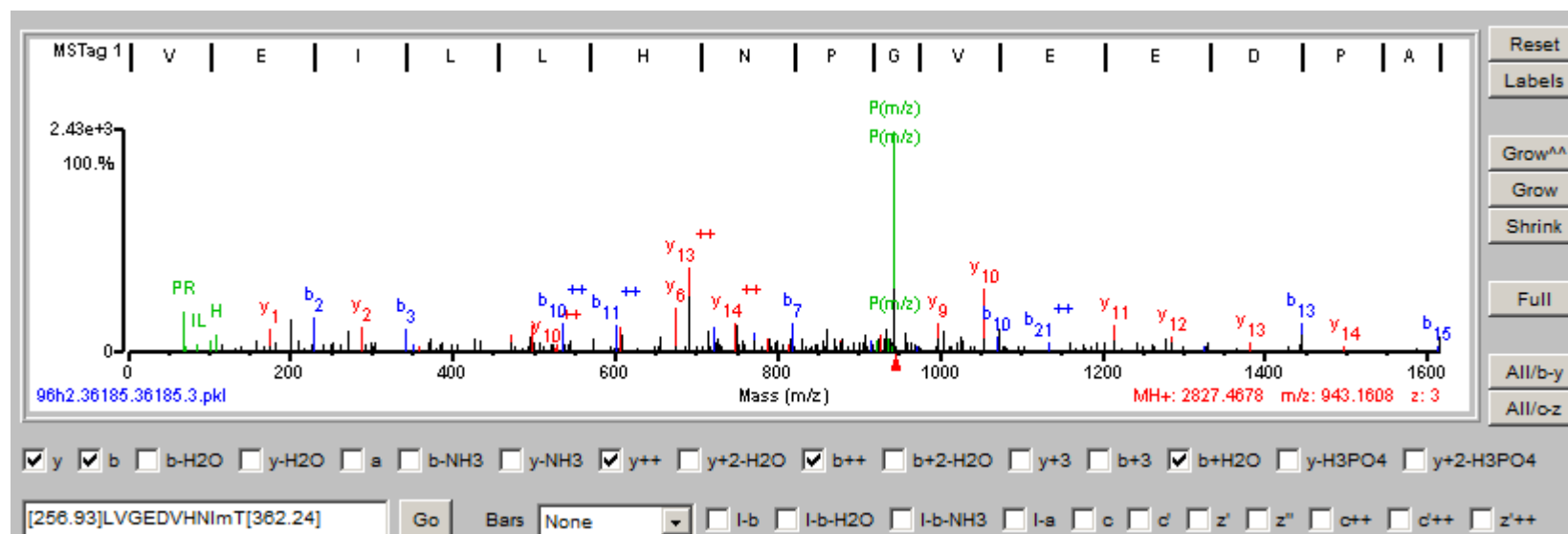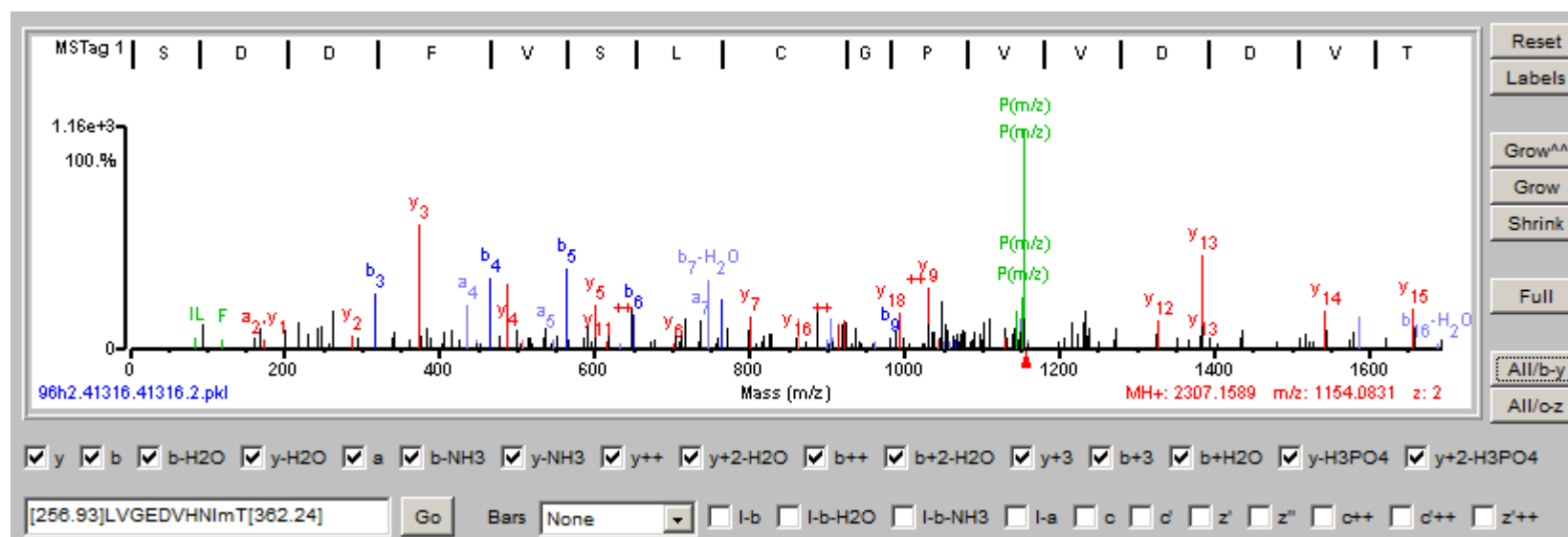

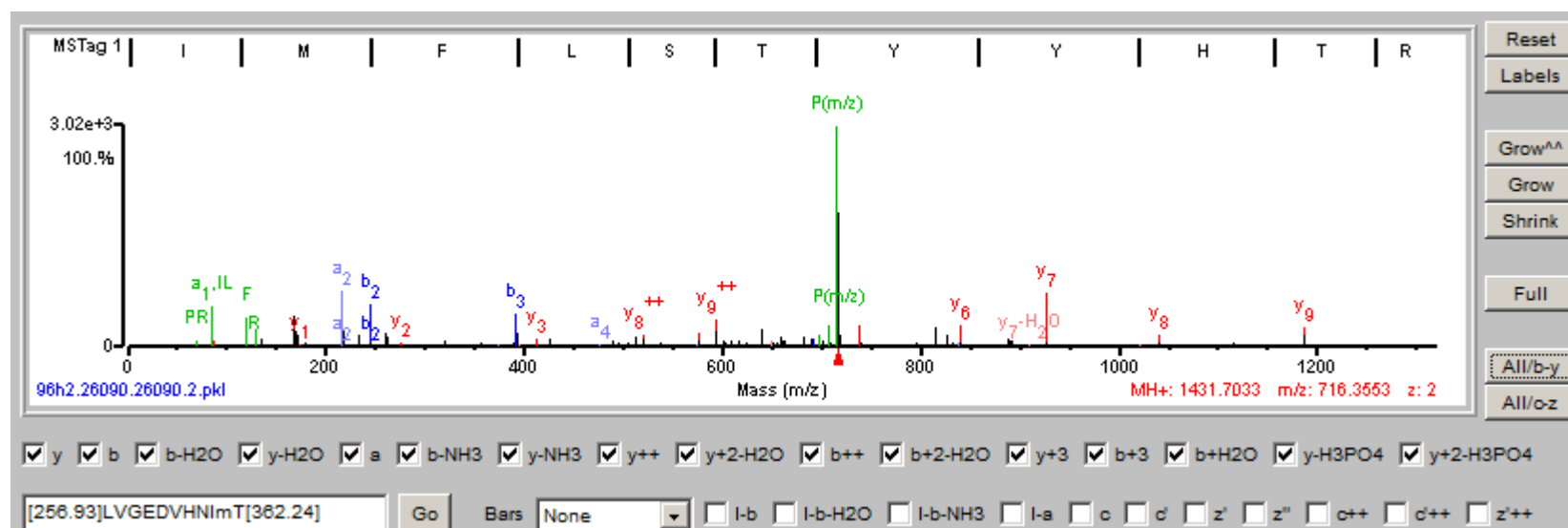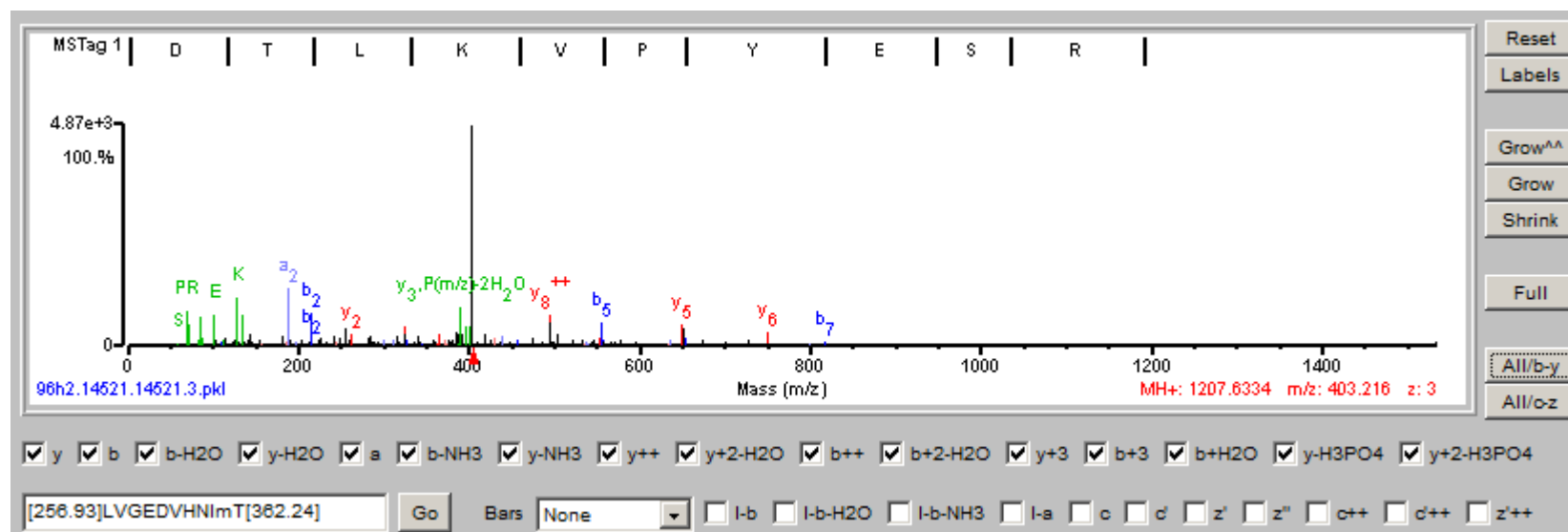

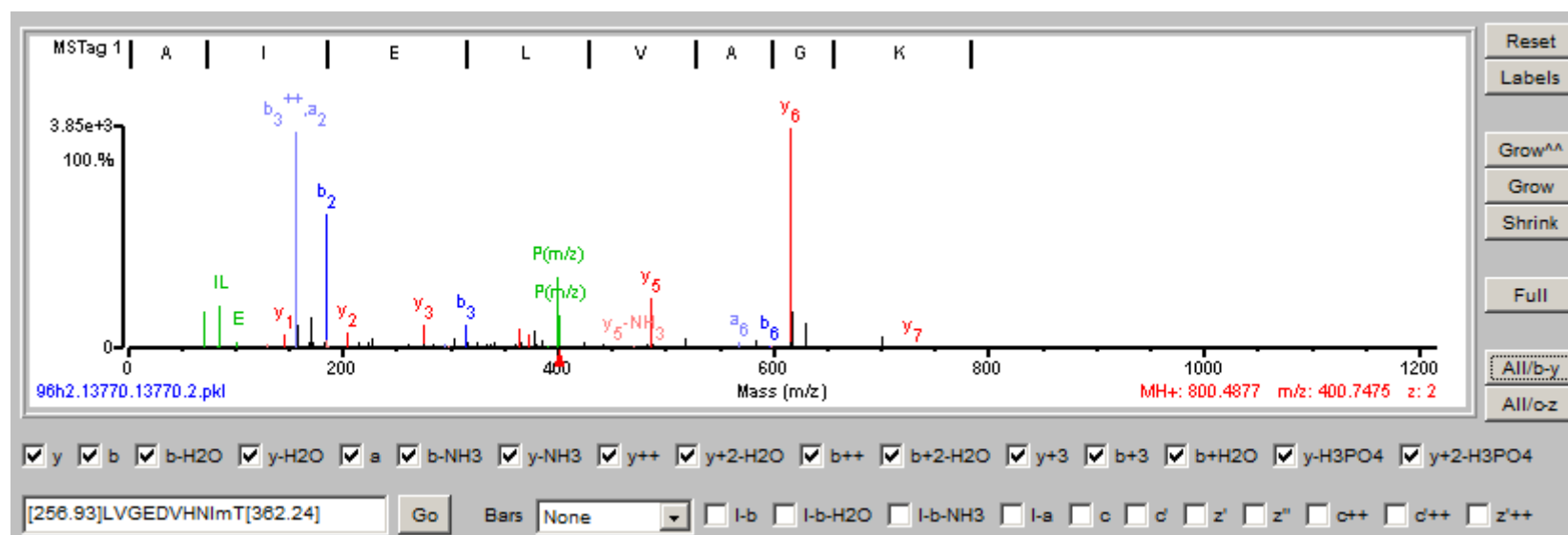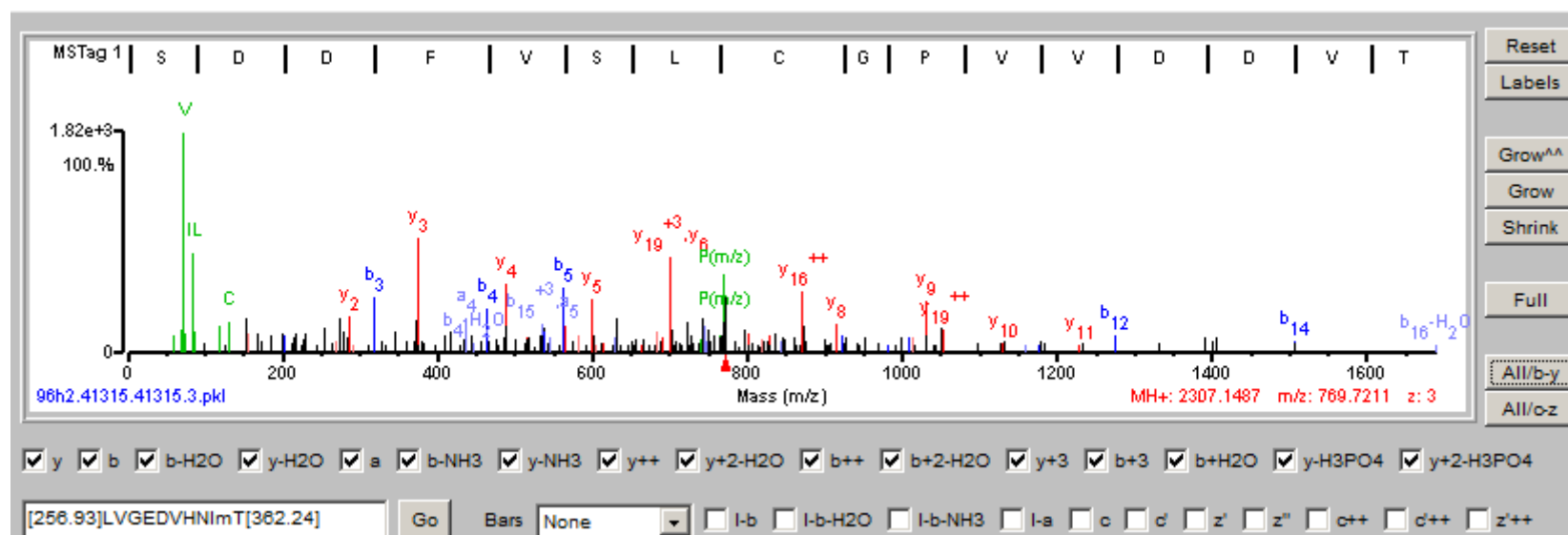

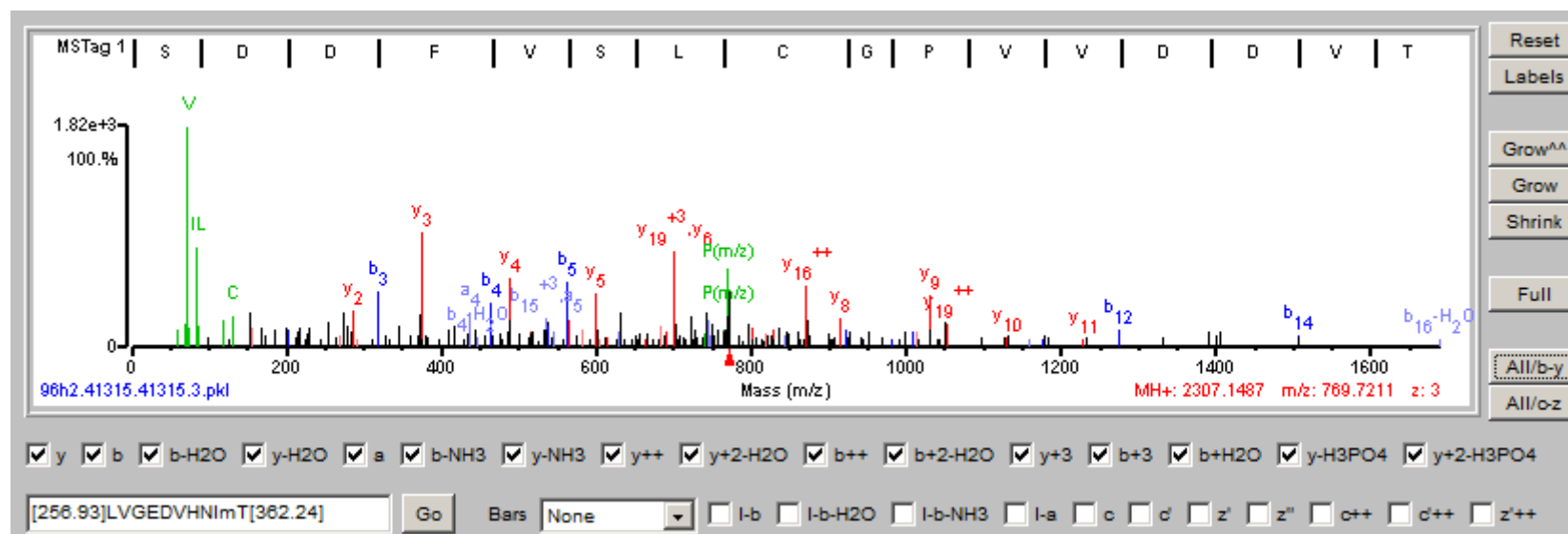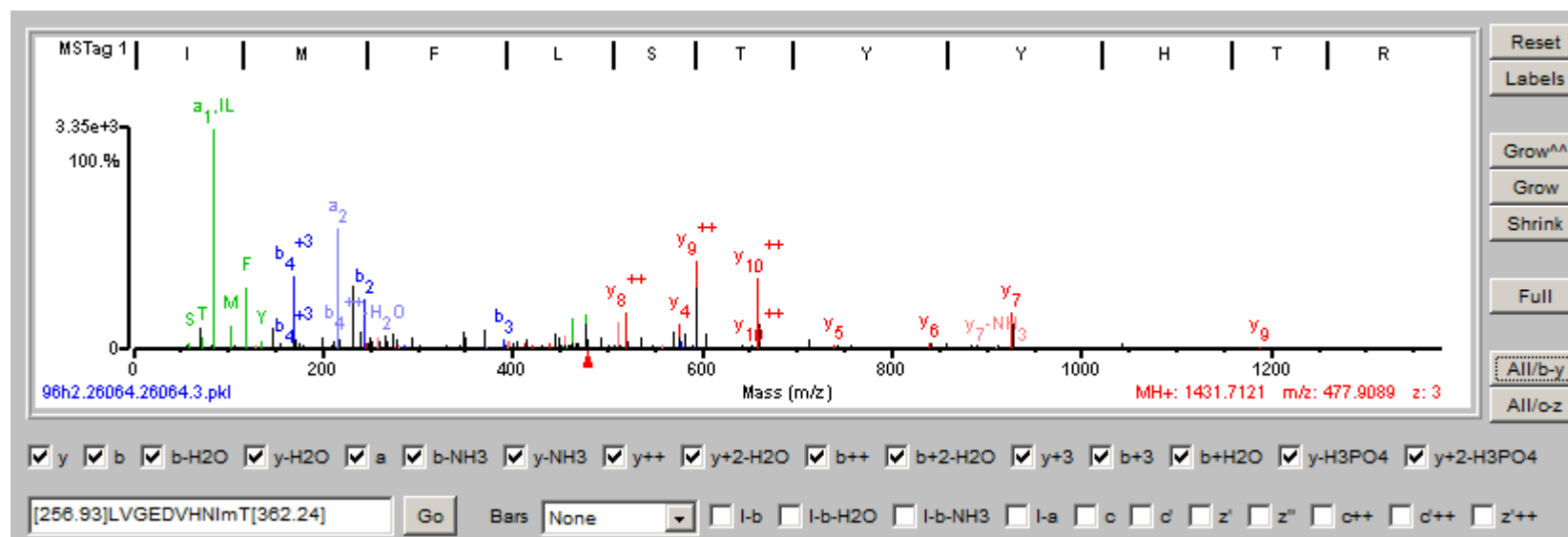

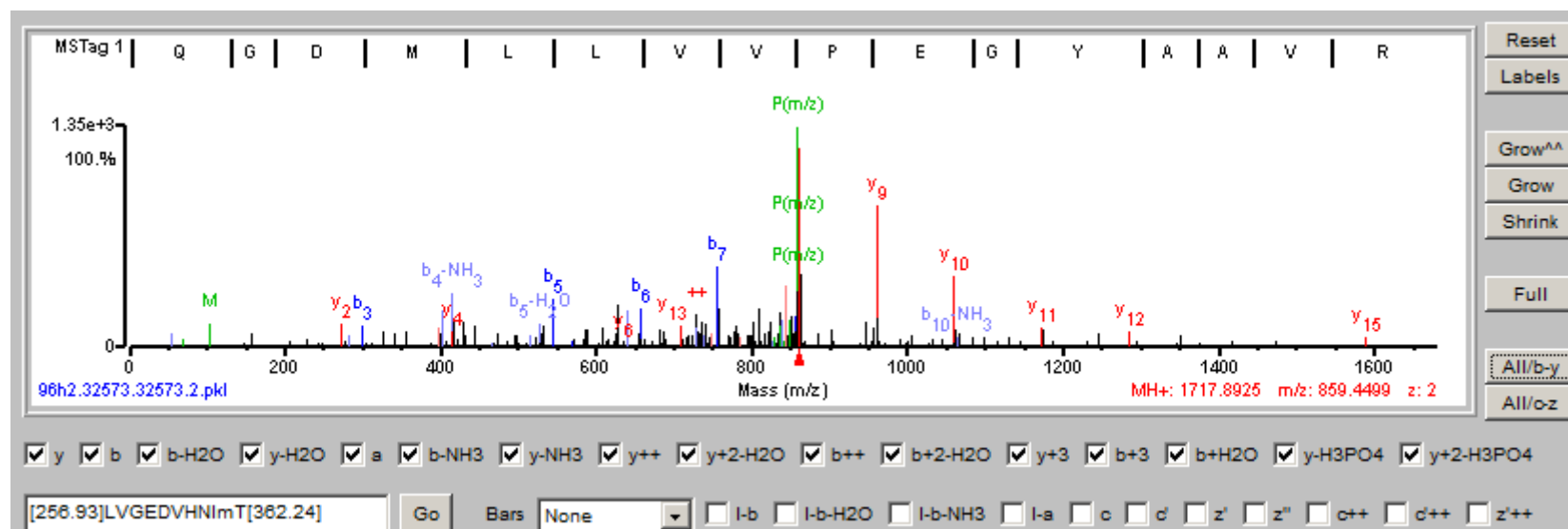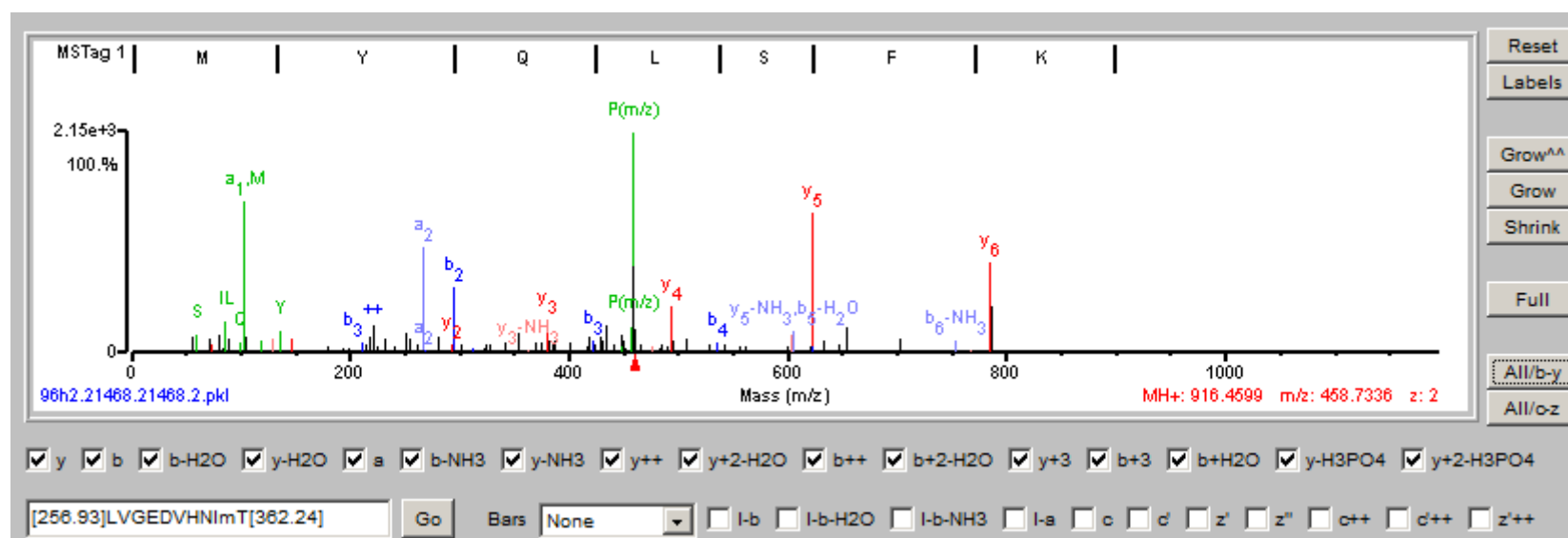

XP\_016543302.1

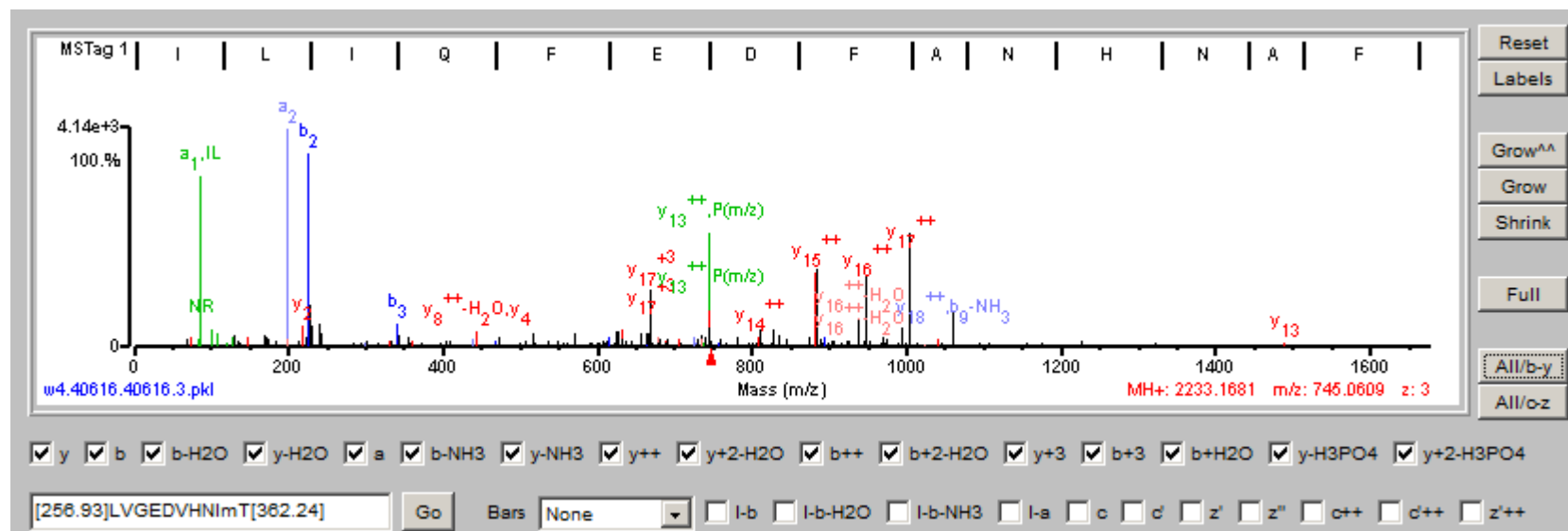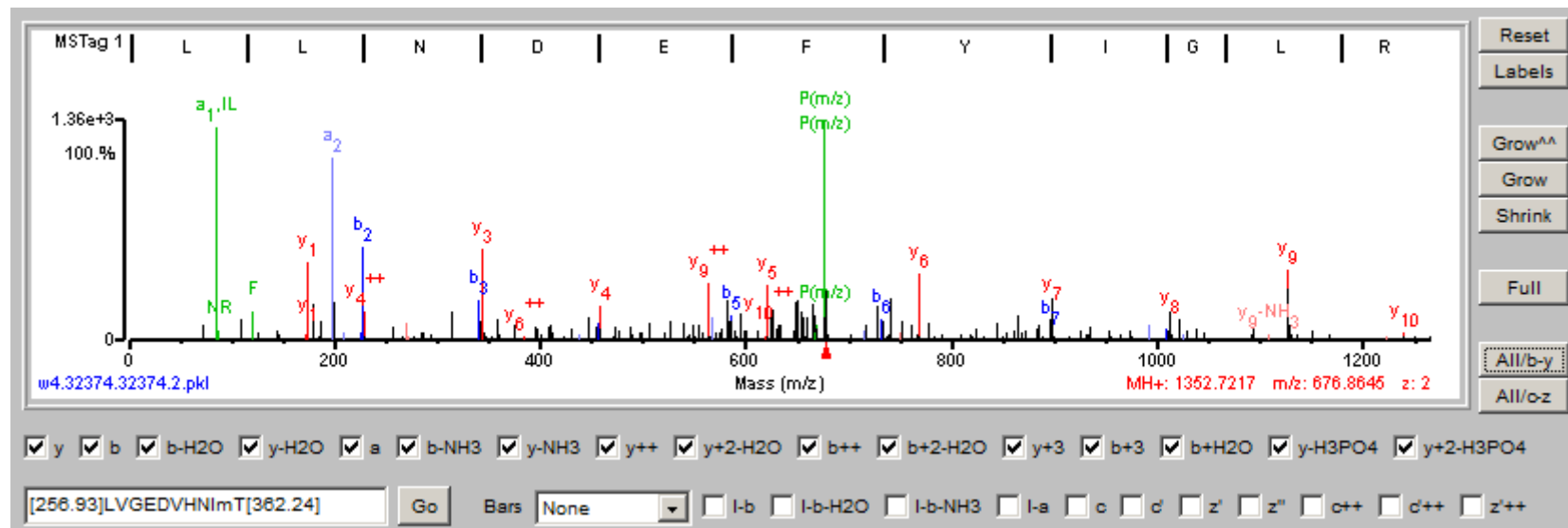

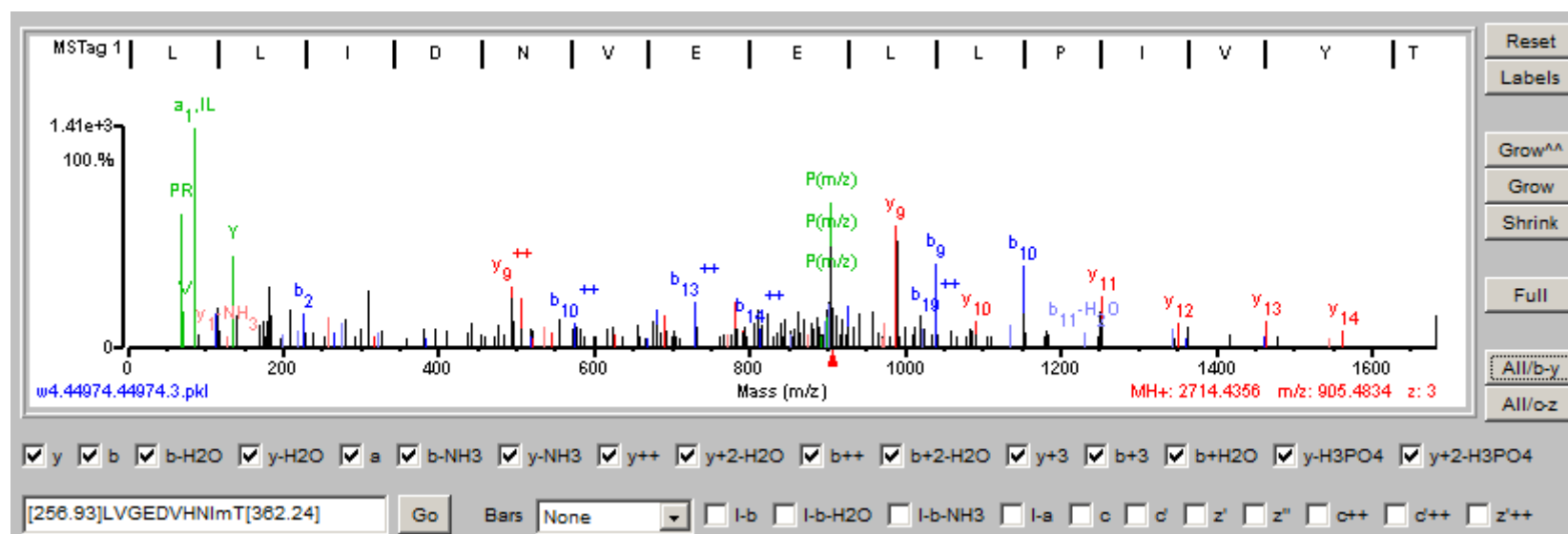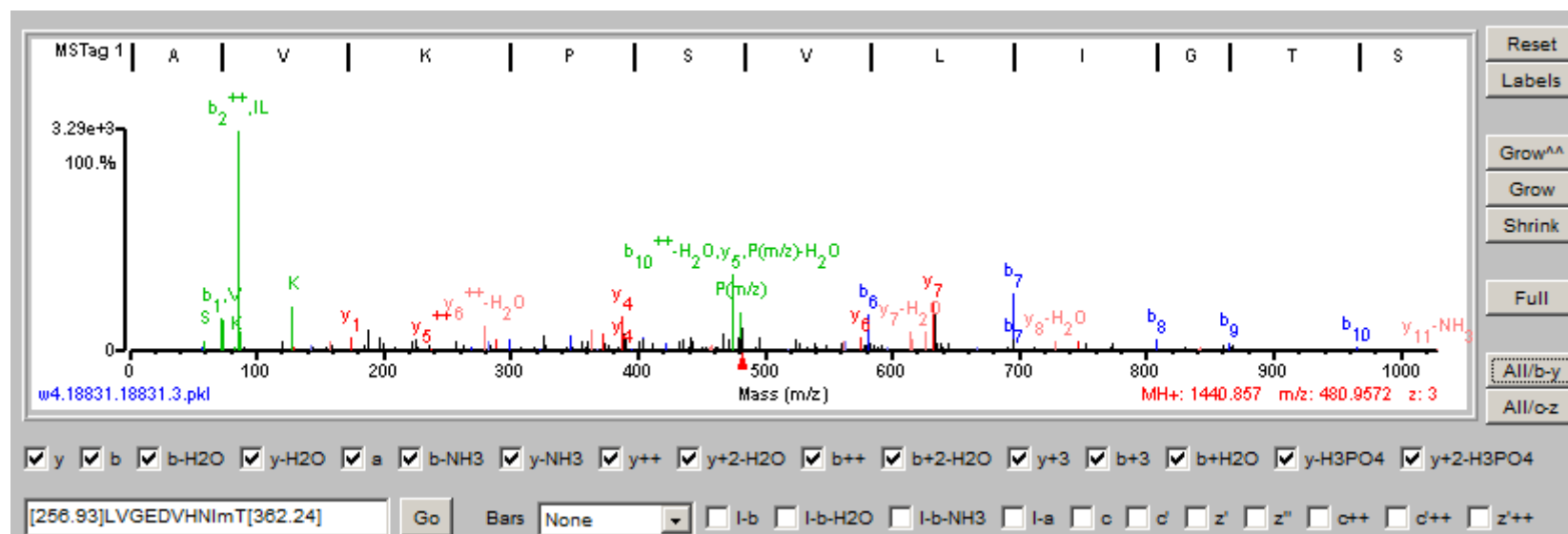

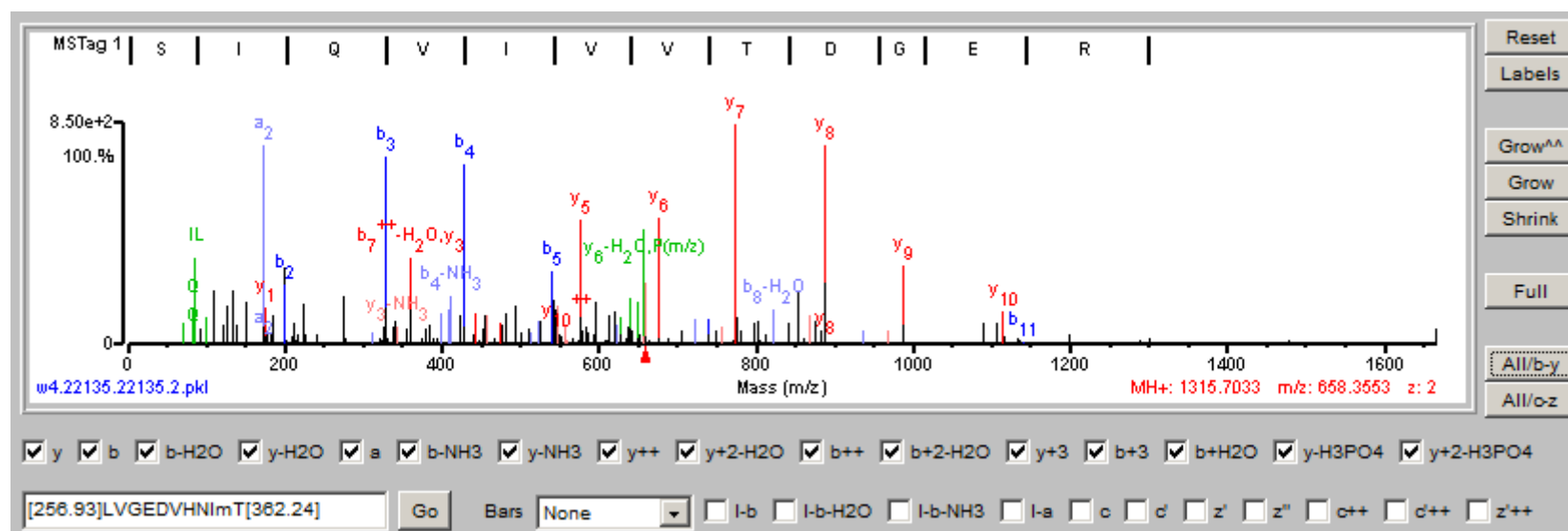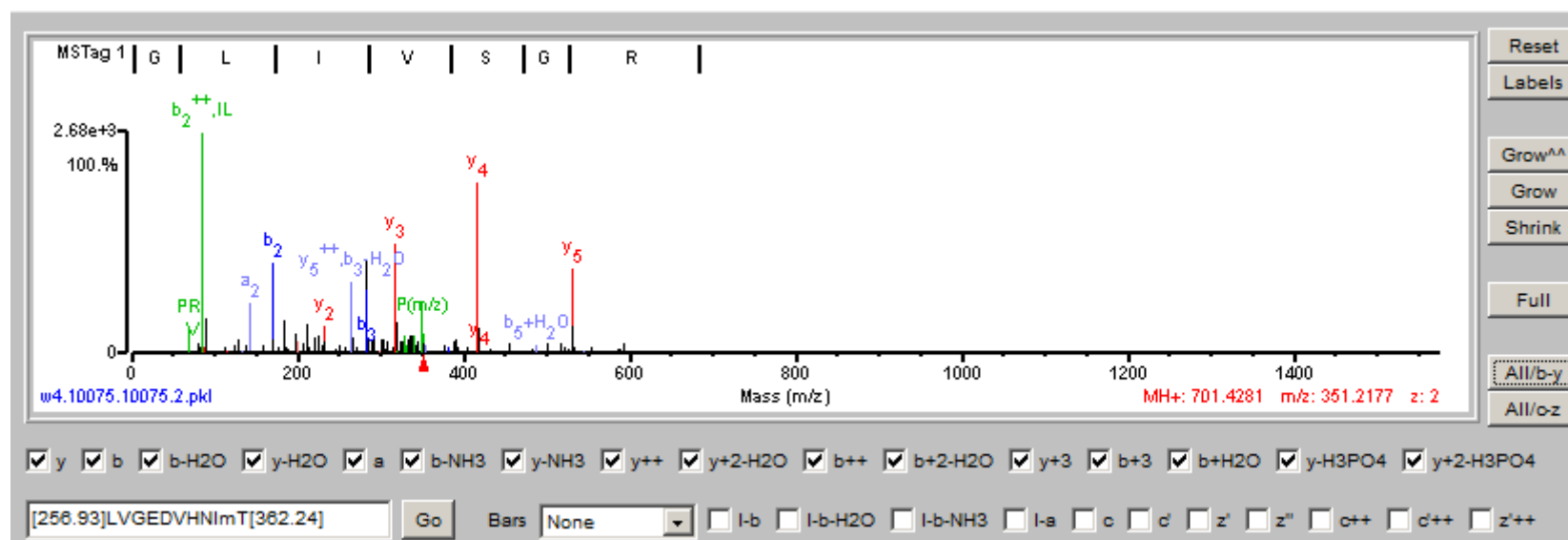

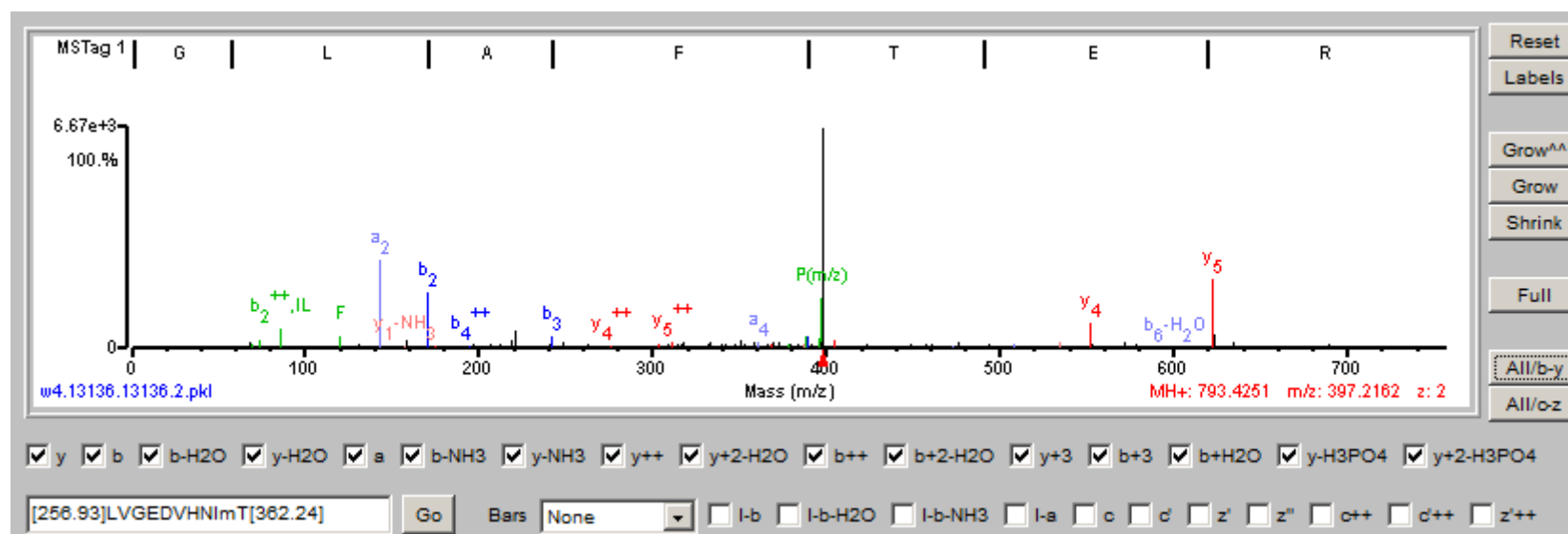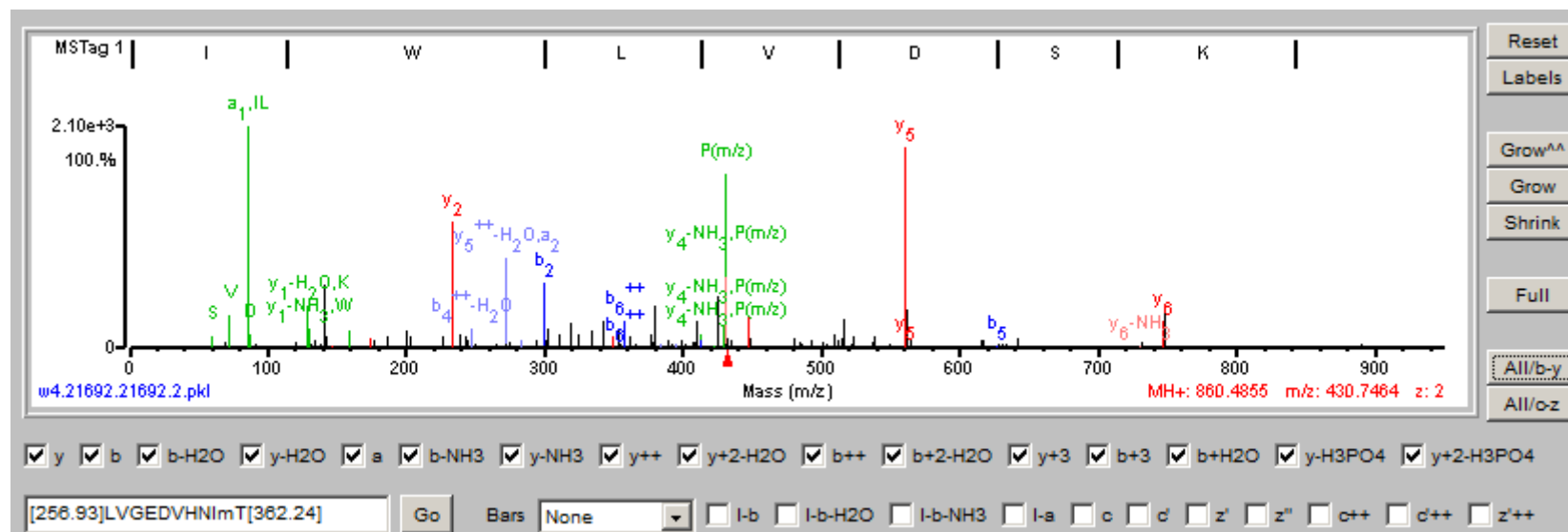

XP\_016547661.1

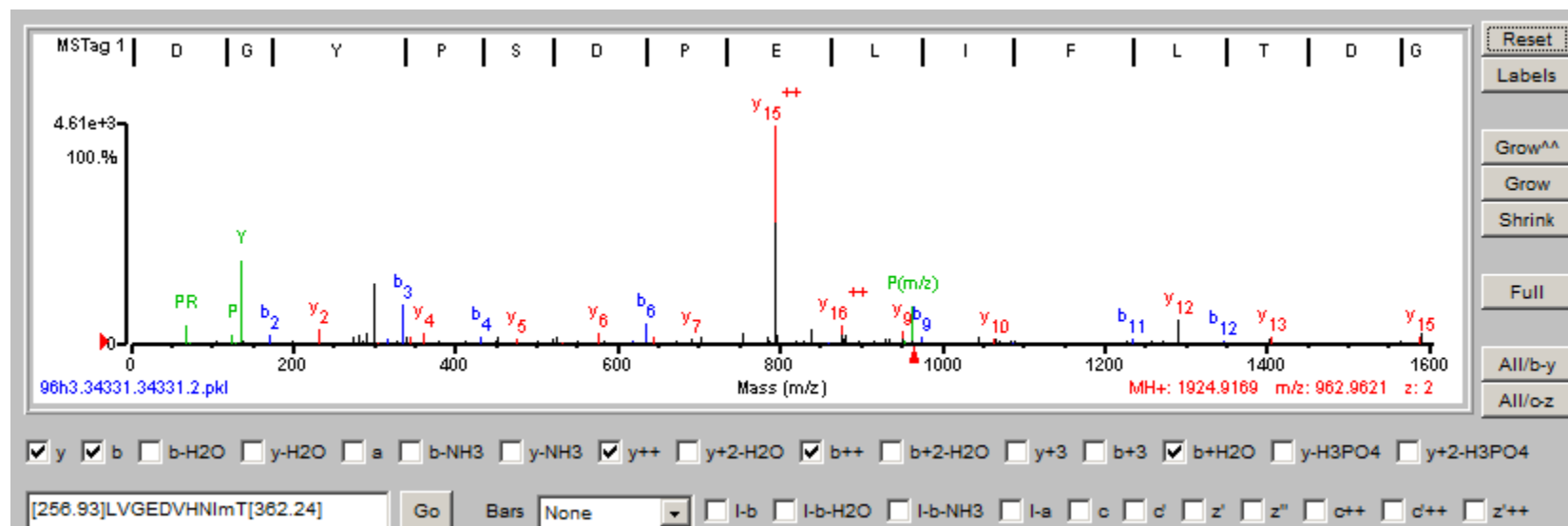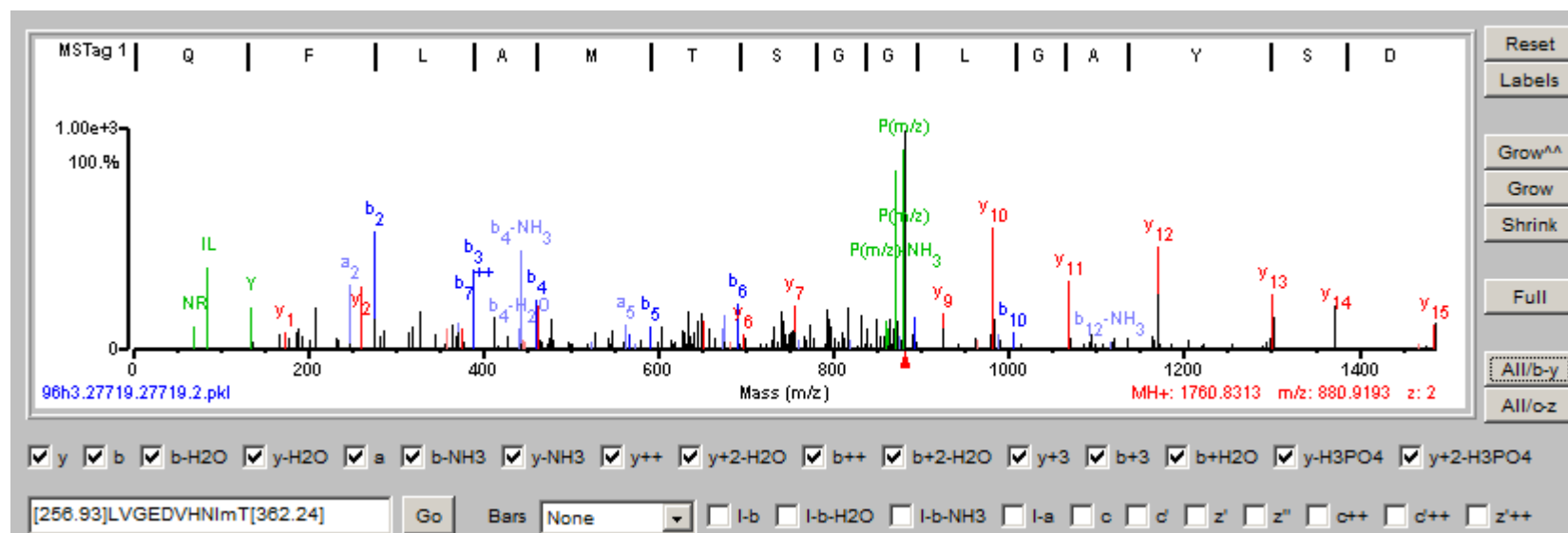

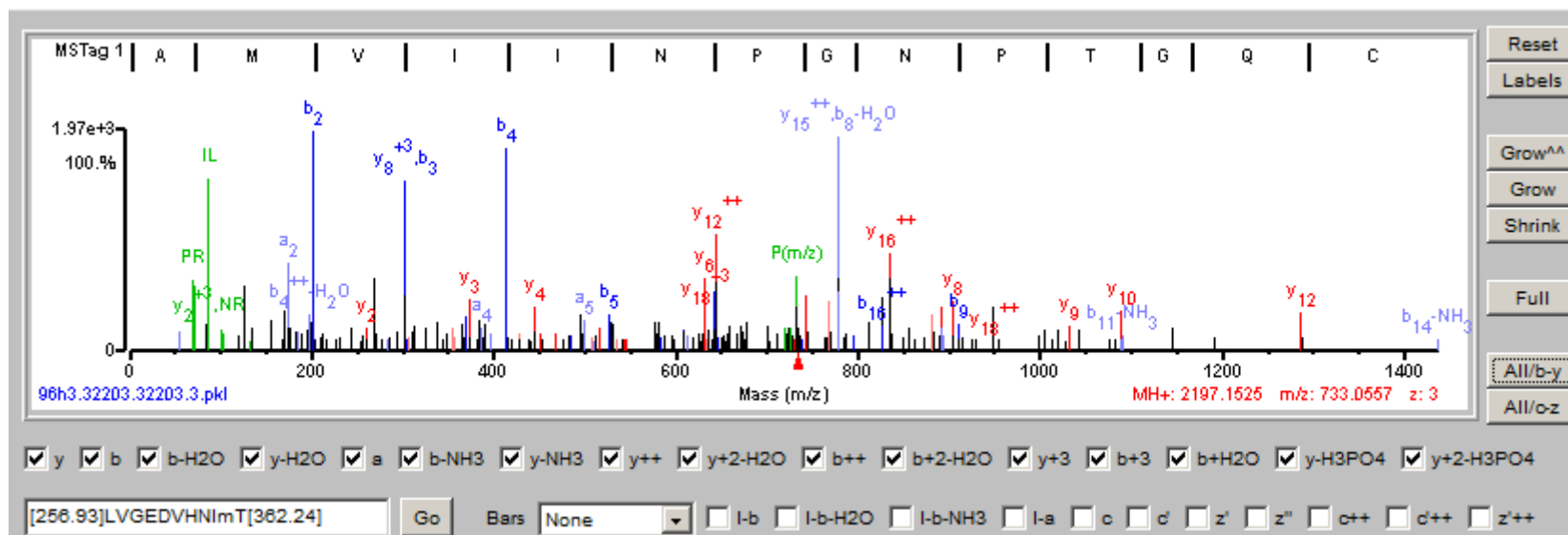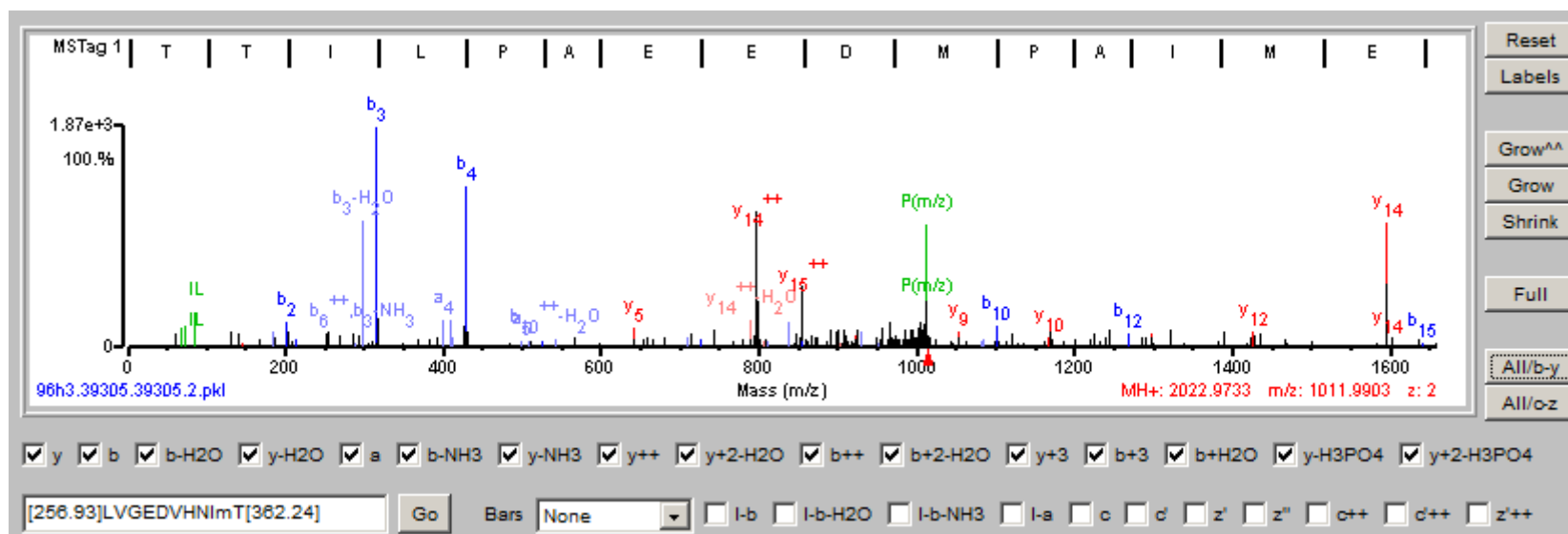

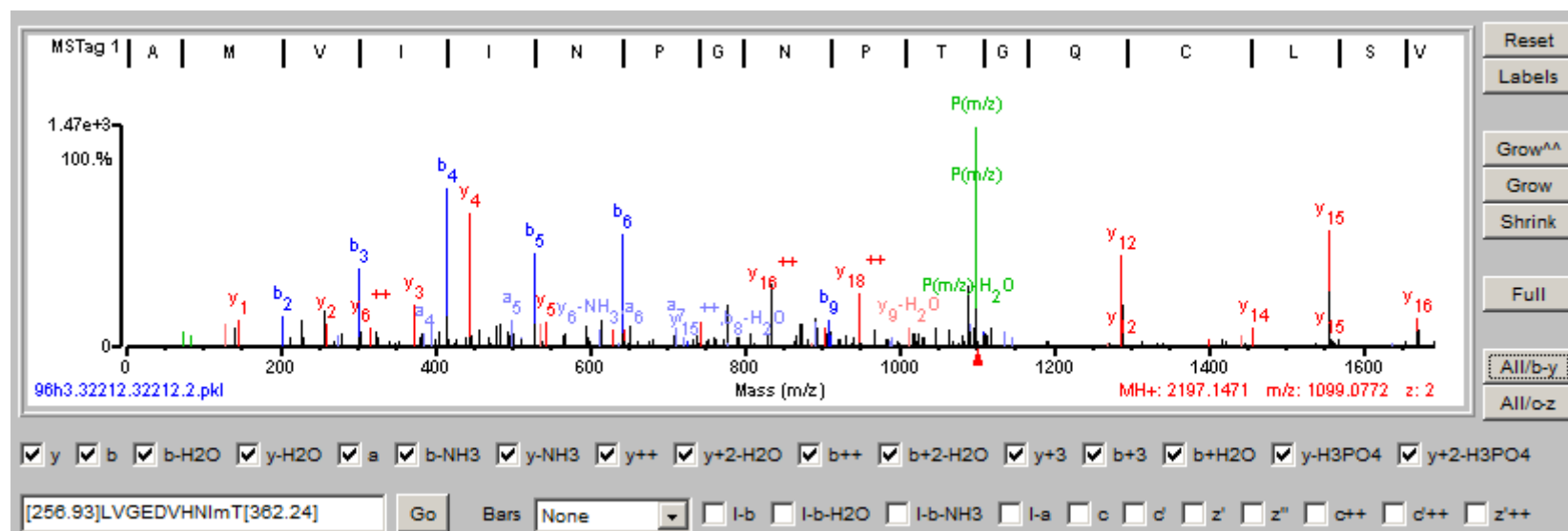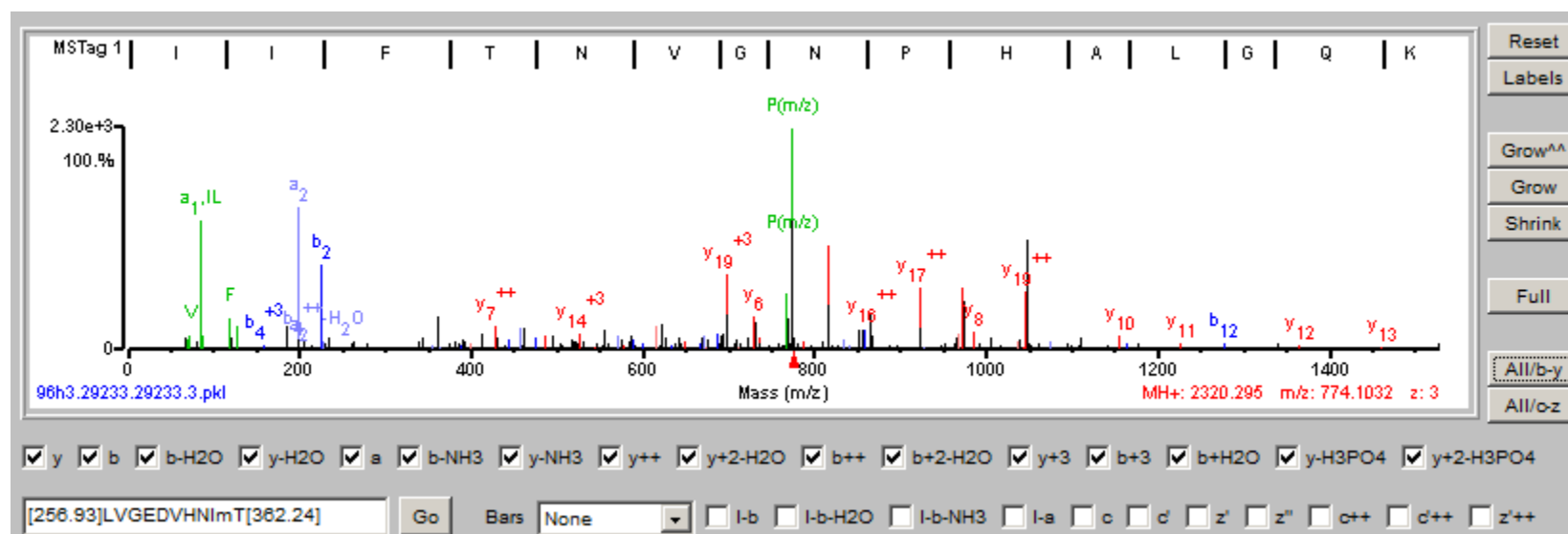

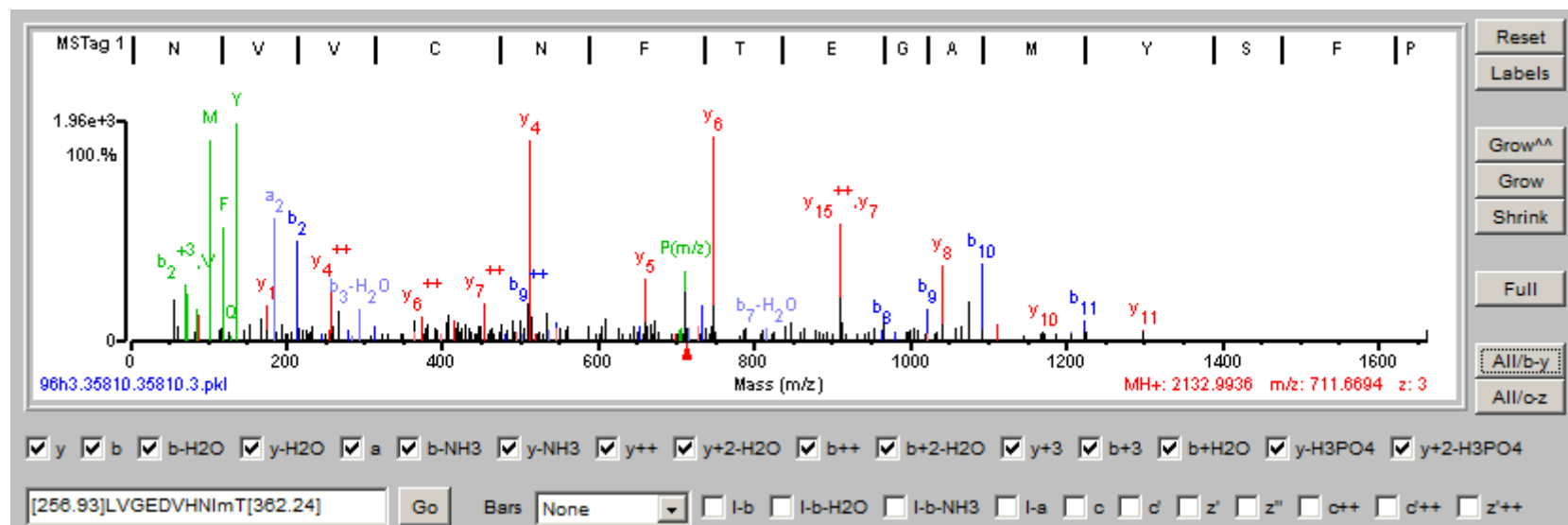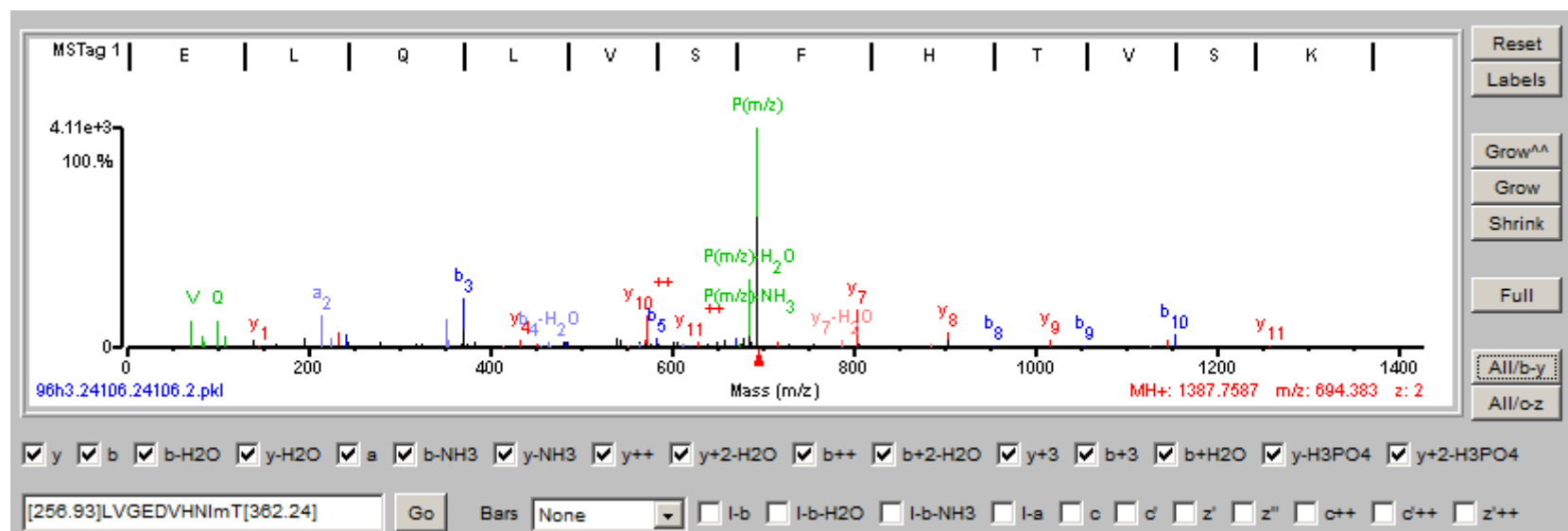

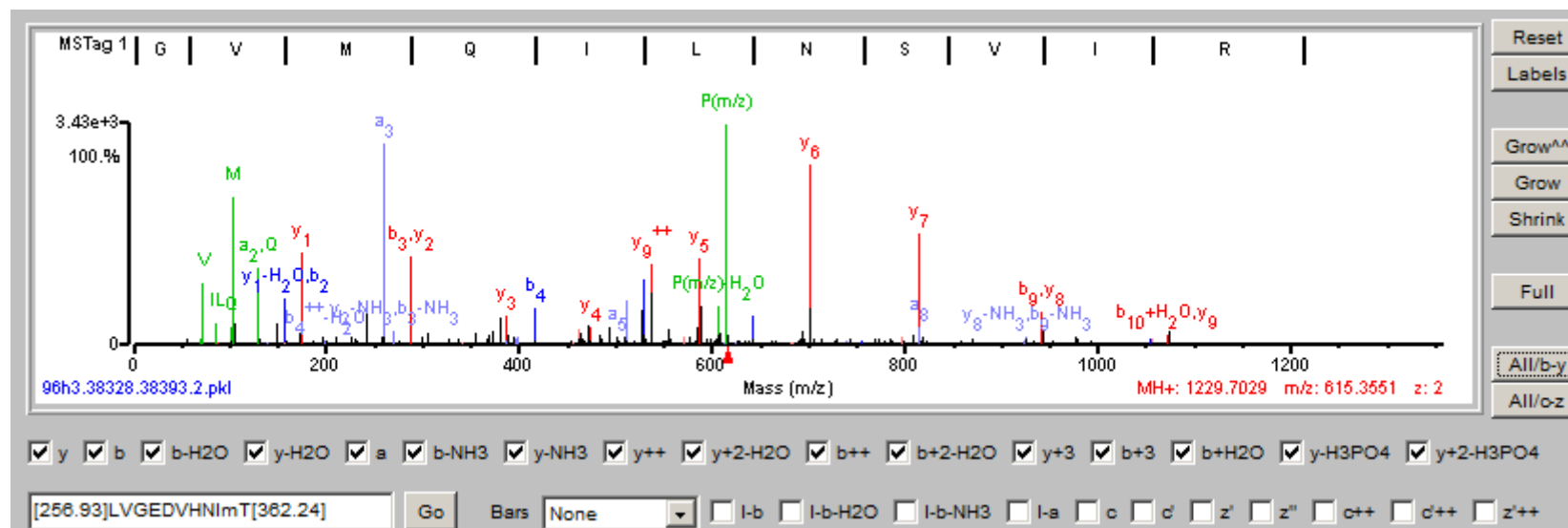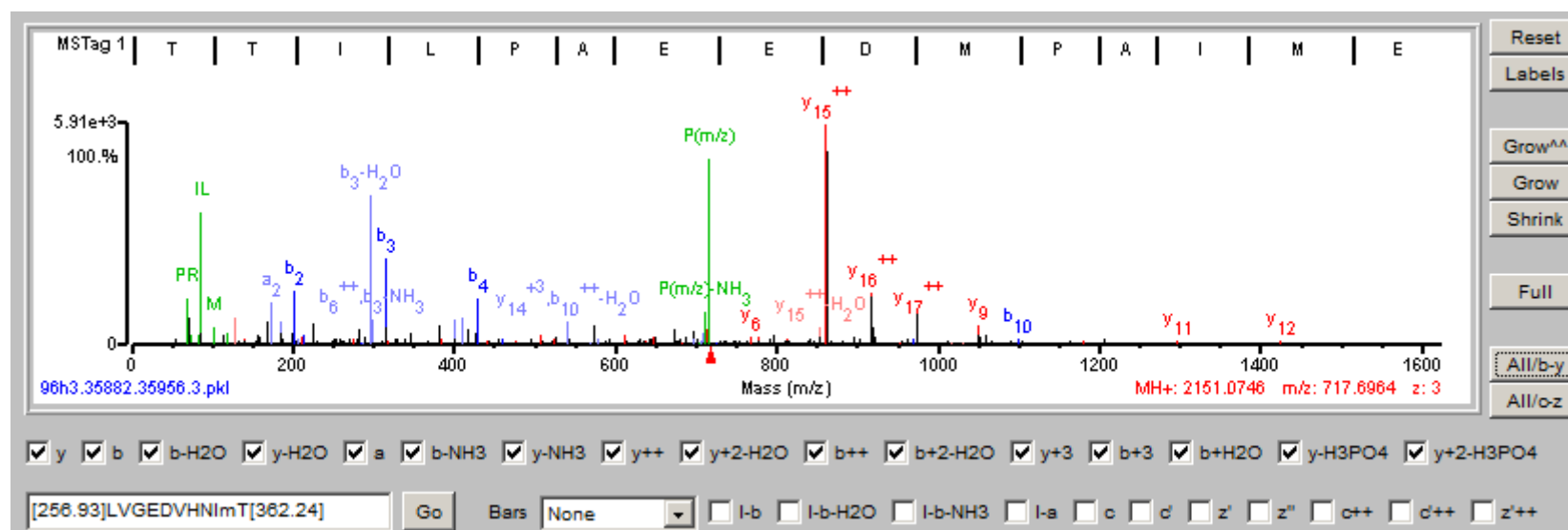

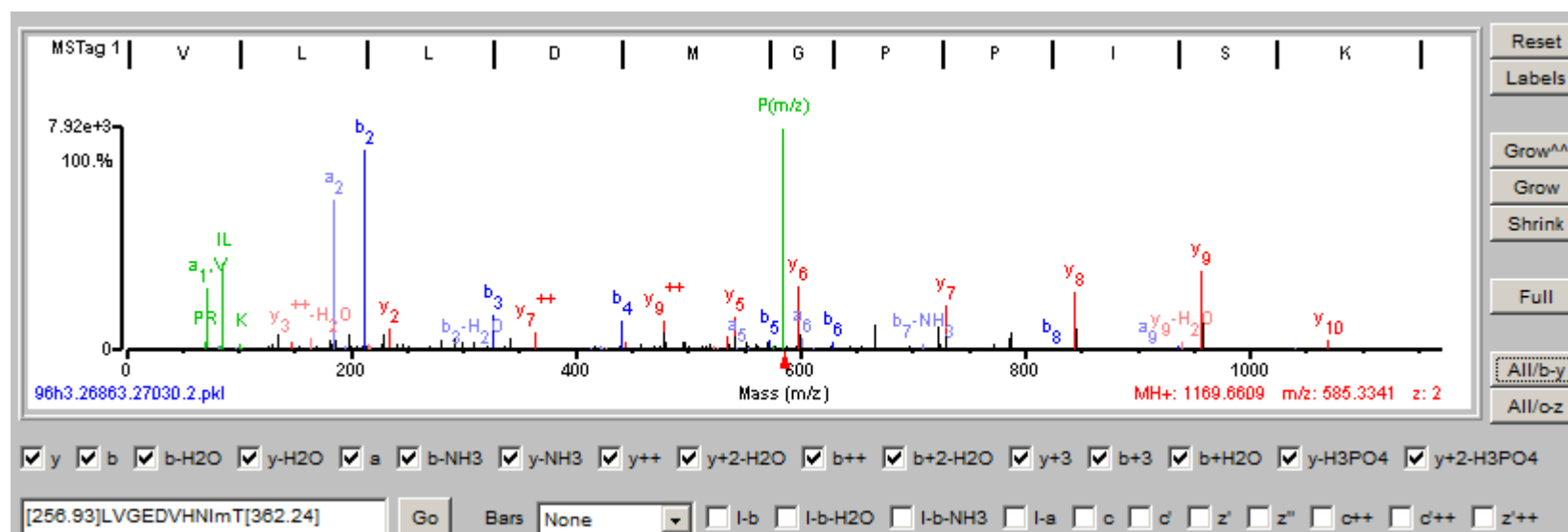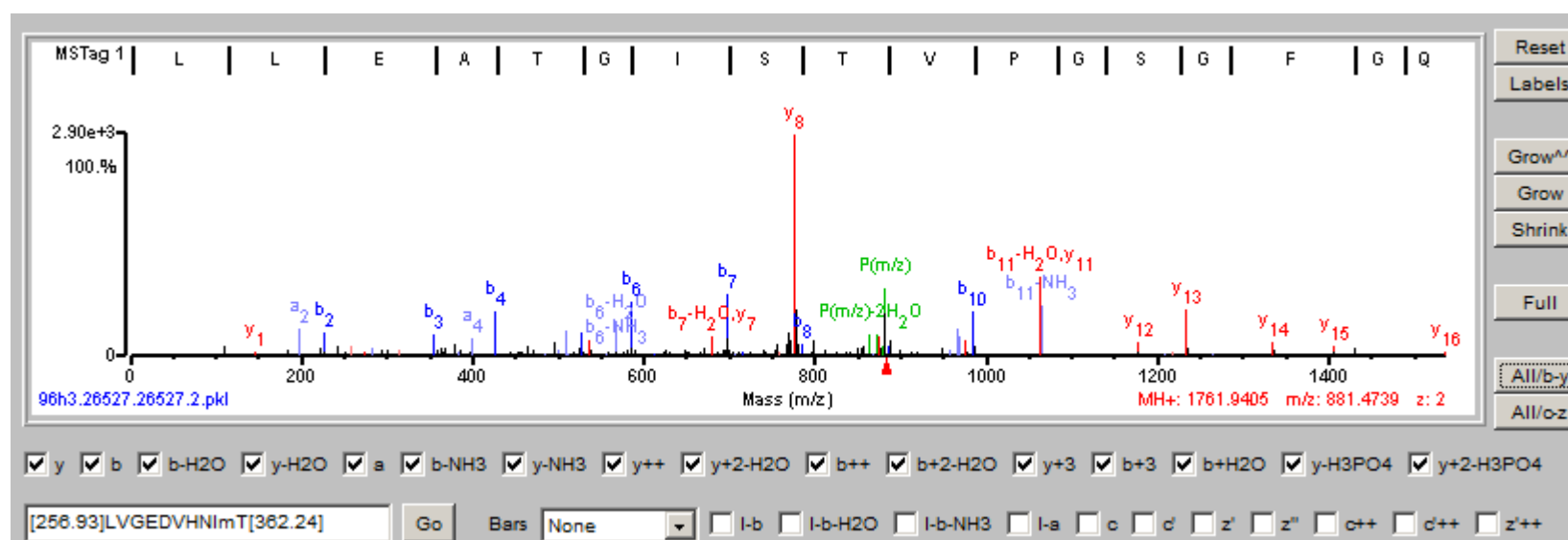

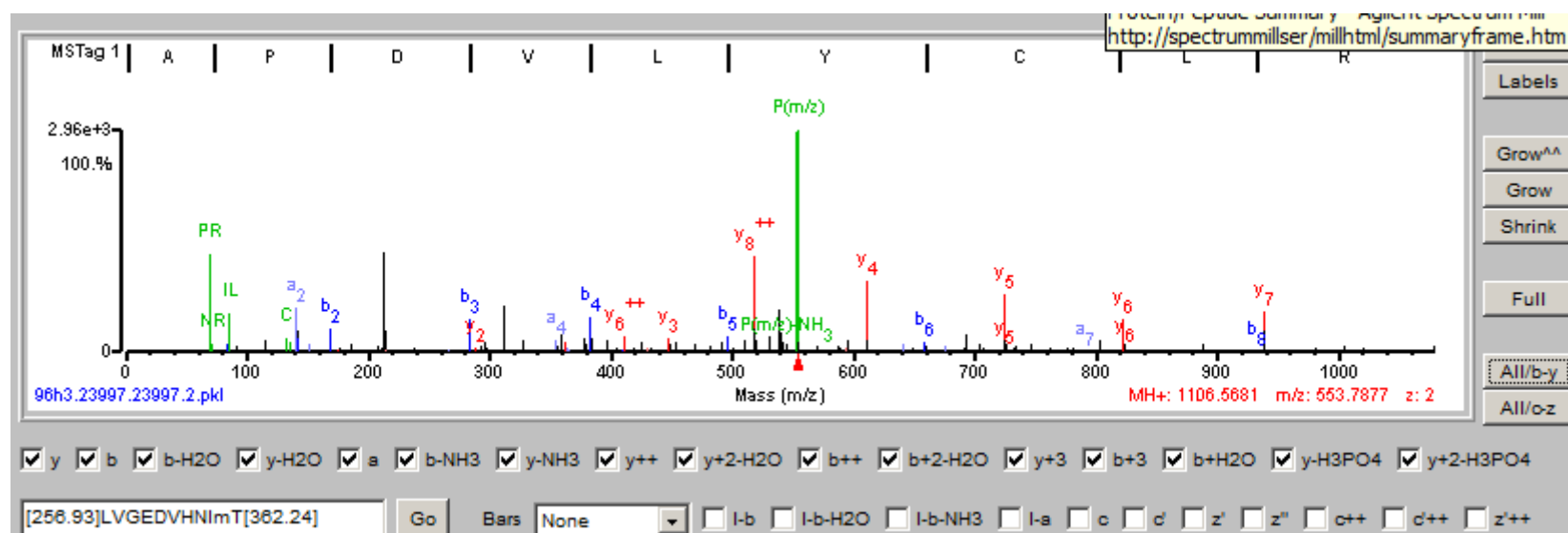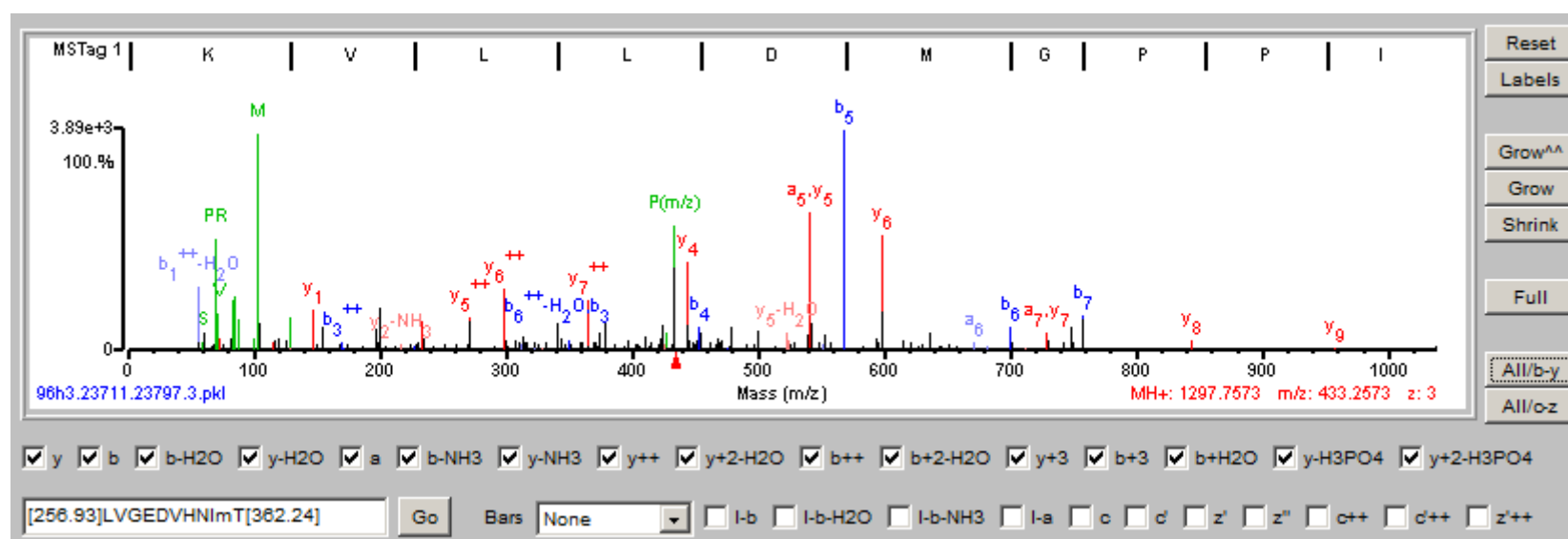

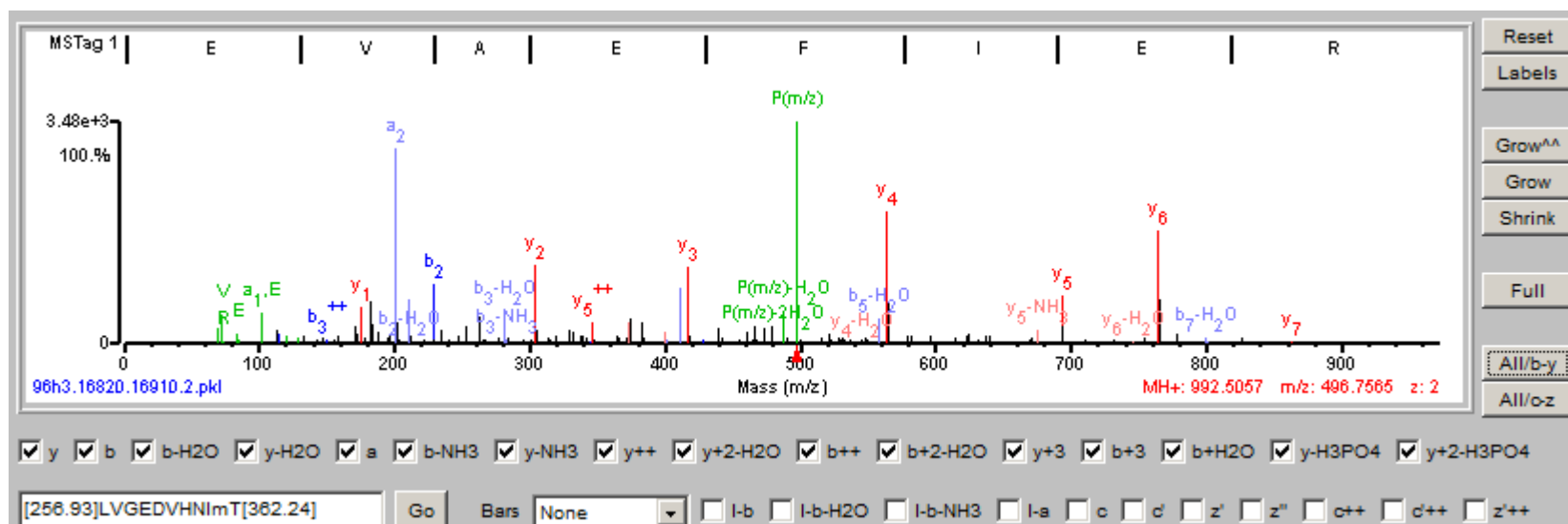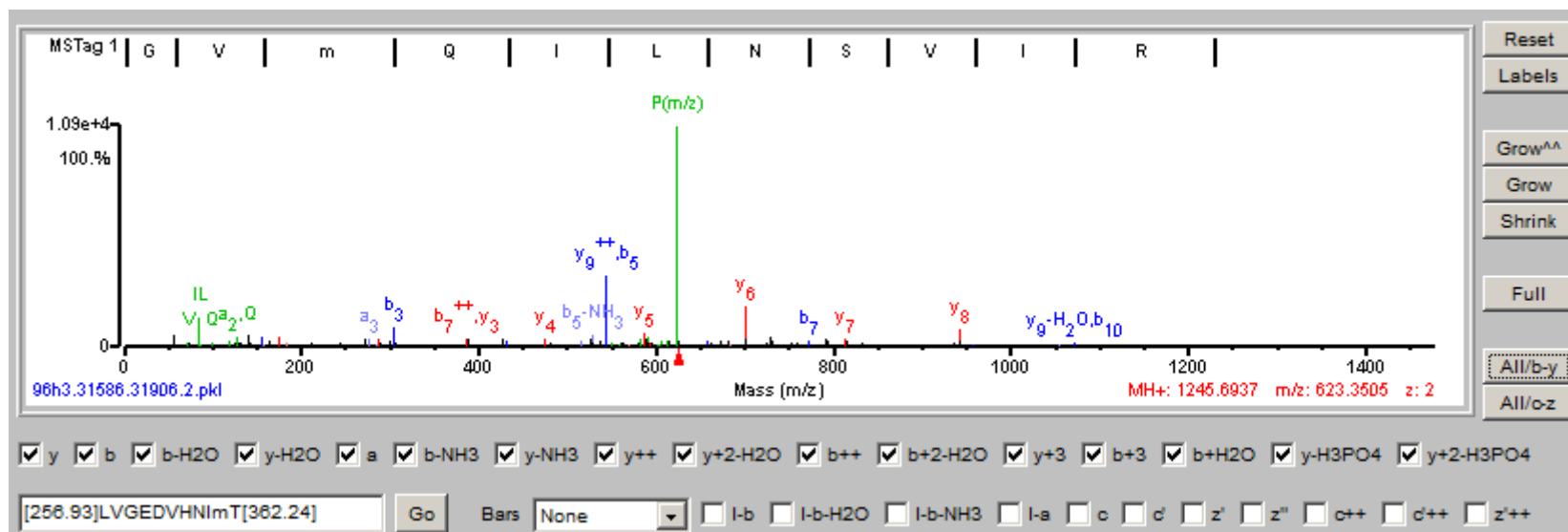



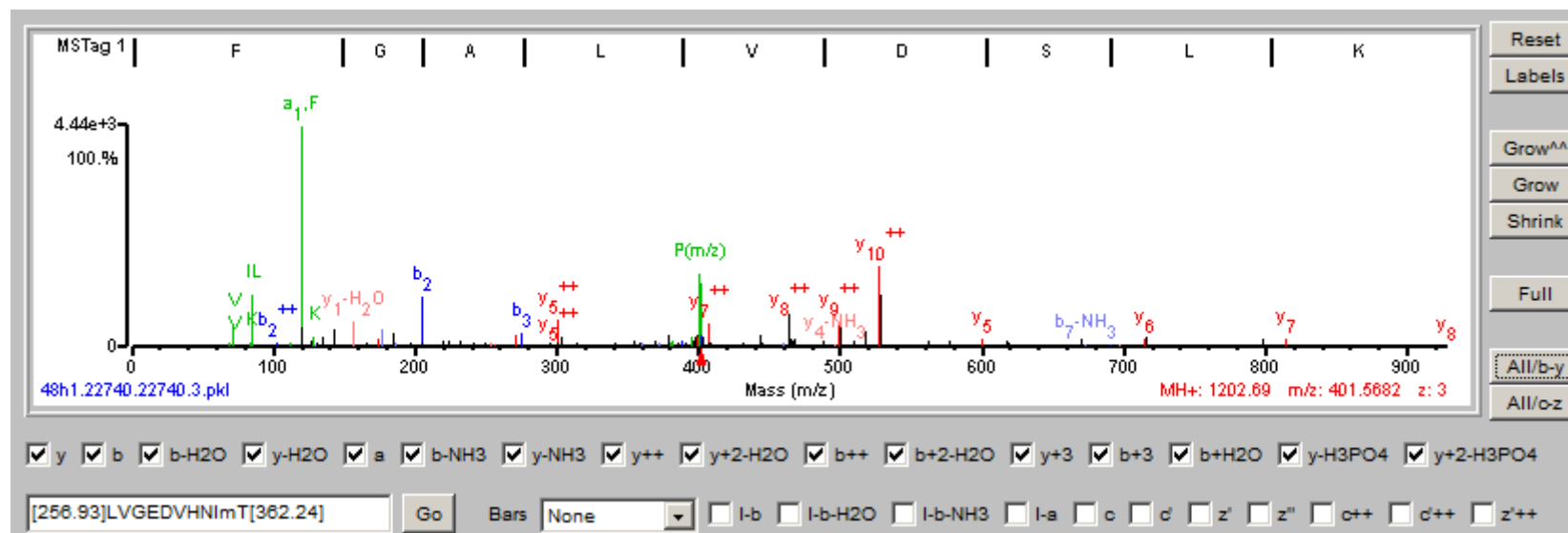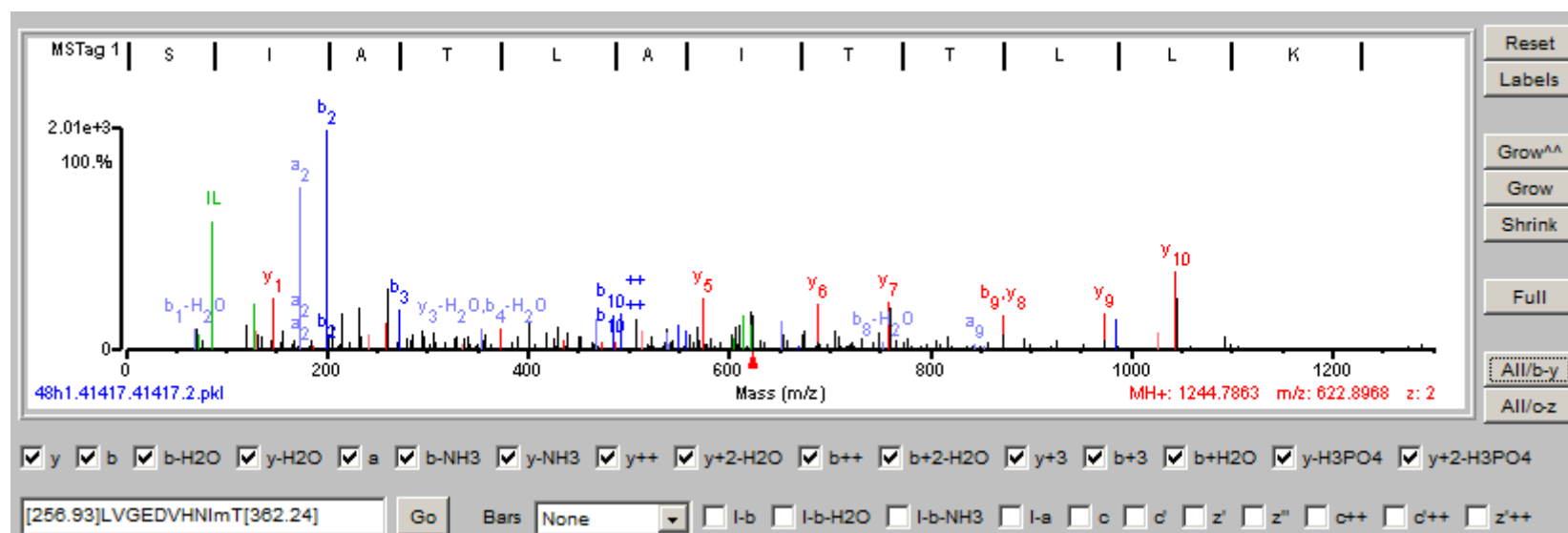

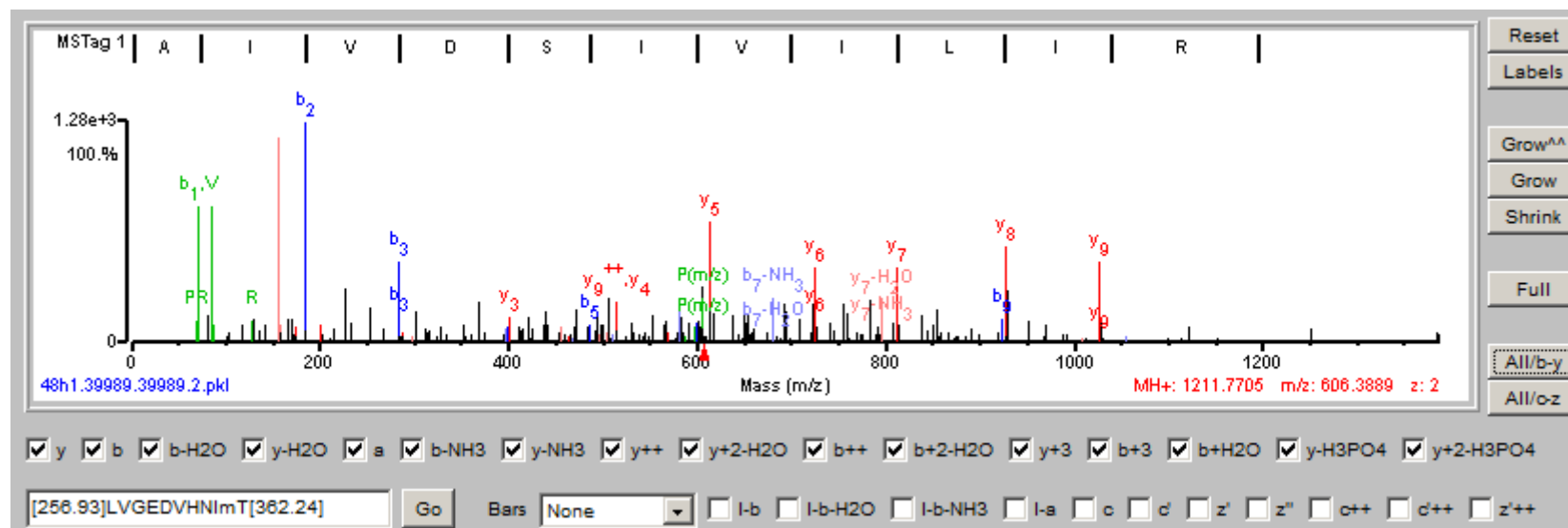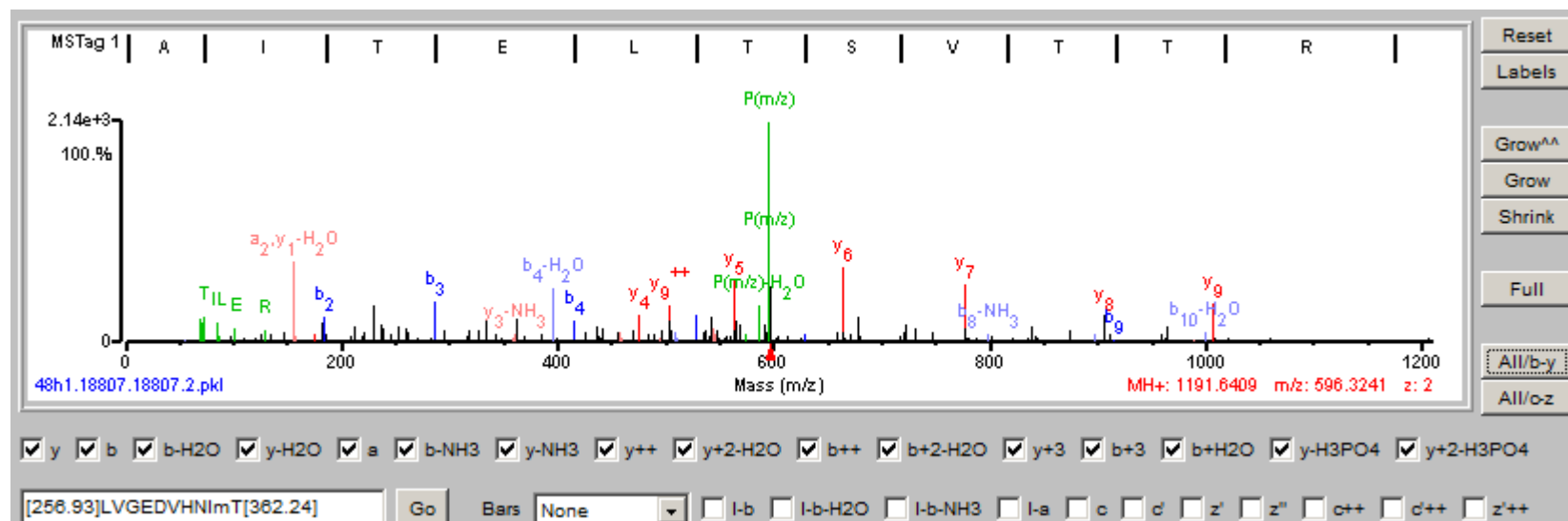

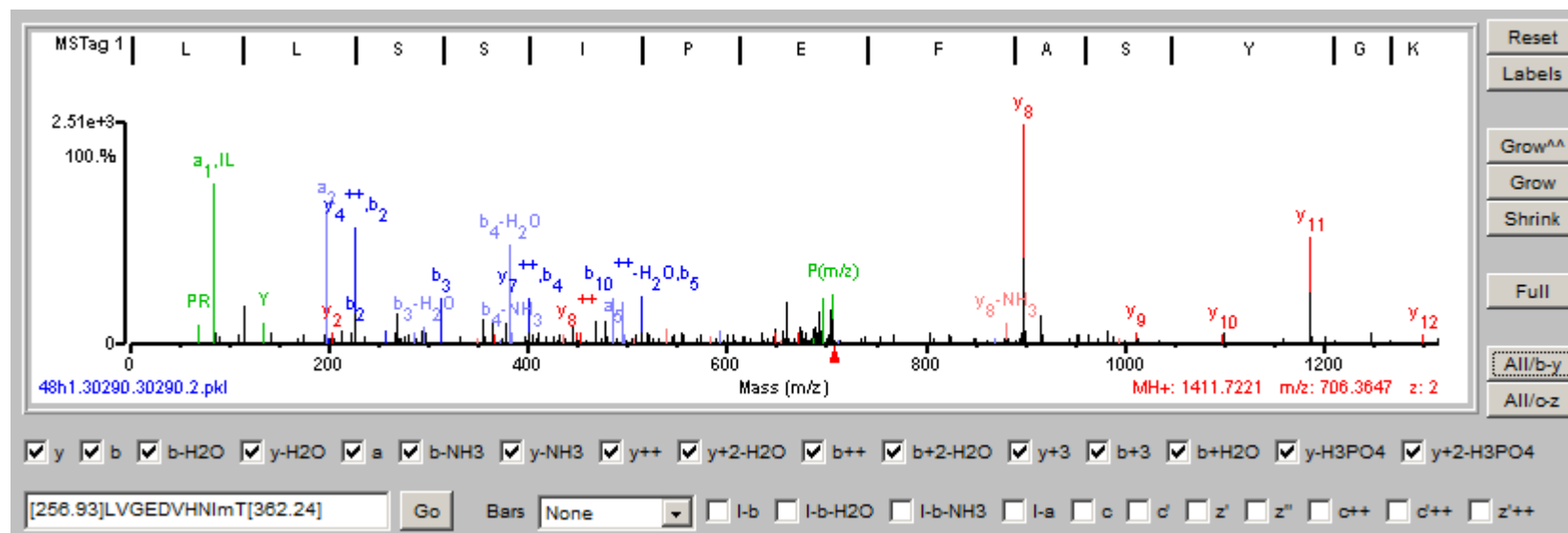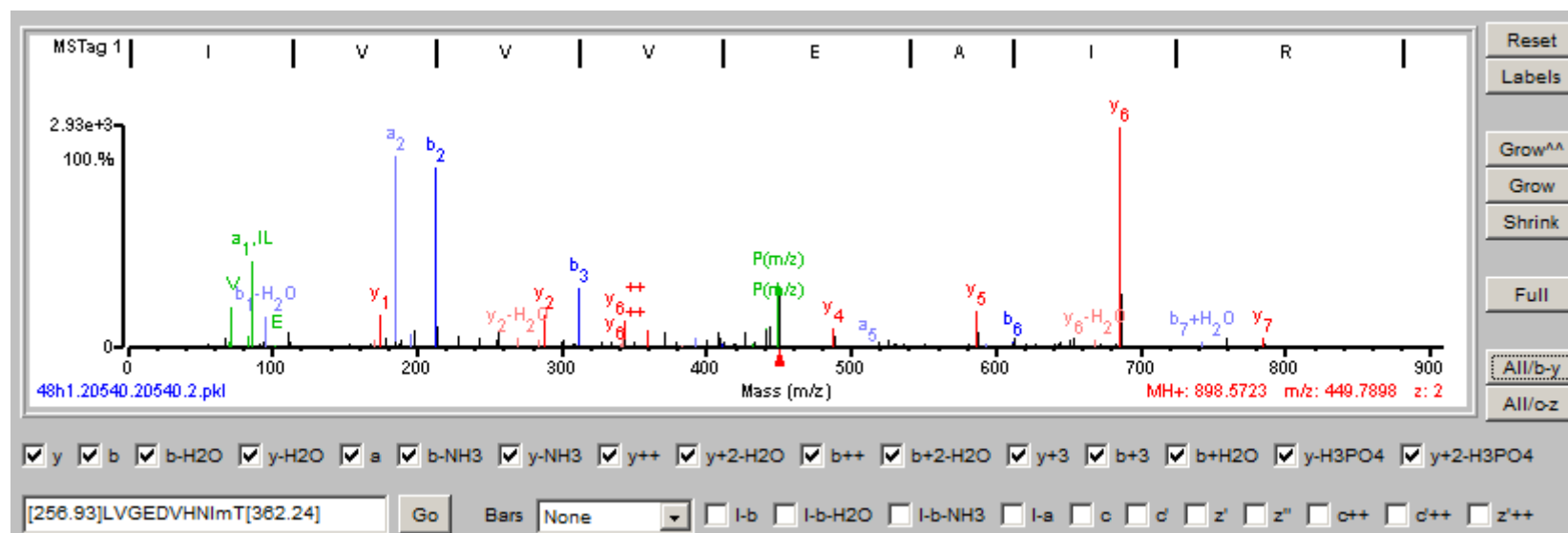

XP\_016551009.1

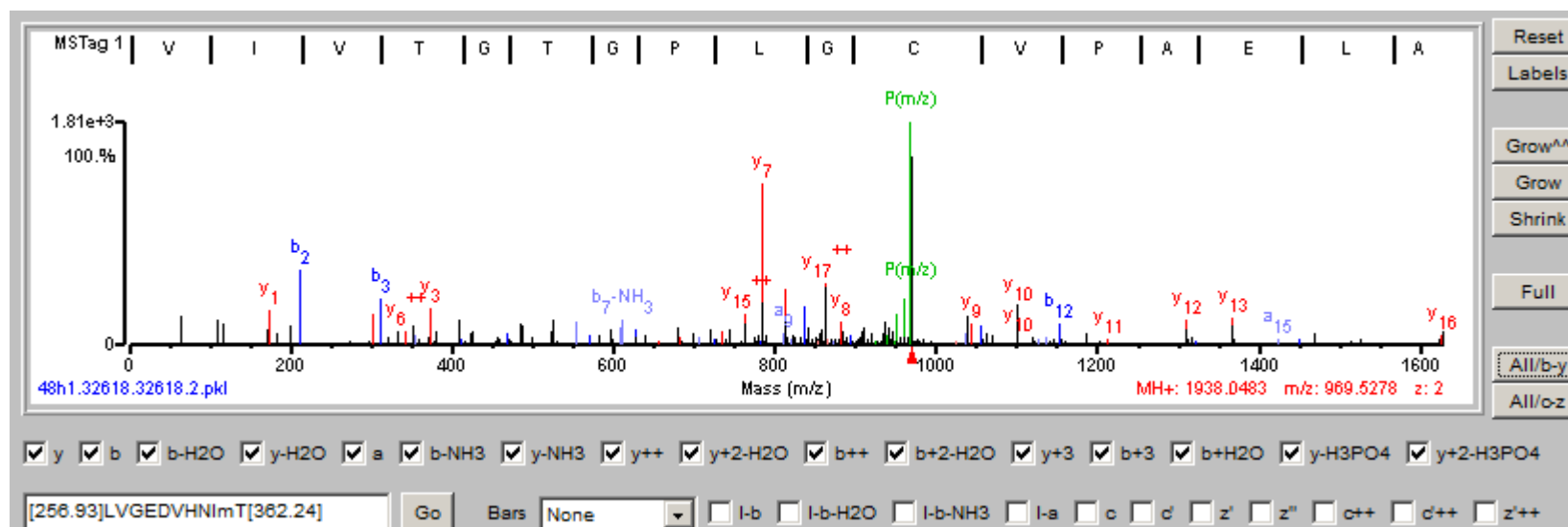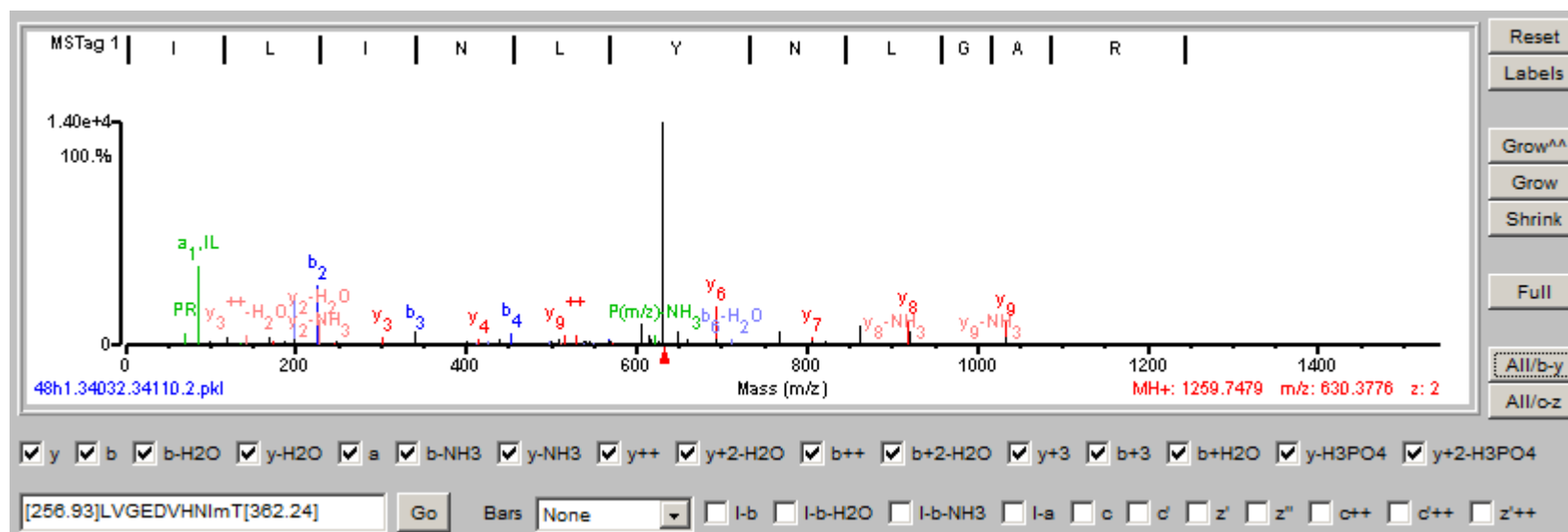

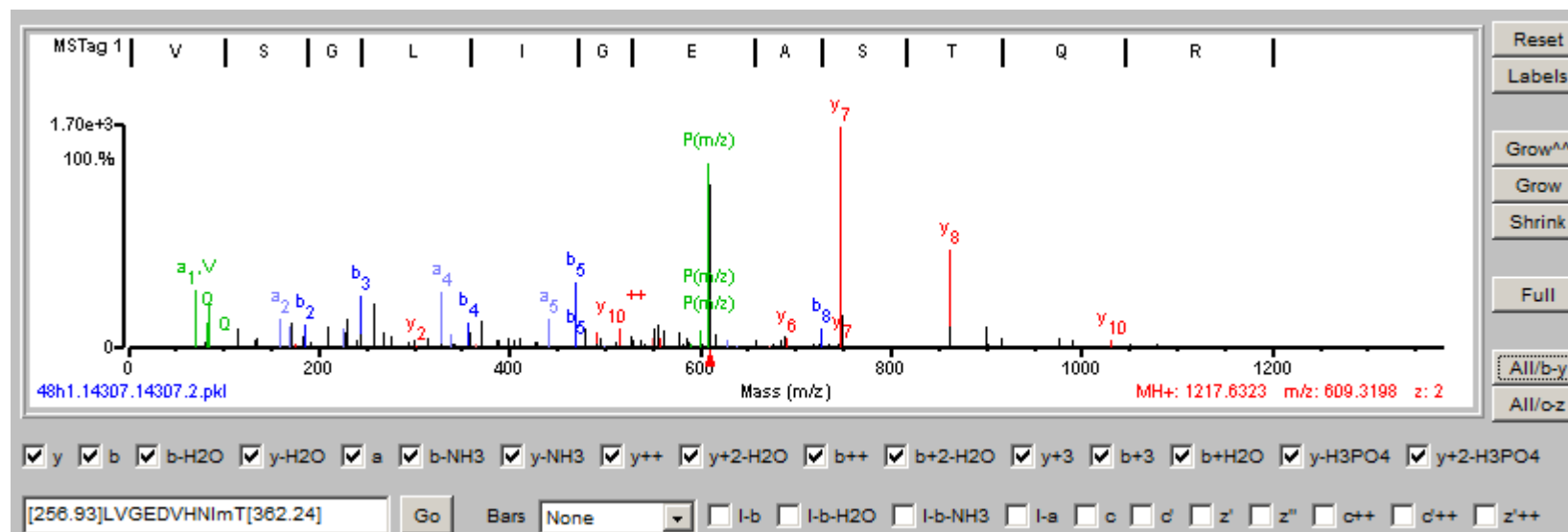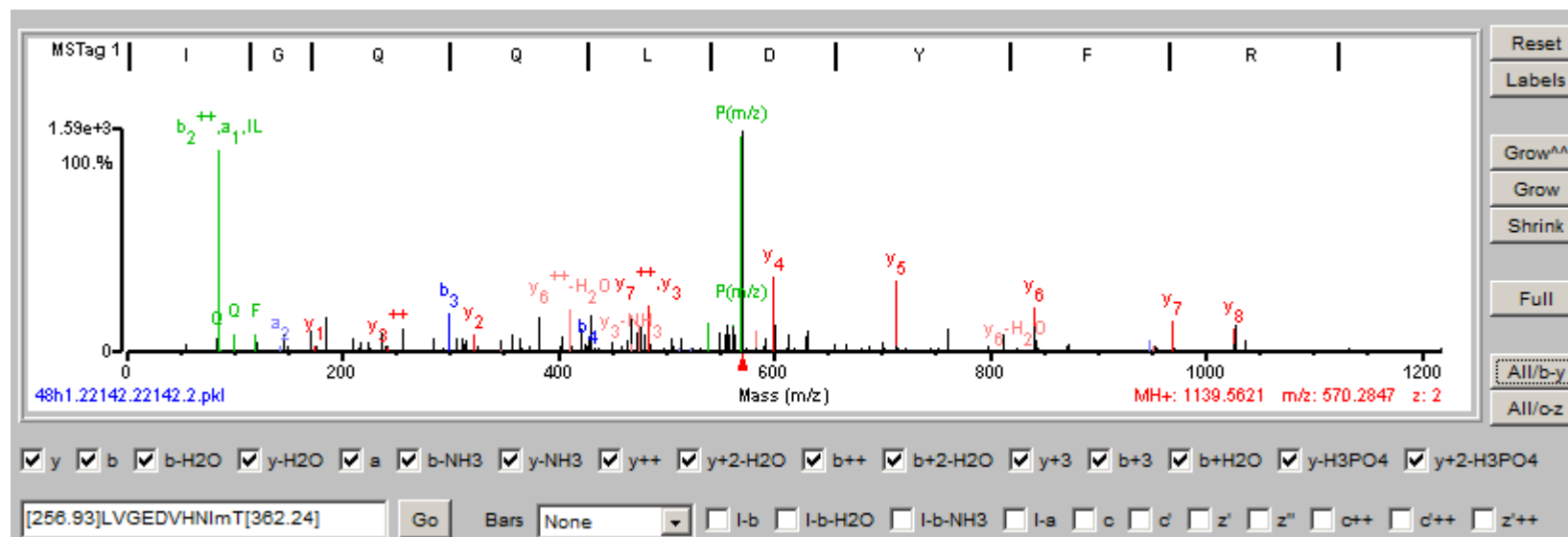

XP\_016551679.1

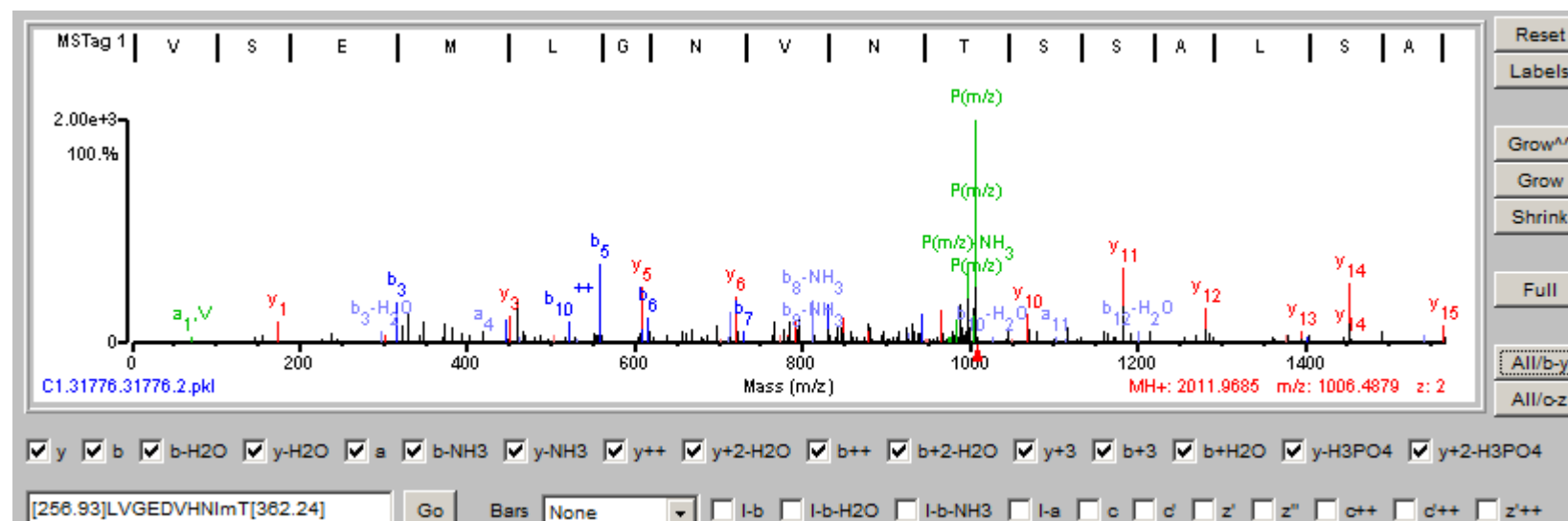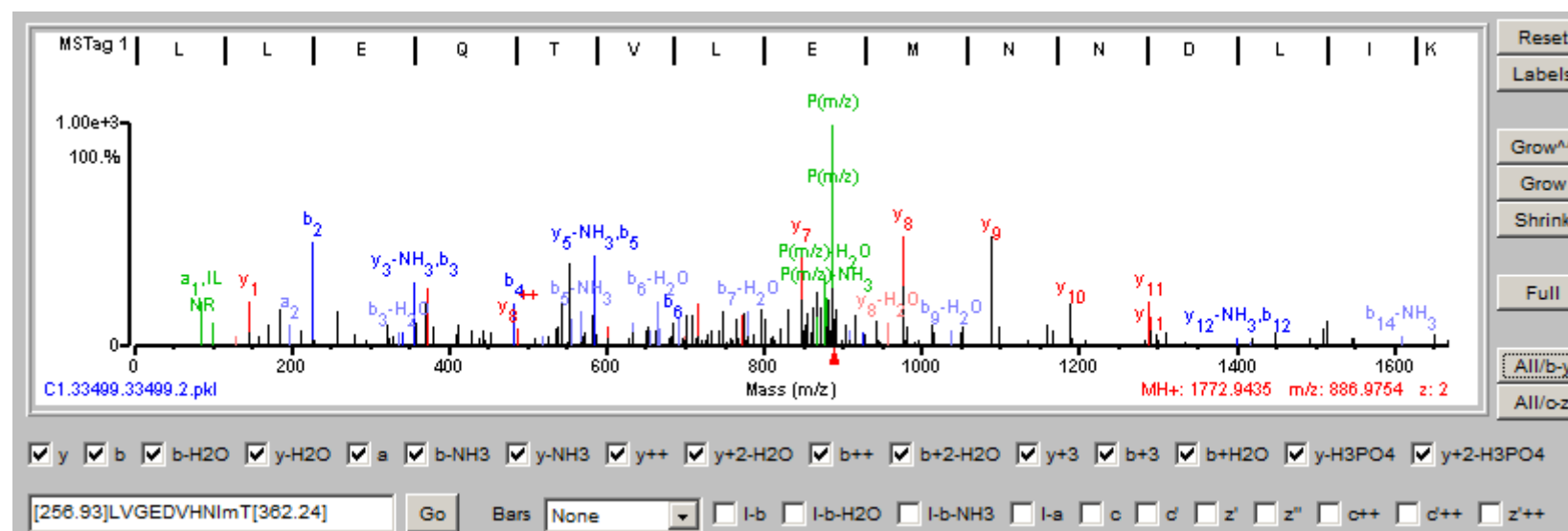

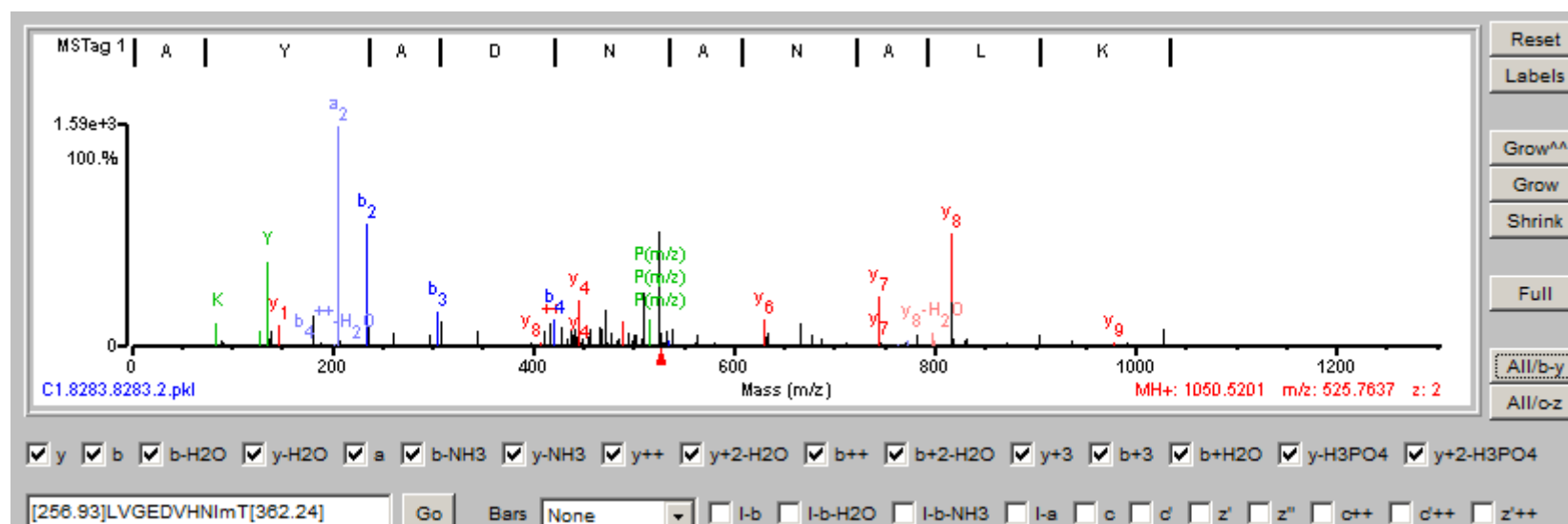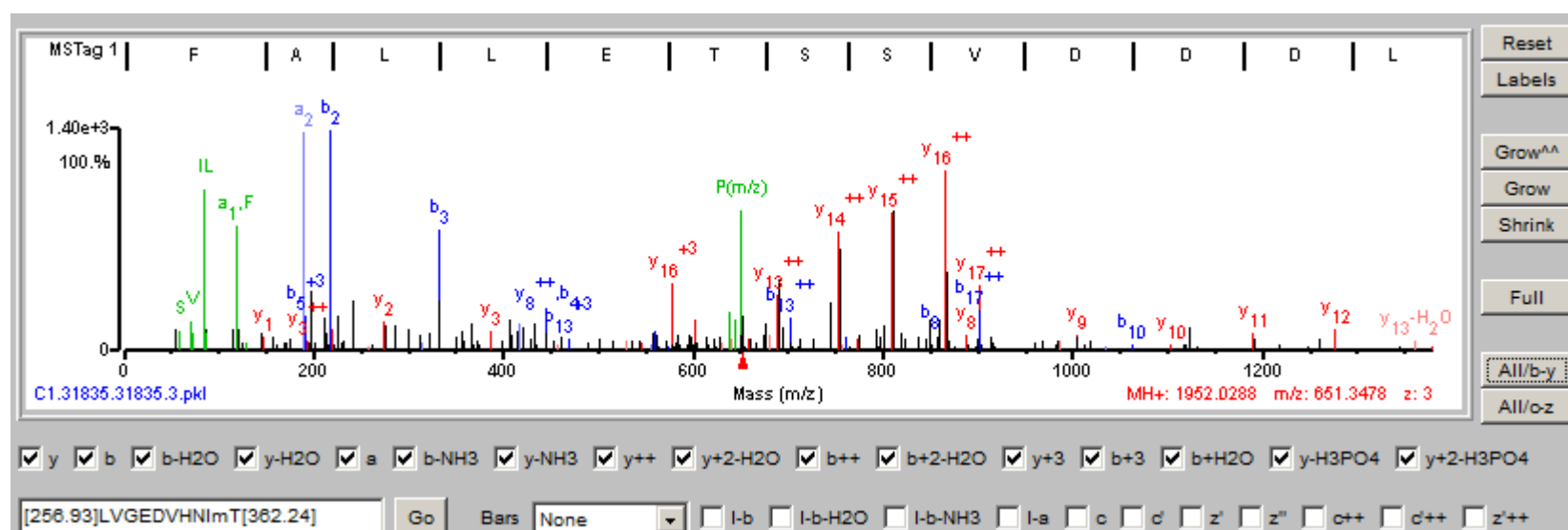

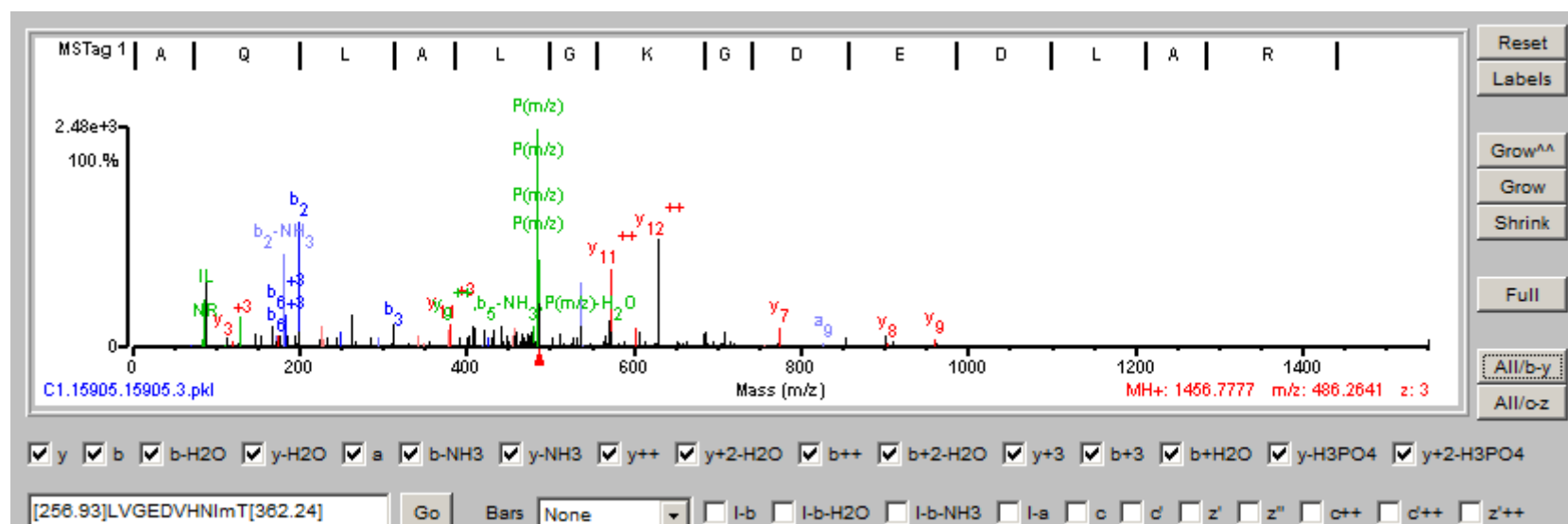

XP\_016563032.1

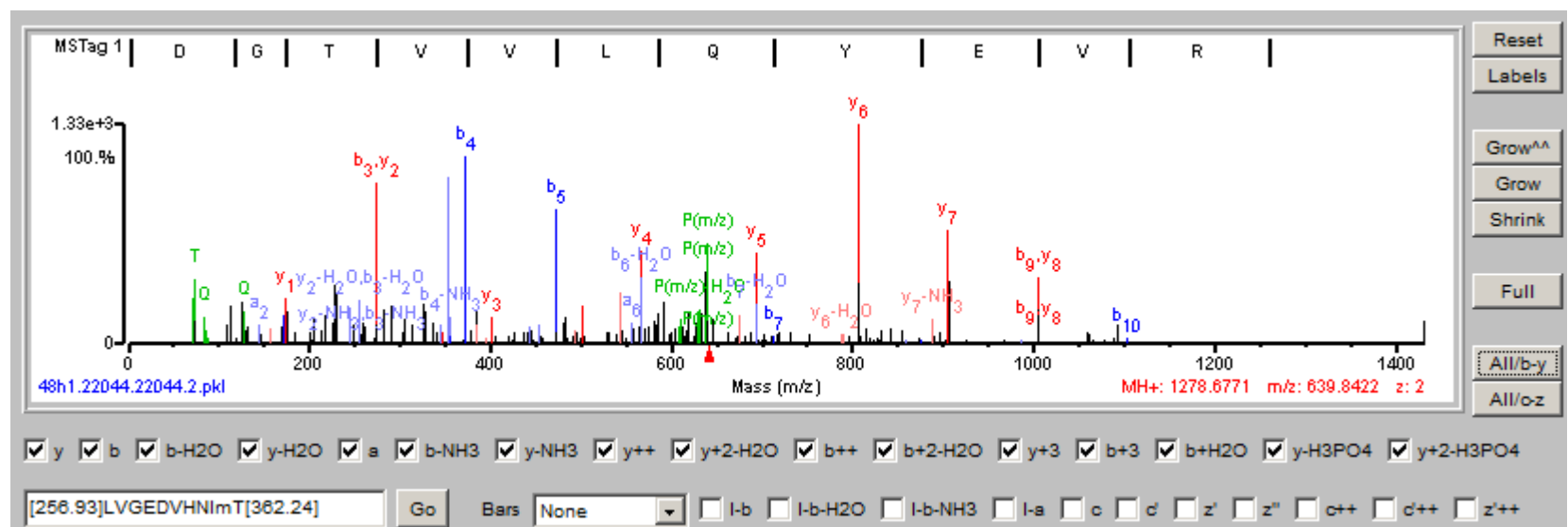

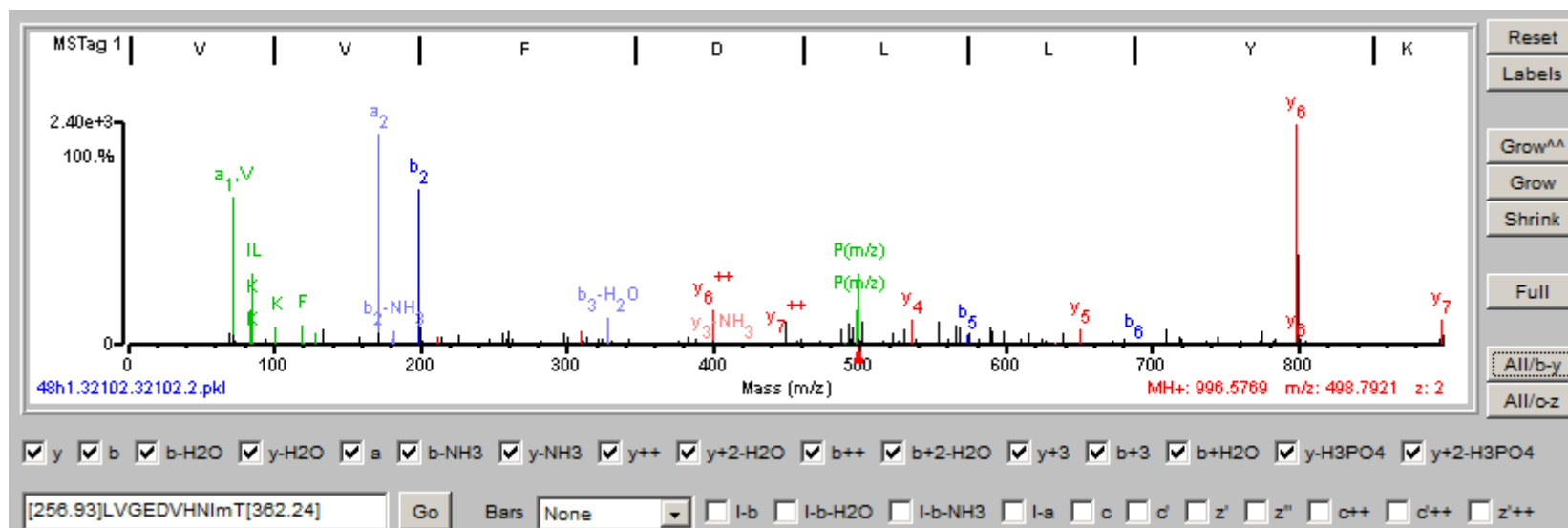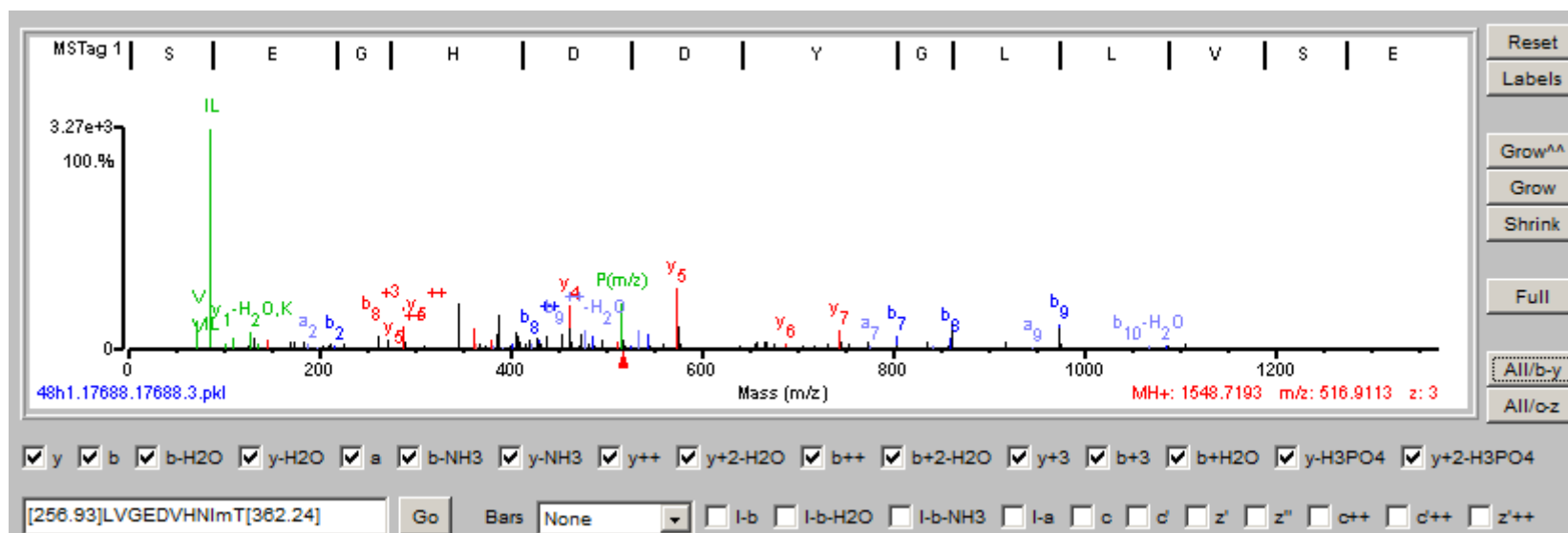

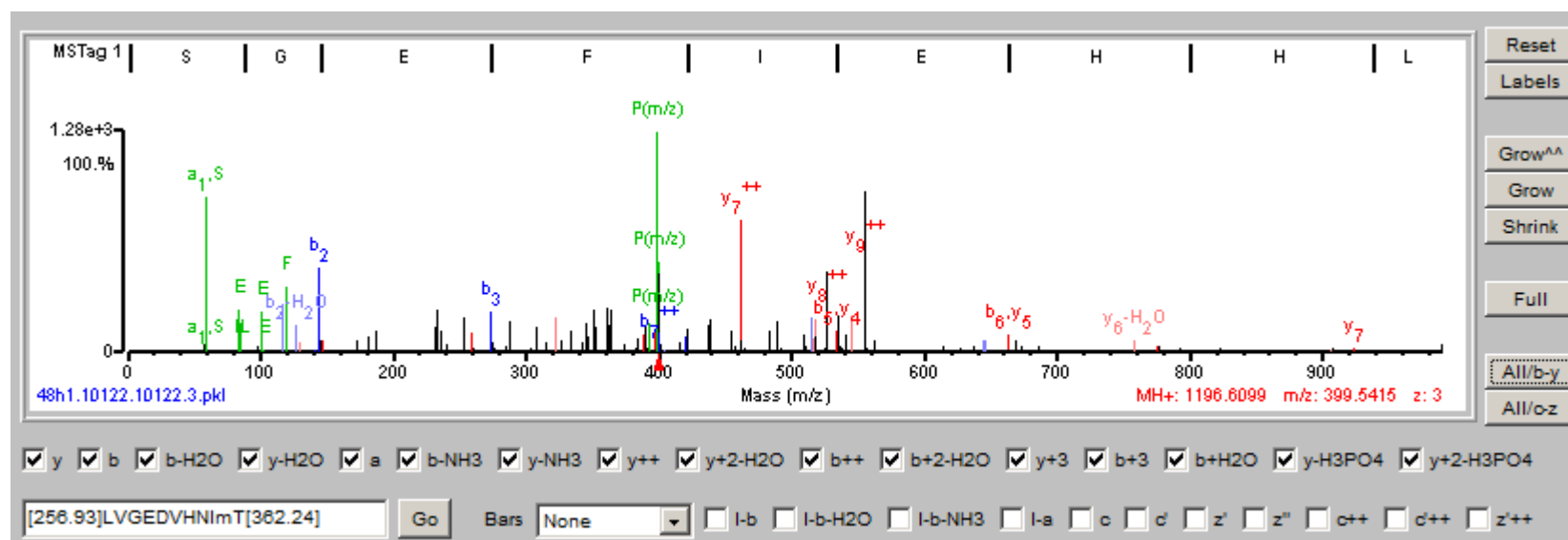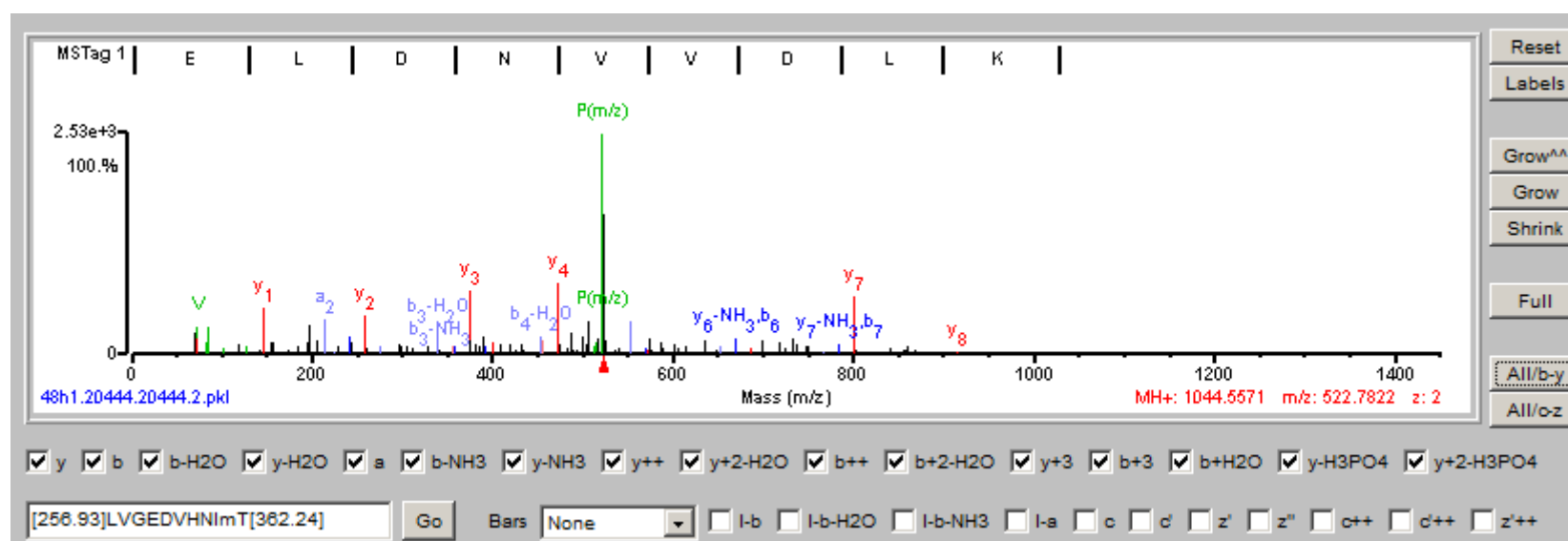

XP\_016563339.1

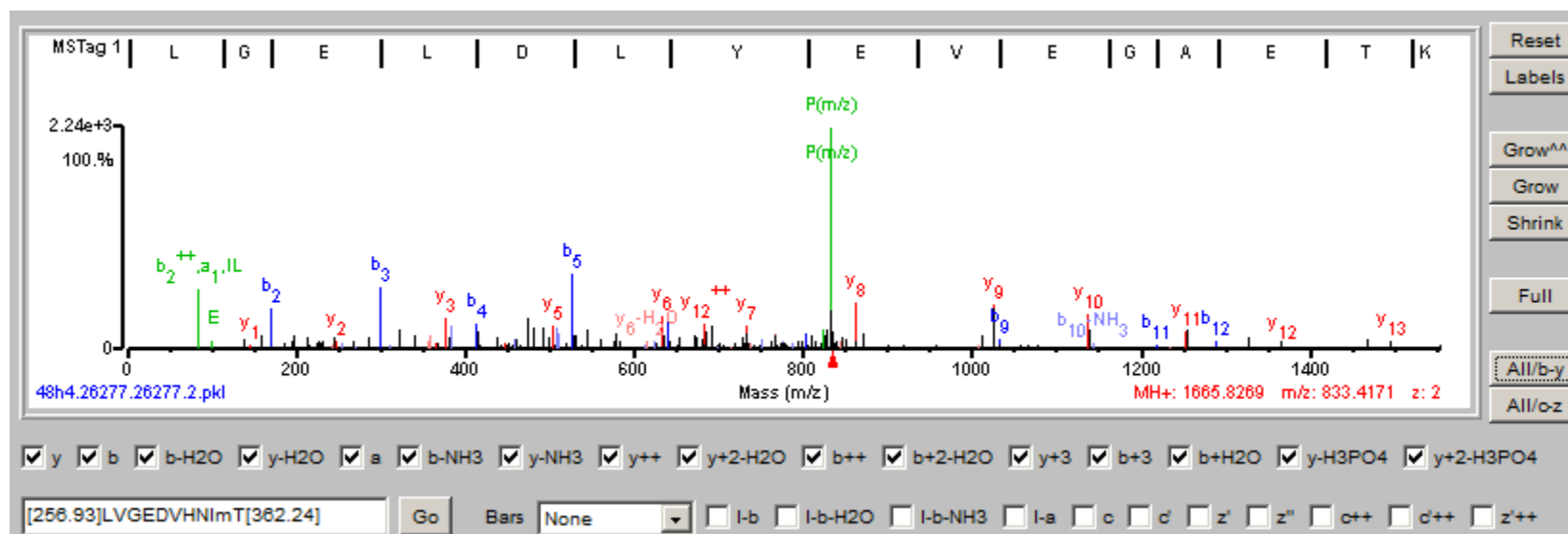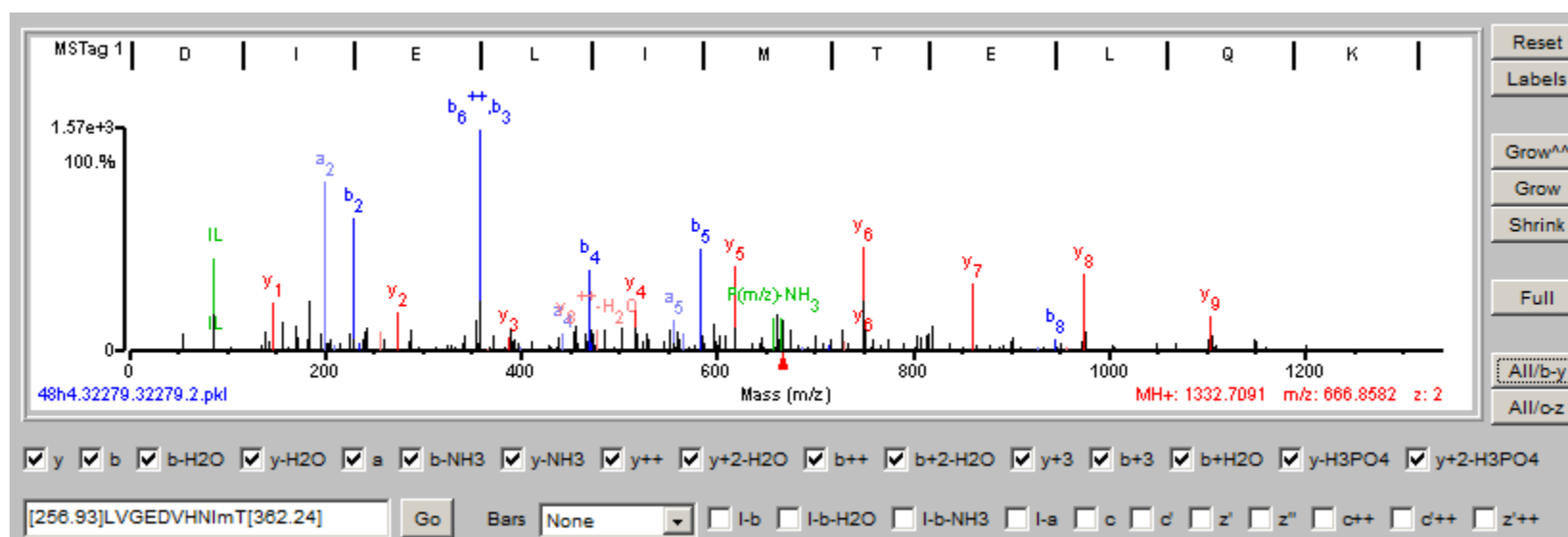

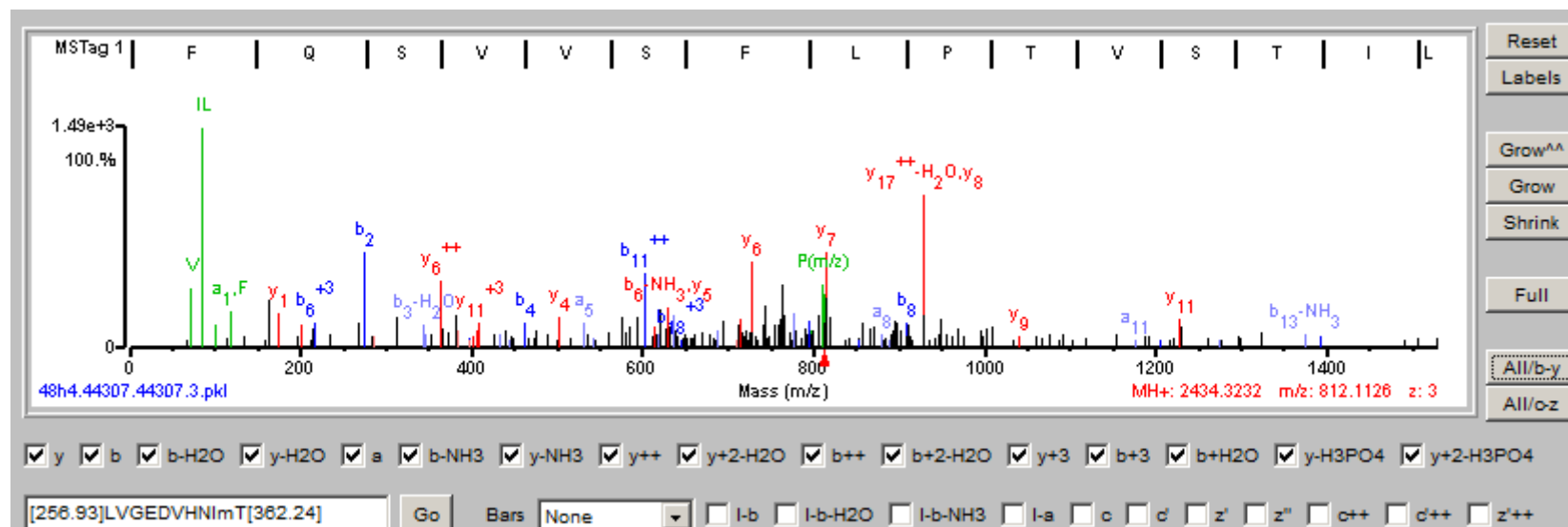

XP\_016566389.1

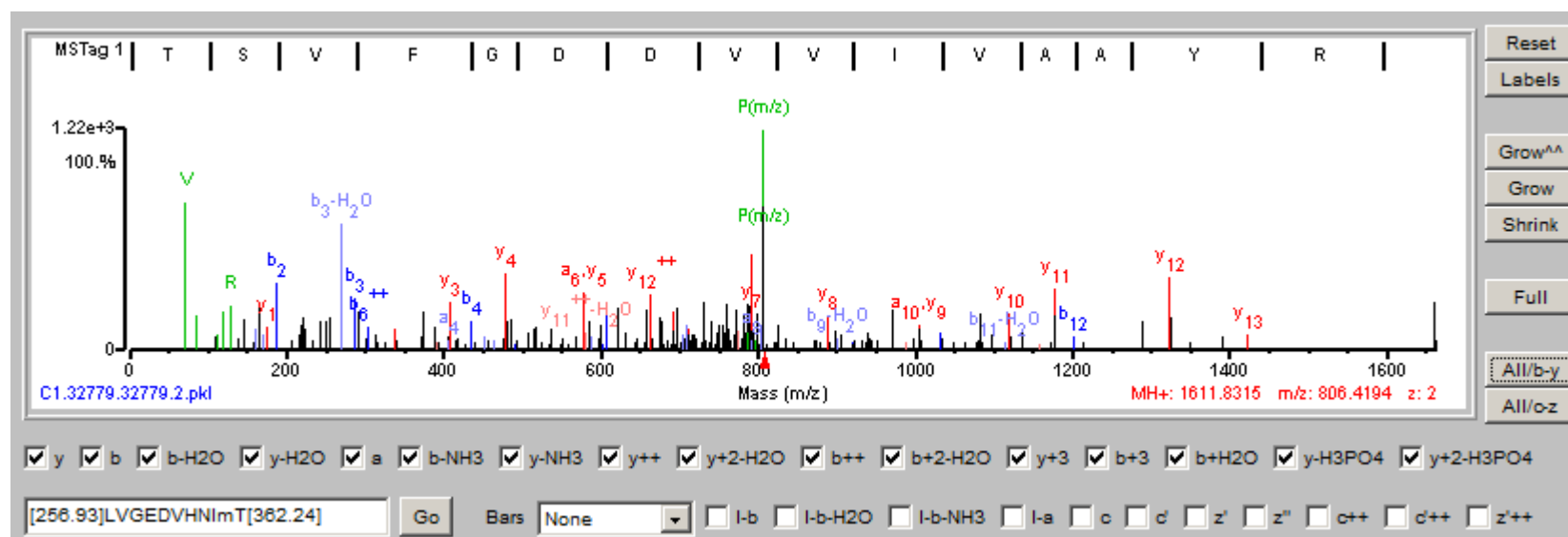

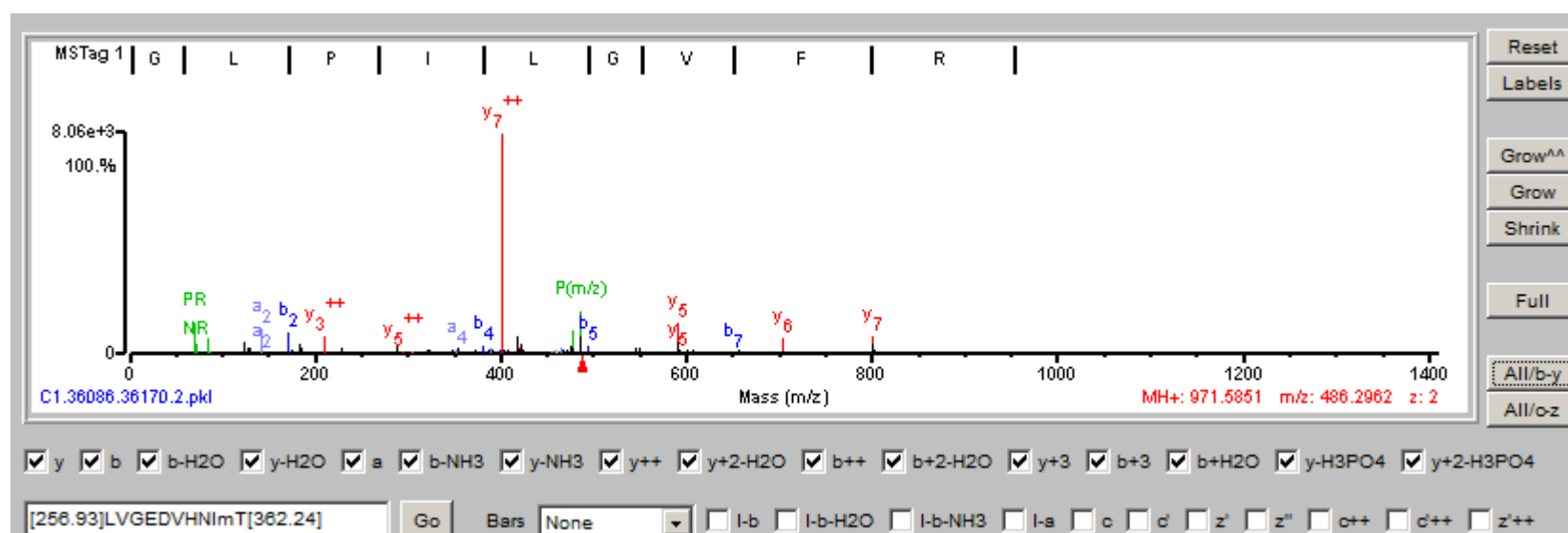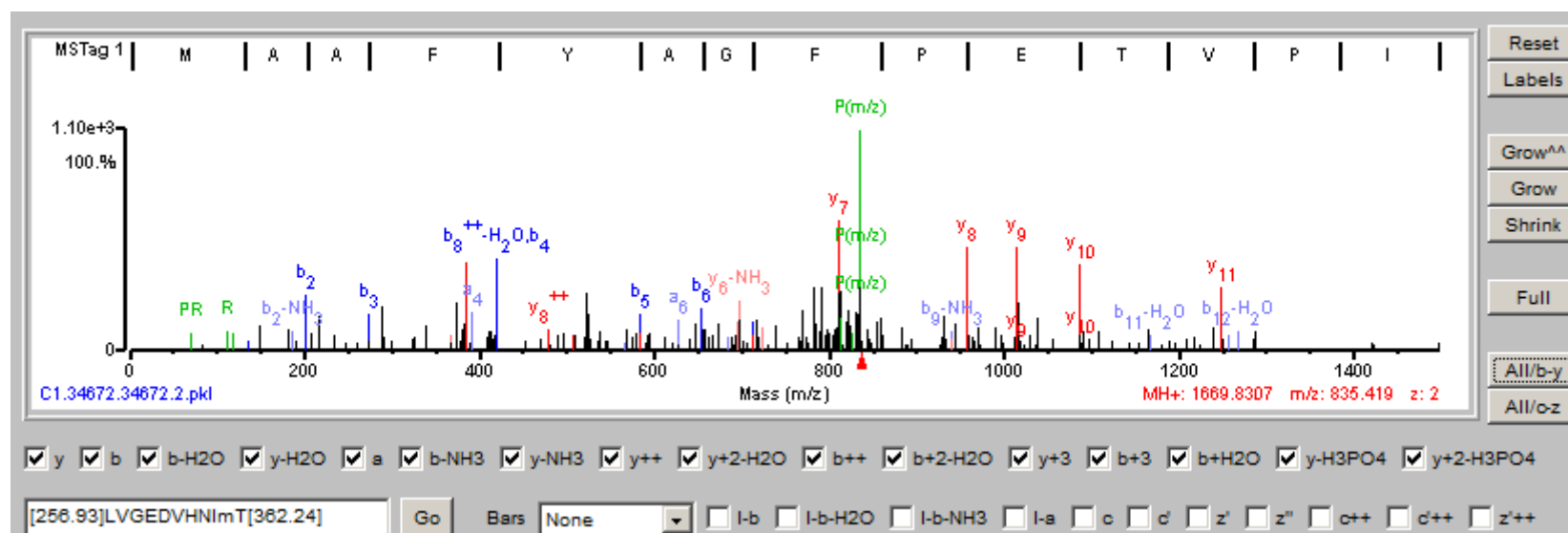

XP\_016574877.1

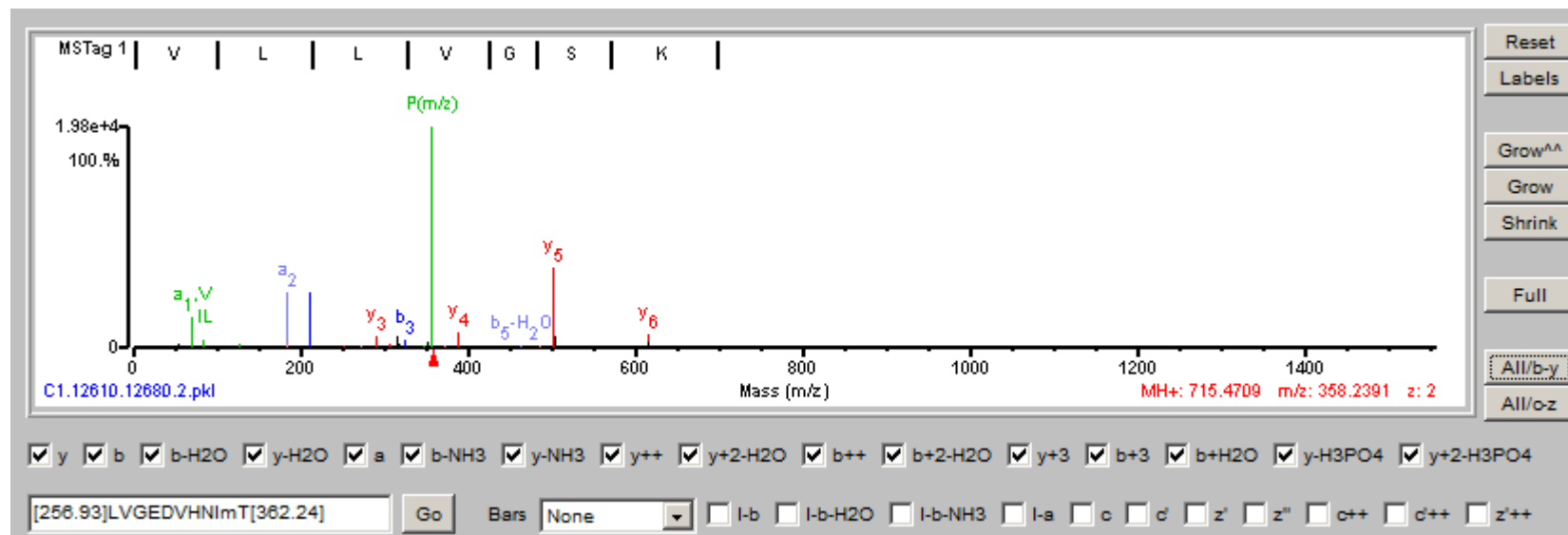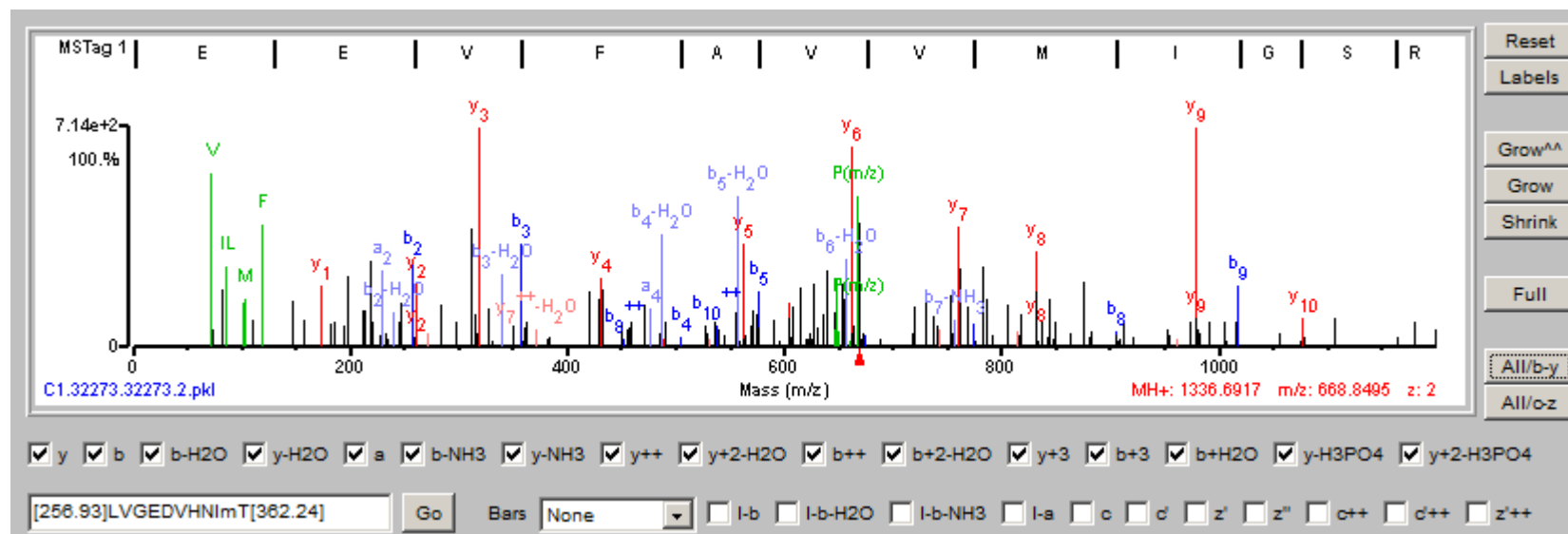

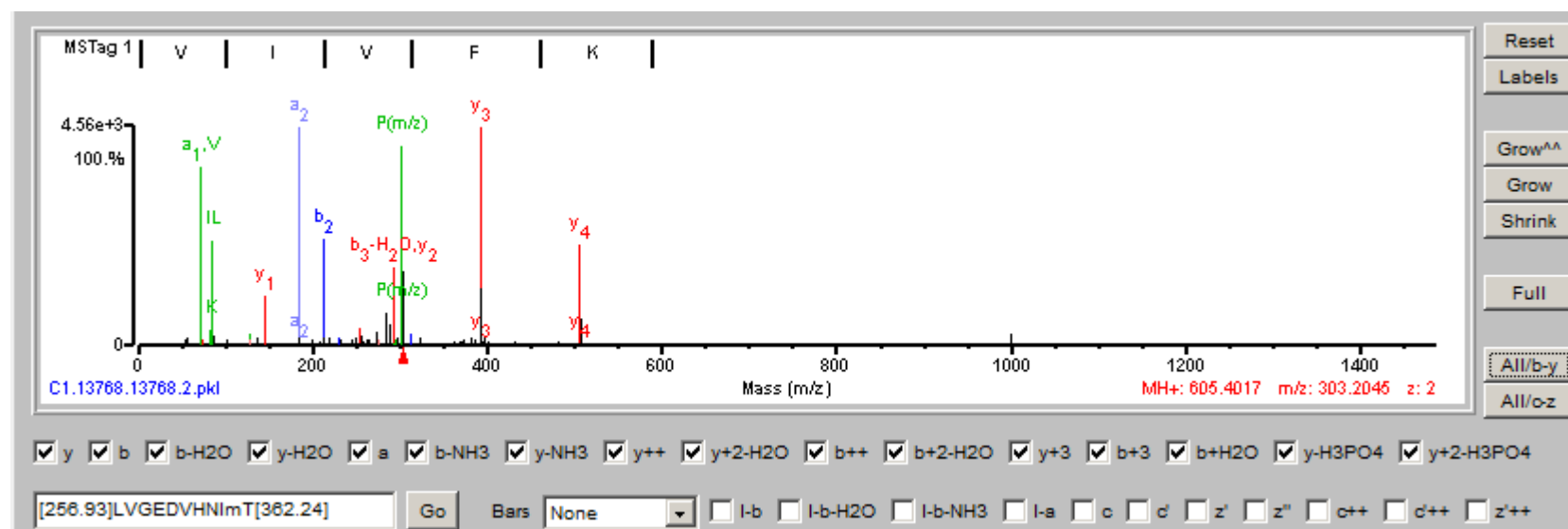

XP\_016577941.1

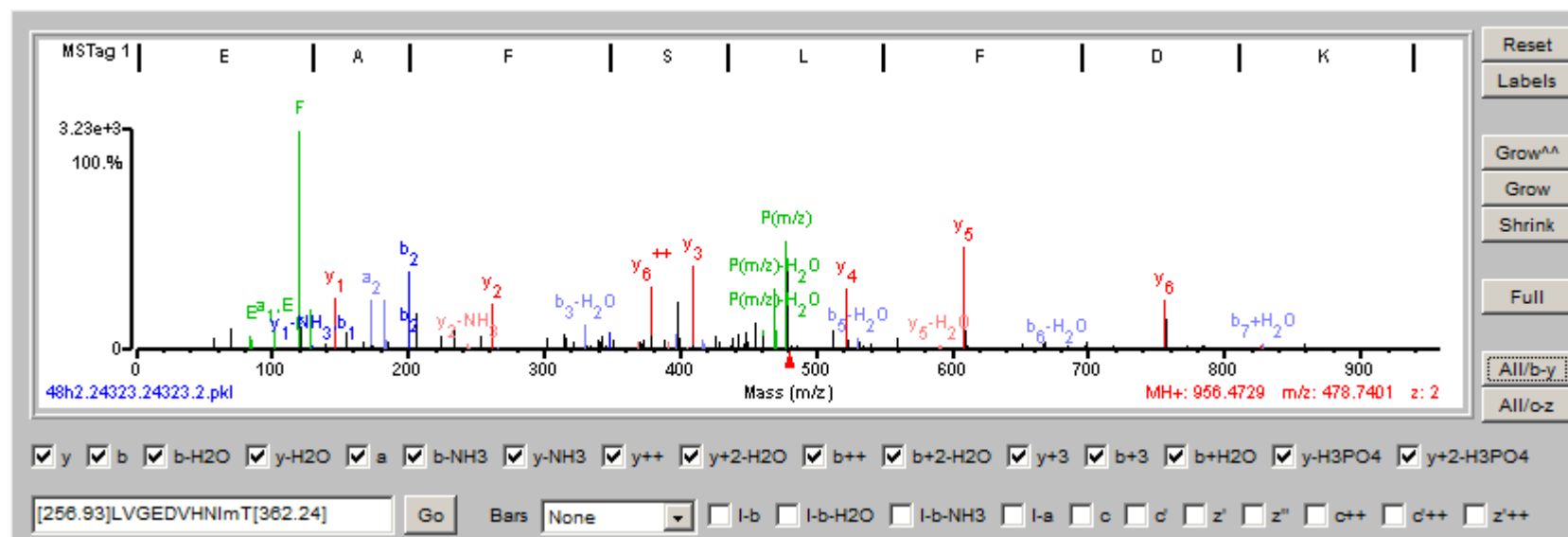

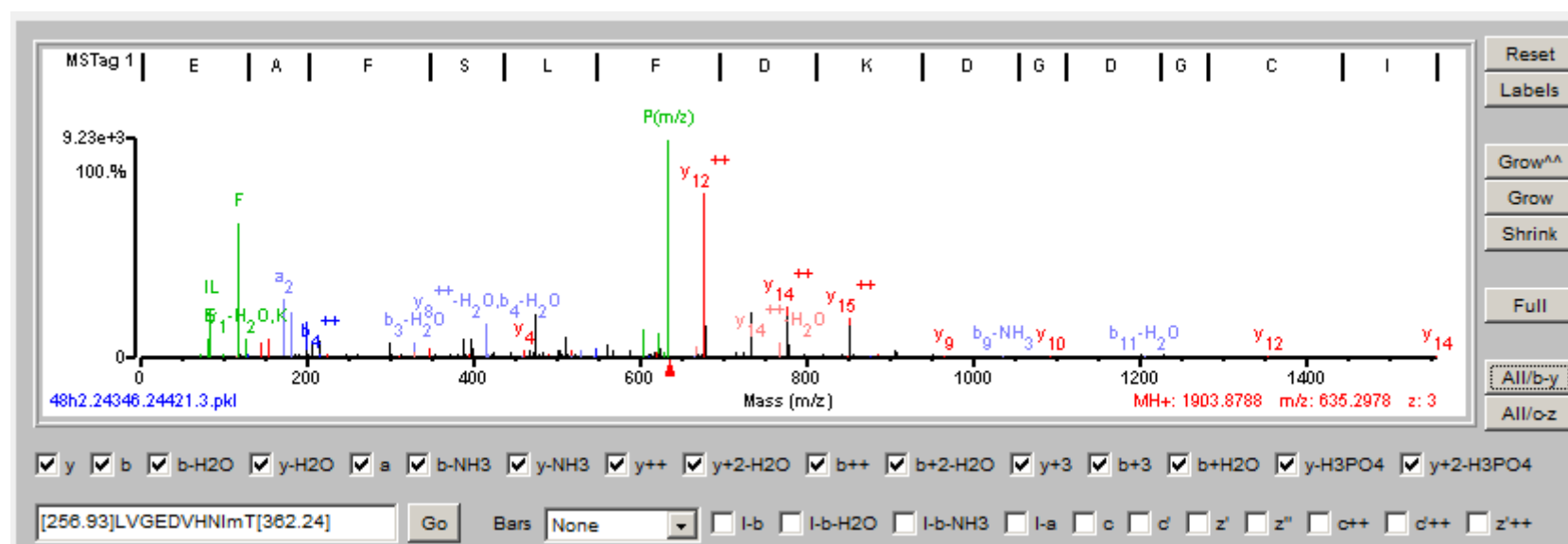

Supplement: Supplementary file 2 — Additional file 2. Some characteristic spectra of regulated proteins. [file 12870_2020_2749_MOESM2_ESM.pdf]
